# Common asymptomatic and submicroscopic malaria infections in Western Thailand revealed in longitudinal molecular and serological studies: a challenge to malaria elimination

**DOI:** 10.1186/s12936-016-1393-4

**Published:** 2016-06-22

**Authors:** Elisabeth Baum, Jetsumon Sattabongkot, Jeeraphat Sirichaisinthop, Kirakorn Kiattibutr, Aarti Jain, Omid Taghavian, Ming-Chieh Lee, D. Huw Davies, Liwang Cui, Philip L. Felgner, Guiyun Yan

**Affiliations:** 1 Department of Medicine, Division of Infectious Diseases, University of California Irvine, Irvine, CA USA; 2 Mahidol Vivax Research Unit, Faculty of Tropical Medicine, Mahidol University, Bangkok, Thailand; 3 Vector Borne Disease Training Center, Saraburi, Thailand; 4 Program in Public Health, University of California Irvine, Irvine, CA USA; 5 Department of Entomology, Pennsylvania State University, University Park, PA USA

**Keywords:** Asymptomatic, Submicroscopic, *Plasmodium vivax*, *Plasmodium falciparum*, Molecular screening, qPCR, Protein microarray, Serology, Surveillance, Low transmission

## Abstract

**Background:**

Despite largely successful control efforts, malaria remains a significant public health problem in Thailand. Based on microscopy, the northwestern province of Tak, once Thailand’s highest burden area, is now considered a low-transmission region. However, microscopy is insensitive to detect low-level parasitaemia, causing gross underestimation of parasite prevalence in areas where most infections are subpatent. The objective of this study was to assess the current epidemiology of malaria prevalence using molecular and serological detection methods, and to profile the antibody responses against *Plasmodium* as it relates to age, seasonal changes and clinical manifestations during infection. Three comprehensive cross-sectional surveys were performed in a sentinel village and from febrile hospital patients, and whole blood samples were collected from infants to elderly adults. Genomic DNA isolated from cellular fraction was screened by quantitative-PCR for the presence of *Plasmodium falciparum, Plasmodium vivax*, *Plasmodium malariae, Plasmodium ovale* and *Plasmodium knowlesi.* Plasma samples were probed on protein microarray to obtain antibody response profiles from the same individuals.

**Results:**

Within the studied community, 90.2 % of *Plasmodium* infections were submicroscopic and asymptomatic, including a large number of mixed-species infections. Amongst febrile patients, mixed-species infections comprised 68 % of positive cases, all of which went misdiagnosed and undertreated. All samples tested showed serological reactivity to *Plasmodium* antigens. There were significant differences in the rates of antibody acquisition against *P. falciparum* and *P. vivax*, and age-related differences in species-specific immunodominance of response. Antibodies against *Plasmodium* increased along the ten-month study period. Febrile patients had stronger antibody responses than asymptomatic carriers.

**Conclusions:**

Despite a great decline in malaria prevalence, transmission is still ongoing at levels undetectable by traditional methods. As current surveillance methods focus on case management, malaria transmission in Thailand will not be interrupted if asymptomatic submicroscopic infections are not detected and treated.

**Electronic supplementary material:**

The online version of this article (doi:10.1186/s12936-016-1393-4) contains supplementary material, which is available to authorized users.

## Background

In Thailand, control efforts have been highly effective in curbing malaria nationwide [1], but malaria remains a significant public health problem along the hilly and forested areas of the country’s borders with Myanmar and Cambodia [2–4]. High geographical heterogeneity in malaria endemicity and the presence of multiple *Plasmodium* species that cause human malaria (*Plasmodium falciparum*, *Plasmodium vivax*, *Plasmodium malariae*, *Plasmodium ovale* and *Plasmodium knowlesi*) are characteristics of malaria epidemiology in the region.

The Government of Thailand aims to achieve malaria elimination by 2030 [5], thus identification (including parasite speciation) and accurate treatment of asymptomatic and symptomatic infections are critical to accomplish this goal. However, this remains a major challenge because light microscopic analysis of blood smears, the gold standard in malaria diagnosis in Thailand, is insensitive at detecting low-level parasitaemia [6]. It is known that as malaria transmission declines, an increasing proportion of individuals are found to have asymptomatic and submicroscopic malaria infections [7], such as in many countries in Southeast Asia and the Amazon [8–12]. This is important because asymptomatic and submicroscopic malaria infections are known to contribute to transmission [7, 13, 14].

Our study site in the Myanmar-border province of Tak, once Thailand’s highest malaria burden region, experienced a drastic reduction in transmission recently [15, 16], and based on microscopy-estimated parasite prevalence (<1 %), it is now considered to be a low-transmission area. However, in our previous study in the Thai–Myanmar border area, a significant number of asymptomatic and submicroscopic malaria infections amongst the adult population were detected by quantitative polymerase chain reaction (qPCR) [17]. Moreover, amongst adult malaria patients attending a malaria clinic, a large number of cryptic mixed-species infections went undetected and untreated [17].

In the present study, the molecular screening and antibody profiling by microarray were expanded to include samples from all ages and three seasonal time points to obtain a more accurate assessment of the current epidemiology of falciparum and vivax malaria in Tak. For blood samples collected during community mass blood surveys and passive case detection activities at the hospital and clinic, the presence of all five *Plasmodium* species found in Thailand was investigated by qPCR. The population’s antibody responses against *P. falciparum* and *P. vivax* proteins were profiled to examine the development of antibody acquisition relative to age and season, and the targets of antibody responses in persons with asymptomatic and febrile malaria in an age-related manner.

## Methods

### Study sites and samples

The study was conducted in the village Mae Salid Noi (17° 28′ 4.7202″, 98° 1′ 48.5106″) and the town of Mae Tan (17° 13′ 49.0146″, 98° 13′ 55.6212″) in Tak Province, northwestern Thailand along the Thai–Myanmar border [17]. The sites are in a low and unstable transmission area, with higher transmission in the rainy season from May to October [3]. The four human malaria parasites, as well as the simian malaria species *P. knowlesi*, are known to infect humans in the region [18, 19], but *P*. *vivax* and *P. falciparum* are vastly predominant [16, 20, 21].

Whole blood samples were collected during three cross-sectional mass blood surveys (MBS) in the study village Mae Salid Noi, in March (*n* = 485) and August (*n* = 398) of 2013, and January 2014 (*n* = 464) from individuals ranging from 0.7 to 92 years of age (median, 14; mean 22, 95 % CI 21–23). The health history, including absence or presence of malaria symptoms as described below, was recorded weekly for each study participant from October 2012 to June 2014. The sample size (*n* = 1347) enabled us to determine parasite prevalence by qPCR with 2.6 % margin of error, using alpha 0.05.

Additionally, 297 whole blood samples were collected from individuals with suspected malaria during passive case detection (PCD) at the Mae Tan malaria clinic and hospital in March (*n* = 67) and August (*n* = 53) 2013, and January 2014 (*n* = 177). Age of PCD participants ranged from 0.8 to 84 years old (median, 23; mean 25, 23–27).

### Blood sample preparation for DNA analysis

From each study participant, 300 μL of whole blood was collected from finger prick into a Microvette CB300 capillary blood collector with lithium–heparin (Sarstedt, Newton, NC). Samples were centrifuged to separate cellular and plasma fractions, then immediately frozen at −80 °C for shipment to University of California Irvine for analysis. Upon thawing, plasma was removed and stored at −80 °C until use. Total genomic DNA was isolated from 100 μL of pelleted cellular fraction using DNeasy Blood and Tissue kit in the QIAcube automated system (Qiagen, Valencia, CA), using the Blood and Body Fluid Spin Protocol with Manual Lysis V1. Purified genomic DNA samples were eluted with 200 μL of Buffer AE and kept at −20 °C until use.

### Sample analysis by microscopy and quantitative PCR

Field microscopy was performed by local trained staff who provided the first result, and positive cases were treated per national malaria treatment guidelines. Individuals found positive in subsequent expert microscopy and molecular screenings were not treated. Expert microscopic examination was conducted at Mahidol University by an expert microscopist with over three decades of experience. Molecular detection of *Plasmodium* parasites was performed at University of California Irvine using a two-tier strategy for qPCR consisting of an initial screening of all 1644 samples for the presence of *Plasmodium* genus-specific products using SYBR Green, followed by TaqMan assays to determine the *Plasmodium* species in samples positive in the genus-specific assay.

For the *Plasmodium*-genus screening, primers were designed to hybridize with a region of 18S rRNA gene conserved amongst *P. falciparum* (Pf), *P. vivax* (Pv)*, P. malariae* (Pm)*, P. ovale* (Po) and *P. knowlesi* (Pk). The forward primer sequence was 5′-GTATTCAGATGTCAGAGGTG-3′, and the reverse primer was 5′-CCTACTCTTGTCTTAAACTAGT-3′. Amplification was performed in 20 μL reactions containing 2 μL of genomic DNA, 10 μL FastStart SYBR Green qPCR Master Mix (Roche, Indianapolis, IN), 0.2 μM of each primer and 3 mM MgCl_2_, in a CFX96 Touch Real-Time PCR Detection System (BIORAD, Hercules, CA). After initial denaturation at 95 °C for 10 min, 45 cycles of 94 °C for 30 s, 60 °C for 30 s, 68 °C for 1 min were followed by a final step of 95 °C for 10 s and a melting curve from 65 to 95 °C with 0.5 °C increments for 5 s. Samples were considered positive if Cq was smaller than 41 and there was a product with melt peak in the temperature ranging between 74 and 75.5 °C. All assays included positive and negative controls. The detection limit of this method was determined to be 0.05 parasites/μL using serially diluted *P. falciparum* cultures.

Samples positive in the genus screening were examined in uniplex TaqMan assays using Plasmo 1 and Plasmo 2 primers and species-specific probes for *P. falciparum*, *P. vivax, P. malariae* and *P. ovale* as described in Rougemont et al. [22] and for *P. knowlesi* as described in Divis et al. [23], with the modification that probes were either FAM/ZEN/Iowa Black FQ (for Pf, Pv and Pk reactions) (Integrated DNA Technologies, San Diego, CA) or FAM/MGBNFQ (for Pm and Po reactions) (Applied Biosystems, Foster City, CA). Amplification was performed in 20 μL reactions containing 2 μL of genomic DNA, 10 μL TaqMan Universal Master Mix II, no UNG (ThermoFisher, Grand Island, NY), 0.2 μM of each primer and 80 ηM of probe, in a CFX96 Touch Real-Time PCR Detection System (BIORAD, Hercules, CA). Samples were tested in duplicate and considered positive if it generated a Cq value smaller than 40.

### Sample classification

Samples were classified into four major categories, according to collection site and presence or absence of *Plasmodium* DNA by qPCR. Samples collected at Mae Salid Noi village during community MBSs were classified as (1) *community malaria* (if sample was qPCR-positive for *Plasmodium*), or (2) *community healthy* (if sample was qPCR-negative). Samples collected at the hospital/malaria clinic from suspected malaria patients were classified as (3) *febrile malaria* (if qPCR-positive), or (4) *non*-*malaria illness* (if qPCR-negative). Malaria symptomatology is defined as fever (>37.5 °C), fatigue, myalgia, headache and nausea, occurring alone or in combination.

### *Plasmodium falciparum* and *P. vivax* protein microarray

The protein microarray used in this study, named Pf/Pv500 (Antigen Discovery Inc., Irvine CA), was described in detail in Baum et al. [17], and on NCBI’s Gene Expression Omnibus under platform accession number GPL21194. Briefly, the array includes 500 *P. falciparum* and 515 *P. vivax* polypeptides printed as in vitro transcription translation (IVTT) reactions, that were down-selected from larger microarray studies based on seroreactivity and antigenicity to humans. Gene accession numbers follow annotation published on PlasmoDB [24]. For large proteins printed on the microarray as overlapping polypeptides or individual exons, the exon position relative to the full molecule and the segment of the ORF are indicated where applicable.

### Probing of plasma samples on the Pf/Pv500 microarray

From the community MBS, 298 plasma samples were probed: 41 community malaria, and 257 community healthy samples, including the corresponding longitudinal samples of community malaria samples, and samples from individuals age-, gender-, and season-matched to the community malaria samples. Of these, 150 samples collected in March 2013 were randomly selected for the seroconversion study, 15 samples for each of 10 age groups. Additionally, 83 samples collected during PCD at the malaria clinic and hospital were probed, including 32 malaria fever and 51 age- and gender-matched non-malaria illness patients. As unexposed controls, 16 samples from healthy blood donors from the United States, with no travel history to malaria endemic regions, were used for serology comparisons. All microarray probing was performed on the same day, and as previously described in Baum et al. [17].

### Data analysis

For analysis of antibody binding to *P. falciparum* and *P. vivax* polypeptides on the microarray the following steps were taken: (1) the median background signal of antibody binding to 24 spots of IVTT reaction without DNA template (no template control, NTC) was calculated for each individual sample; (2) the raw values of antibody binding to *P. falciparum* and *P. vivax* polypeptides were divided by their corresponding median NTC value, generating fold-over-control (FOC) values; (3) FOC values were log_2_-transformed for data normalization. Normalized data was used for statistical analyses and for figure representations of the data. (4) To determine which polypeptides were seroreactive to plasma from the Thai sample cohort, Significance Analysis for Microarrays (SAM) [25] was performed comparing the intensity of antibody binding to the proteins on the array between the samples collected in Thailand from individuals over 15 years-old (*n* = 211) and 16 USA unexposed controls. The test was performed using MeV 4.8.1, with the following parameters: median and 90th percentile of false discovery rate (FDR), 1.2 and 9.3 %, respectively; median and 90th percentile of number of false significant genes, 3.9 and 30.5, respectively. This resulted in 326 polypeptides being considered significantly seroreactive in exposed Thai samples, and all further analyses considered only this set. Individual plasma samples were considered seropositive for a polypeptide if the sample’s signal intensity value was above the upper 99 % confidence interval value of the unexposed control group. Breadth of response was determined by the number of antigens an individual or group of samples were seropositive to, based on the above criteria for seropositivity. For analysis of antibody responses, the non-parametric multiple comparison Steel–Dwass and the Wilcoxon test were used for pairwise comparisons of means, using JMP11.2. Significance tests were 2-sided and set at 0.05 level for type I error. Z-scores were calculated as the number of standard deviations above or below the mean of the unexposed group. Annual rates of seroconversion and seroreversion for the immunogenic polypeptides were calculated using Systat 11 (Systat Software, Chicago, IL) by fitting age-specific seroprevalence data to a reversible catalytic model using the maximum-likelihood method that assumes binomial error distribution: $P_{t} = \lambda /\lambda + \rho (1 - e( - (\lambda + \rho )*t))$ [26]; where *P*_*t*_ is the proportion of seropositive individuals in each age group *t*, λ is the annual rate of seroconversion and ρ is the annual rate of reversion to seronegativity. The model was fitted to seroprevalence curves to each seroreactive antigens using ten age groups, shown in Additional file 1. Individuals below 2 years of age were excluded to eliminate pre-existing maternal antibodies from analyses. *T* test for comparison of slopes of linear regression lines was performed as per Zar [27]. A two-proportion Z-test was used to compare the proportion of samples in different age categories between two populations. The geometric mean value and the 95 % confidence interval of data are reported in parenthesis, i.e. (0.73, 0.72–0.74).

## Results

### Infection rates and comparison between qPCR and microscopy results

From the community of Mae Salid Noi, DNA of *Plasmodium* parasites was detected by qPCR in 41 (3.0 %) of 1347 blood samples tested. The highest infection rate was observed in March 2013, with 4.3 % positive samples (21/485), and decreased significantly in August to 1.3 % (5/398), and then increased to 3.2 % in January 2014 (15/464). Four individuals had confirmed malaria in repeat samples, 5 (*P. falciparum*, *n* = 1) and 10 (*P. vivax*, *n* = 3) months apart. Of the 41 community malaria cases, three (two children and one adult) (7.3 %) presented fever at sample collection, while 33 (80.5 %) were asymptomatic and had no malaria symptoms or illness complaints during the 5-month period preceding and following sample collection. The remainder five (12.2 %) developed fever 1–4 months after presenting a positive qPCR for *Plasmodium* parasites.

Table 1 shows a comparison of results from molecular and microscopy screening for *Plasmodium* parasites for the samples collected. Amongst samples from the community surveys, *P. vivax* and *P. falciparum* were the most commonly detected species, while *P. ovale* and *P. malariae* were detected only in mixed-species infections (qPCR results, Table 1). DNA of *P. knowlesi* was not detected. Only three samples were positive by microscopy, diagnosed as *P. vivax,* thus 92.7 % of *Plasmodium*-infected samples had submicroscopic parasitaemia (microscopy results, Table 1).

**Table 1:** Comparison of screening results for blood samples from community mass blood surveys and passive case detection in the Thai–Myanmar border area

	qPCR (reference)	Expert light microscopy				
	Number of samples	*P. falciparum*	*P. vivax*	*P. malariae*	Mixed *Pf* + *Pv*	Negative
Community mass blood survey						
* P. vivax*	21	–	2	–	–	19
* P. falciparum*	10	–	–	–	–	10
Mixed *Pf* + *Pv*	6	–	1	–	–	5
Mixed *Pf* + *P. ovale*	2	–	–	–	–	2
Mixed *Pf* + *Pv* + *Po*	1	–	–	–	–	1
Mixed *Pf* + *Pv* + *Po* + *P. malariae*	1	–	–	–	–	1
Negative	1306	–	–	–	–	1306
Total *n*	1347	–	3	–	–	1344
Hospital and malaria clinic PCD						
* P. falciparum*	5	5	–	–	–	–
* P. vivax*	4	–	1	–	–	3
* P. malariae*	1	–	–	1	–	–
Mixed *Pf* + *Pv*	22	5	14	–	–	3
Negative	265	–	–	–	–	265
Total *n*	297	10	15	1	–	271

From the blood collected at the hospital/clinic from febrile patients, malaria infections were confirmed in 32 (10.8 %) of 297 samples by qPCR, with the infection rate declining from 19.4 % (13/67) to 11.3 % (6/53) and 7.3 % (13/177) along the March–August–January time line. Most frequently found in these patients were mixed-species infections of *P. falciparum* and *P. vivax*, which accounted for 68.7 % of febrile malaria cases (qPCR results, Table 1). All mixed-species infections (22/22) were misdiagnosed by light microscopy, usually by not detecting the secondary species: of *P.falciparum* diagnoses by microscopy, 50 % contained cryptic *P. vivax* infections; while 93.3 % of *P. vivax* diagnoses contained cryptic *P. falciparum* infections (microscopy results, Table 1). *Plasmodium malariae* was detected in one patient as single infection, but neither *P. ovale* nor *P. knowlesi* were detected by qPCR in these samples.

The age distribution of all samples collected in the community MBS and hospital PCD are shown in Additional file 2. The median age for individuals with *Plasmodium* infections in the Mae Salid Noi community was 14 years, with a mean of 22.8 (17.1–28.5); while the median age of febrile malaria patients in the hospital/clinic was 26.5, with a mean of 27.6 (22.6–32.6). The proportion of qPCR-positive samples in the age groups of 11–20, 21–30 and 31–40 years was significantly higher in the hospital/clinic samples than in the community samples, resulting in the higher median age for malaria patients (Additional file 3).

### Plasma antibody reactivity to *P. falciparum* and *P. vivax* proteins on the microarray

**Fig. 1:**
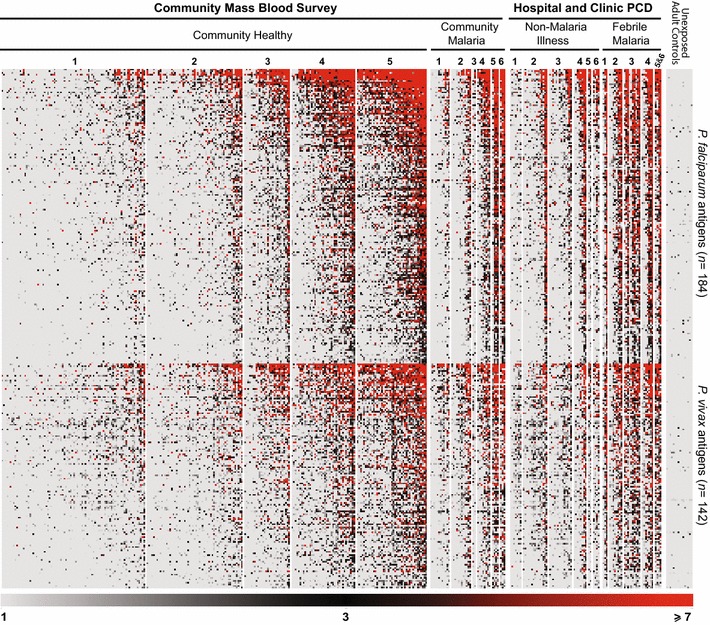
Heatmap of Z-scores of intensity of antibody binding to *P. falciparum* and *P. vivax* proteins on the microarray, segregated by decade of age. Samples were collected from the community in Mae Salid Noi (*n* = 298) and from febrile patients of the hospital/malaria clinic in Mae Tan (*n* = 83). Healthy community and non-malaria illness samples were qPCR-negative for *Plasmodium* sp., community malaria and febrile malaria samples were qPCR-positive for *Plasmodium* sp.. The decade of age of sample donors is shown at the top of heatmap signals, and correspond to years of age as: 1 (up to 10 years old), 2 (11–20), 3 (21–30), 4 (31–40), 5 (41–50) and 6 (51–60+). The youngest age available from community samples was 3 years old, and from PCD, 5 years old. The colored gradient indicates the number of standard deviations above the mean signal intensity of unexposed adult controls from the USA (Z-score). Individual samples appear as *columns*, seroreactive proteins appear as *rows*

A subset of plasma samples from the community mass blood surveys and hospital/clinic PCD were probed on a protein microarray, resulting in 184 *P. falciparum* and 142 *P. vivax* proteins being recognized as immunogenic by antibodies. A heatmap shows the antibody reactivities to these proteins by the individual samples, segregated by collection site, malaria infection status and age (Fig. 1); Additional file 4 presents the gene ID, names and description of all 326 immunogenic proteins, along with their seroprevalence rates for the different age groups in the community and PCD samples.

Many well-known antigens, such as several *P. falciparum* merozoite surface proteins (MSP), apical membrane antigen 1 (AMA1), liver stage antigen 1 (LSA1), serine repeat antigens (SERA) 3, 4 and 5; erythrocyte membrane protein 1 (PfEMP1); and *P. vivax* circumsporozoite protein (CSP), early transcribed membrane proteins (ETRAMP), multiple MSP and SERA proteins, amongst many others were recognized, and detailed seroprevalence rates for these antigens are presented in Additional file 4. The top ten most immunogenic proteins of *P. falciparum* and *P. vivax* for the entire Thai cohort are presented in Table 2.

**Table 2:** Top ten most immunogenic proteins of *P. falciparum* and *P. vivax* for plasma samples from the Thai–Myanmar border area

	Description	% Seropositive (*n* = 381)
*P. falciparum gene ID and fragment*		
PF3D7_0220000, exon 2 segment 1	Liver stage antigen 3 (LSA3)	76.4
PF3D7_1300300, CIDR1	Erythrocyte membrane protein 1, PfEMP1	66.9
PF3D7_0930300, segment 2	Merozoite surface protein 1 (MSP1)	66.9
PF3D7_0930300, segment 1	Merozoite surface protein 1 (MSP1)	66.7
PF3D7_1203700, exon 3	Nucleosome assembly protein (NAPL)	66.1
PF3D7_1335100	Merozoite surface protein 7 (MSP7)	63.3
PF3D7_0202000, exon 2 segment 1	Knob-associated histidine-rich protein (KAHRP)	62.2
PF3D7_1014100, segment 1	Conserved *Plasmodium* protein	61.9
PF3D7_0220000, exon 2 segment 2	Liver stage antigen 3 (LSA3)	61.4
PF3D7_1038400, exon 2 segment 1	Gametocyte-specific protein (Pf111)	60.4
*P. vivax Gene ID and fragment*		
PVX_083560, exon 2 of 2	Hypothetical protein, conserved	77.7
PVX_003770, exon 1 of 2	Merozoite surface protein 5 (MSP5)	69.3
PVX_122865, exon 1 of 5	Hypothetical protein, conserved	68.2
PVX_123810, exon 2 of 2 segment 2	Hypothetical protein, conserved	66.9
PVX_097720	Merozoite surface protein 3 alpha (MSP3a)	66.4
PVX_098025, exon 1 of 3 segment 2	DNA-directed RNA polymerase I	66.4
PVX_000555, exon 2 of 2	Calcium-dependent protein kinase 4, putative	65.6
PVX_098830, exon 1 of 1	Proteasome activator complex subunit 3	65.6
PVX_122180, exon 1 of 1	U1A small nuclear ribonucleoprotein, putative	65.1
PVX_081550, exon 1 of 1	Hypothetical protein, conserved	63.5

Compared to the background reactivity of unexposed controls, Thai samples had significantly higher responses to the proteins on the microarray (Steel–Dwass test, p < 0.0001) (Additional file 5). Amongst the four Thai sera groups based on collection site and malaria status, the intensity and breadth of response was highest in febrile malaria patients, followed by community malaria samples, non-malaria illness patients and healthy community samples (Additional file 5). All plasma samples were reactive to *Plasmodium* proteins on the array, with the number of antigens recognized by individual samples ranging from 11 to 324, median 124.

### Differential antibody acquisition rates for *P. falciparum* and *P. vivax* antigens

**Fig. 2:**
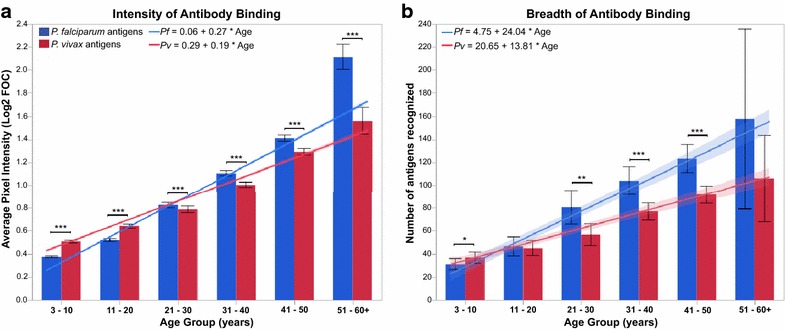
Age-dependent increase in intensity (**a**) and breadth (**b**) of antibody binding to *Plasmodium* antigens by 298 plasma samples collected during community MBS in Mae Salid Noi. The average of responses to 184 *P. falciparum* antigens are shown in *blue*, responses to 142 *P. vivax* antigens are shown in *red*. *Top of bars* represent the mean value, *error bars* represent 95 % confidence interval of the mean. Wilcoxon p values for comparison of responses to the two *Plasmodium* species are shown as *asterisks*: *<0.05, **≤0.01, ***<0.001; p values greater than 0.05 are not shown. Linear regression lines show 95 % CI as *shaded area outside line*, the equation for each regression is shown

For the samples from the community MBS (*n* = 298), children in the youngest age group had the lowest magnitude and breadth of antibody responses, while adults over 50 had the highest; the averaged antibody responses to the 326 *Plasmodium* proteins increased significantly (Steel–Dwass test, p < 0.0001) in intensity and breadth (with one exception) with the increase of age, as shown in Additional file 6. However, the rate by which antibodies against *P. falciparum* and *P. vivax* were acquired differed between the two species, as shown in Fig. 2.

Up to 20 years of age, the intensity of response (Fig. 2a) was significantly stronger against *P. vivax* antigens (Wilcoxon p < 0.0001); but by the age of 21–30 a switch in the dominant target species is observed, with antibodies against *P. falciparum* exceeding those against *P. vivax,* a trend that increased with age. As breadth is partially defined by intensity of the response to individual antigens, a similar trend was observed for the number of antigens recognized by the different age groups (Fig. 2b). In the youngest group, the repertoire of antibodies was larger against *P. vivax* (p = 0.04), but this difference began to disappear in samples from donors in their second decade of life; from age 21 to over 60, samples showed a broadening of their antibody repertoires that recognized significantly more *P. falciparum* antigens than *P. vivax.*

Although the higher intercept values in regression equations of *P. vivax* indicated that in the youngest age groups the antibody response started higher against *P. vivax*, the linear regression lines in Fig. 2a and b showed significantly steeper slopes for the antibody response to *P. falciparum* (slope comparison T-test, p < 0.0001), indicating that the rate of accumulation of antibodies against *P. falciparum* is faster than against *P. vivax* in this population.

Similarly, calculated annual seroconversion (SCR) and seroreversion (SRR) rates (Additional file 4) indicated significant differences in the development of antibody responses against *P. falciparum* and *P. vivax* antigens. SCR values for *P. vivax* antigens (mean 0.048, CI 0.035–0.062) were significantly higher than for *P. falciparum* antigens (0.029, 0.026–0.033) (Wilcoxon p = 0.03), indicating that seropositive rates to *P. vivax* tended to start higher at the youngest age and continued relatively high throughout life. Lower SCR values for *P. falciparum* antigens indicate a low seroprevalence in the youngest groups, but steady acquisition of seropositive states with aging. Loss of antibodies, as determined by the SRR, was higher for vivax antigens (0.022, 0.009–0.035) than for falciparum (0.002, −0.001–0.005) (p < 0.01), indicating that antibodies generated against *P. falciparum* were significantly more stable, while antibodies to *P. vivax* were less stable and were lost at a faster rate than those against *P. falciparum*.

### Temporal changes in antibody responses to *Plasmodium* in the community

Temporal changes in antibody responses were investigated in children (<15 years old) and adults (>15 years old) from the village using longitudinal samples collected from the same individuals at three time-points, 5 months apart. For confirmed *Plasmodium* cases, individuals had qPCR-positive samples in March 2013 followed by two qPCR-negative samples, in August 2013 and January 2014; age- and gender-matched controls had three consecutive qPCR-negative samples at these time points.

**Fig. 3:**
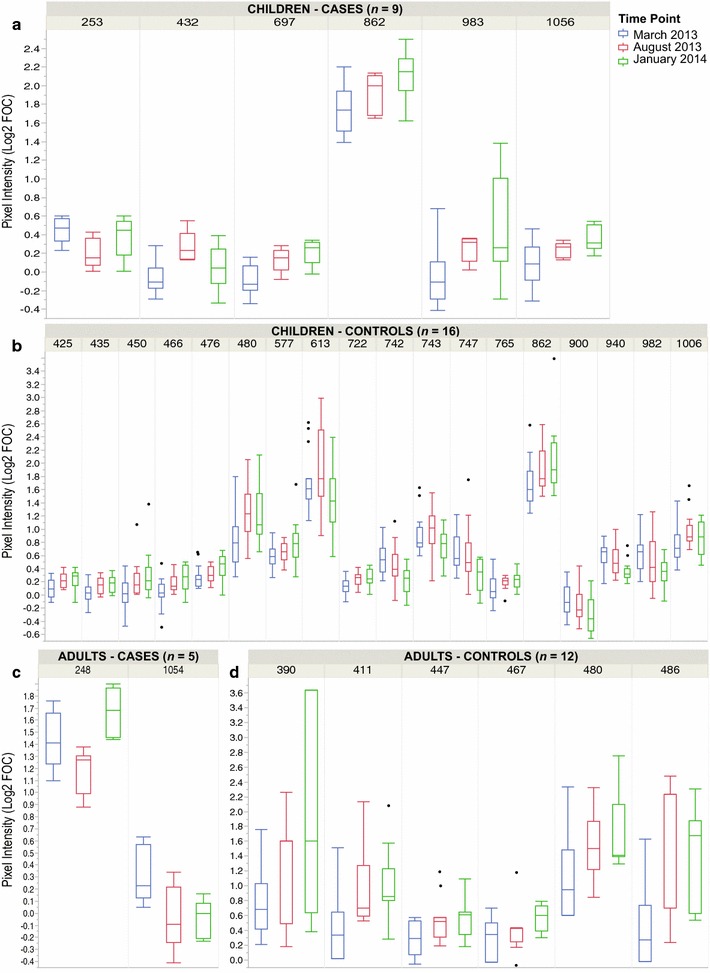
Antibody responses to antigens with significant seasonal changes. The intensity of antibody responses of children and adults with confirmed malaria in March 2013 (cases, *panels* **a**, **c**), and matched uninfected controls (**b**, **d**) are shown for antigens with at least one significant change (p < 0.05) in antibody levels between two time points. Antigens are identified in the figures by their microarray index number, and their corresponding gene ID and protein description are found in Additional file 4. The* box* indicates the first and third quartiles, the *line inside the box* the median pixel intensity value. The *whiskers* represent the minimum and maximum value

For each of the 326 antigens examined for changes in antibody binding along the 10 months studied, only the 32 shown in Fig. 3 had significant (p < 0.05) fluctuations at one or more time points. Children showed the most temporal changes in their antibody responses, regardless of infection status, usually as significant boosting of their responses by August 2013 that sometimes dropped or continued to rise at the last time point (Fig. 3a, b). Some notable antigens that were boosted in August in the children’s responses were the *P. falciparum* gametocyte-specific protein *Pf*111 (index 432 and 435), SERA 3 (index 450) and MSP7 (index 466). *Plasmodium falciparum* erythrocyte membrane protein 1 and SERA 4 (index 613 and 982, respectively) showed a significant decay between the time points before and after the rainy season (March 2013 and January 2014). Adults had more stable responses and fewer antigens varied significantly along the seasons (Fig. 3c and d), but their responses usually were boosted to peak in January 2014.

The overall trend for antibody responses to *Plasmodium* antigens in these samples was a rise from the March 2013 levels that peaked in August 2013 for controls and January 2014 for cases, in both children and adults (Additional file 7). This is coincident with before, during and after the long rainy season (May to October), which is associated with low, peak and decline of parasite transmission season in the region. Although controls were qPCR-negative at the three sample collection times, the possibility of asymptomatic *Plasmodium* infections in the intervening months cannot be ruled out, and could result in the boosting of their antibody responses, as seen.

### Comparison of antibody responses between asymptomatic and febrile malaria cases

Asymptomatic cases were defined by the lack of malaria symptoms up to 5 months after a *Plasmodium* infection confirmed by qPCR and consisted of 33 individuals from the community, with a median age of 14 and 60 % of them below 20 years of age. Febrile malaria cases were defined by the presence of fever and other symptoms at the time of confirmed *Plasmodium* infection, and consisted of 32 hospital/clinic patients, with a median age of 26.5 years.

Mixed *Pf* + *Pv* infections were significantly more frequent amongst febrile patients than amongst asymptomatic cases (OR 12.46 (3.96–39.19), p < 0.0001); while single *P. vivax* infections were significantly more frequent amongst the asymptomatic cases than in febrile malaria cases (OR 7.35 (2.18–24.73), p = 0.001). Single infections with *P. falciparum* were found in equal proportions between the two groups (OR 1.74 (0.53–5.73), p = 0.36).

**Fig. 4:**
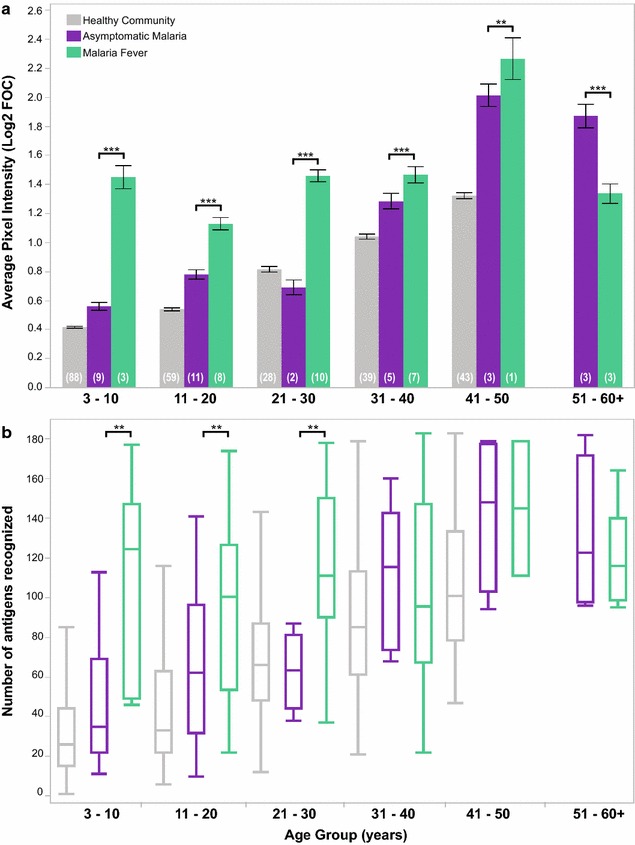
Comparisons of intensity (**a**) and breadth (**b**) of antibody responses to *Plasmodium* antigens by plasma samples from asymptomatic (*purple*) and febrile (*green*) malaria cases, segregated by age group. Healthy community samples (qPCR-negative for *Plasmodium*) are shown in *grey*. The number of samples (*n*) in each age group appears in parenthesis. *Top of bars* represent the mean pixel intensity value of antibody binding to 326 antigens, *error bars* represent 95 % confidence interval of the mean. For *box whisker* plots, the *box* indicates the first and third quartiles, the *line inside the box* the median. The *whiskers* represent the minimum and maximum value. Wilcoxon test p values comparing the means between the asymptomatic and febrile cases within each age group are shown as *asterisks*: **<0.01, ***<0.001; p values greater than 0.05 are not shown

The intensity and breadth of their antibody responses are shown in Fig. 4. In general, asymptomatic cases showed antibody responses significantly elevated from the healthy community baseline (Wilcoxon test for intensity, p < 0.0001; breadth, p < 0.05); however, it was still significantly lower than in febrile patients up to age 50 (Fig. 4a). Febrile patients also had significantly broader antibody repertoires than asymptomatic cases up to the age of 30 years, but this difference disappeared in older adults (Fig. 4b). The antibody response was also significantly higher (p < 0.0001 for each comparison) in malaria fever cases when samples were grouped by the infecting *Plasmodium* species, i.e., in *P. falciparum*, in *P. vivax* and in mixed *Pf* + *Pv* infections, regardless of age.

In the oldest age group, plasma from febrile malaria patients aged 51 and older showed a drop in intensity of binding significantly below the level of antibody responses by asymptomatic cases. It is only in this group that individual antigens were found to elicit significantly higher antibody responses in asymptomatic carriers than febrile patients. These 21 *P. falciparum* and 7 *P. vivax* antigens and their Wilcoxon p values are presented in Additional file 4. Apart from these antigens, no other serological markers could distinguish individuals protected from symptomatic manifestations of malaria.

## Discussion

In a pilot study in the village of Mae Salid Noi and town of Mae Tan, a high level of submicroscopic infections was found amongst asymptomatic and symptomatic adults [17]. Now, from an age-stratified cohort six-times larger, new observations from this low transmission area are added regarding the frequency of asymptomatic and submicroscopic infections, differences in rates of antibody acquisition to *P. falciparum* and *P. vivax* antigens, and antibodies profiles associated with seasonal changes and with clinical and subclinical malaria.

While the detection limit of expert microscopy is approximately 100 parasites per μL [6], the qPCR technique used here detected as few as 0.05 parasites per μL of whole blood. In the community mass blood surveys, *Plasmodium* infection rates estimated by qPCR were nearly 14-times higher than those estimated by expert microscopy, and despite the overall low parasite prevalence in the population (3.0 %), over 90 % of infections were both asymptomatic and submicroscopic at the time of the survey. This is consistent with the mounting evidence from other low transmission areas in the Amazon, Africa and Southeast Asia, where a previously unrecognized reservoir of *Plasmodium* infection is rising in the form of asymptomatic submicroscopic parasitaemia [8–12, 28–31]. In areas of very low transmission submicroscopic carriers are estimated to be the source of 20–50 % of all human-to-mosquito transmissions [14]. Although the importance of submicroscopic infections for malaria transmission is still unclear [32], the Malaria Eradication Research Agenda suggests that any parasitaemia, no matter how small, may be potentially a source of transmission and thus a threat to malaria elimination efforts [7].

Mixed-species infections were common in both the community survey and passive case surveillance in the hospital and clinic, where 25 % of asymptomatic infections and 68 % of febrile malaria cases were caused by co-infections of primarily *P. falciparum* and *P. vivax.* All mixed-species infections detected by qPCR were misdiagnosed as single-species infections by light microscopy, primarily by not detecting cryptic *P. falciparum* in the blood smears. The inability to detect and treat malaria infections is a serious threat to the health care of individual patients and to malaria control and elimination. Molecular diagnosis is not practical in the field and hospital or clinic settings, therefore training of hospital technicians and improvement of malaria rapid diagnostic tests are urgently needed, particularly in light of the presence of multidrug-resistant parasites in Thailand [33, 34].

Notwithstanding the small number of parasitaemic samples found in the three MBSs of the community by qPCR, serological measures indicated exposure to malaria parasites in all individuals tested. However, the youngest children showed limited responses, reacting to a small number of antigens, a serological indication of the success of malaria control efforts in the area because very low antibody titers are expected to correlate with reduced parasite exposure events. These children are an attractive target group for serosurveillance because detection of antibodies in this group, after drastic reduction or elimination of local transmission, indicates resurgence of malaria in a population [35, 36]. Serology provides a view of present and past parasite exposures, and seroprevalence rates can be used to define malaria endemicity in an area [37, 38].

It could be expected that the low levels of antibodies against *Plasmodium* in the youngest group would render it especially susceptible to malaria infection and disease, and indeed, in this study half of community malaria cases were of persons below 15 years old. However, despite the lack of high titers or broad antibody repertoires against the parasite, the vast majority of the infections were submicroscopic and asymptomatic, and remained so for at least 5 months. This challenges the notion that individuals with little exposure to malaria have limited ability to suppress parasitaemia and would become symptomatic, but is consistent with epidemiological findings from other low transmission areas [8, 14, 39, 40]. In fact, high levels of antibodies against *P. falciparum* in children from Papua New Guinea were not associated with protection from malaria, rather with higher exposure to parasites [41]. The high prevalence of submicroscopic and asymptomatic infections in the 3–15 years old cohort demonstrates that despite the low antibody titers, efficient parasite-controlling immunity developed after few exposures.

Experimental and natural infection studies have shown that complete strain-specific immunity to vivax malaria may be acquired after two or three exposures, but the rate of acquisition of disease protective immunity differs between *P. falciparum* and *P. vivax* (reviewed in [42]). Abundant evidence showed that the number of clinical episodes of vivax malaria decreased earlier in life than that of falciparum malaria, suggesting a faster acquisition of protective immunity against *P. vivax* [42]. In this study, the intensity and breadth of antibody response in the youngest age groups was significantly higher against *P. vivax,* but a reversal in species-specific immunodominance occurred in adults. Two possible reasons can be suggested to explain this age-related difference in immunodominance, which are not necessarily mutually exclusive. First, the shift in species-specific response with increasing age may result from differences in the biology of the falciparum and vivax infections, such as relapses in *P. vivax* and higher parasitaemia in *P. falciparum*. The estimated SCR and SRR showed that antibodies against *P. falciparum* accumulated continuously and decayed at a slow rate throughout life, whereas antibodies against *P. vivax* were quickly acquired but were less stable. Second, the historical relative prevalence of the two species also could play a role in this difference. From the 1970s to the mid-1990s, the prevalence of *P. falciparum* exceeded *P. vivax* in the region [15], a trend that was reverted in the recent past years, resulting in *P. vivax* currently exceeding *P. falciparum* by 2:1. Hence, adults in the cohort would have historically experienced more falciparum infections than the children, who now are comparatively more frequently exposed to *P. vivax.* Additionally, antibody cross-reactivity between *P*. *falciparum* and *P. vivax* could add confounding factors to this analysis, but this could not be addressed in this study due to the co-endemicity of these two species in the region.

As transmission levels drop further, serology is being recognized as a valuable tool to monitor malaria epidemiology [7]. Examination of age-specific seroprevalence profiles can be used to detect transmission hot-spots [43] and detect changes in local transmission [35, 38]. Furthermore, absence of antibodies against *Plasmodium* has been used to show the success of elimination programmes in several countries [44–46]. As the longevity of antibodies against *Plasmodium* varies between antigens [47, 48] and has both short- [49–51] and long-lived [52–54] components, it is imperative to choose antigenic markers with the appropriate kinetics to detect recent parasite exposure, rather than memory responses from past infections.

The kinetics of antibody responses to hundreds of proteins were explored in the longitudinal cohort, but there was little change at the level of individual markers in the 10 months studied. Children showed the most changes across time, indicative of reinforcement of memory responses or acquisition of new antibodies with time, while there were minimal changes in the adults’ responses. This is similar to what was observed in Kenya, where significant increase in breadth and intensity of antibody response after the malaria season was observed in children below 8 years old, but not in adults [55]. Nonetheless, there was significant overall increase in antibody levels from before to after the rainy season in both adults and children, suggesting that some may have had asymptomatic infections in the intervening months between sampling times. Thus, in order to accurately determine antibody kinetics for use in serological surveillance, more frequent sampling accompanied by molecular screening is required, such as in the study recently published by Helb et al. [56].

Another aim of this study was to identify antigenic targets that elicited higher antibody levels in asymptomatic carriers when compared to febrile patients, and that could be suggested as to play a role in protective responses. However, antibody titers were significantly higher in those who experienced clinical symptoms, especially in the younger age groups. Here, it may be once again the case that high levels of antibodies to *Plasmodium* are indicative of higher exposure rather than of protection to malaria [41]. Not only did symptomatic patients have, in the majority of cases, parasite levels that were detectable by microscopy while asymptomatic carriers had subpatent parasitaemia, the majority of symptomatic cases also carried multi-species infections. These factors likely would augment the antibody response in febrile patients, while asymptomatic carriers are capable of controlling parasite levels effectively by some other mechanism. Interestingly, recent studies showed evidence of upregulation of inhibitory receptors eliciting exhaustion-related phenotypes on T cells associated with defective effector function during chronic parasitaemia or regular reinfections with *P. falciparum* [57] and *P. vivax* [58]. The exhaustion of CD4 T cells may be associated with failure to develop a fully differentiated B cell response in these cases, such as in the subpatent asymptomatic infections common in our cohort, and result in lower levels of antibodies [59]. Nonetheless, other studies designed to discover markers of disease protection in malaria in high and low transmission settings have identified several serological correlates of protection [55, 60, 61].

In summary, the findings of the present study have implications for surveillance, control and elimination of malaria in Thailand. In particular, the data suggests children aged 3–15 years old are a sensitive group for serosurveillance monitoring changes in transmission due to malaria interventions. Additionally, in the community 100 % of *P. falciparum* and mixed species infections, and 90 % of *P. vivax* infections went unrecognized and untreated. Amongst the febrile cases, microscopy failed to detect 75 % of *P. vivax* infections; and of the mixed-species infections, all went unrecognized, being misdiagnosed as a single-species infection or completely undetected. Because current surveillance methods focus on case management, malaria transmission cannot be interrupted if asymptomatic infections are infectious to mosquitoes and contribute to transmission [7, 11]. Currently, implementation of molecular screening methods for population-wide surveillance is not feasible in developing countries, and the field applicable serological surveillance tools are deficient because of lack of optimal serological markers with the appropriate kinetics [36]. The mounting evidence of the shortcomings of the current malaria surveillance, detection and treatment methods argues for a major change in malaria control approaches. For example, mass drug administration to largely healthy populations has been controversial and resistance to this approach is considerable [28, 62, 63]. However, there is an urgent need for discussion and exploration of such alternative approaches by the scientific and public health communities.

## Conclusions

The high prevalence of undetected and untreated *Plasmodium* infections in Tak is a challenge to Thailand’s efforts to eliminate malaria by 2030. The shortcomings of current diagnostic and treatment methods argues for a urgent change in malaria control approaches, with mass drug administration, serological and high-throughput detection methods needing to be explored as potential next-generation tools for malaria control and elimination in areas of low-transmission.

## Additional files

10.1186/s12936-016-1393-4 Age-fitted seroprevalence plots for seroconversion and reversion calculations. The average seroprevalence for each age group is plotted for the seroreactive *Plasmodium* proteins, denoted as P1 through P326. Their corresponding gene ID and protein description are found in Additional file 4.
10.1186/s12936-016-1393-4 Age distribution in years of samples collected at community mass blood survey in Mae Salid Noi (blue) and hospital/malaria clinic PCD (orange). The darkened areas represent *Plasmodium-*positive qPCR samples. The box plot above the histogram indicates the first and third quartiles, the line inside the box the median. The 1.5× interquartile range is indicated by the horizontal line bisecting the box.
10.1186/s12936-016-1393-4 Percentage of *Plasmodium-*positive samples amongst all samples collected per age group in years from the community MBS (blue) and hospital/clinic PCD (orange). The number of samples collected in each age group appears inside the corresponding bar. The p-value for Two-Proportion Z-test is shown as asterisks: ** <0.001; p values greater than 0.05 are not shown.
10.1186/s12936-016-1393-4 List of 326 seroreactive *P. falciparum* and *P. vivax* polypeptides recognized by antibodies in plasma from exposed villagers and malaria patients in Tak Province, Thailand; and their respective seroprevalence rates, seroconversion (SCR) and seroreversion (SRR) rates, and Wilcoxon p values for comparison of mean antibody responses in asymptomatic and febrile malaria senior cases.
10.1186/s12936-016-1393-4 Average and 95% confidence interval of intensity and breadth of antibody response to 326 *Plasmodium sp.* proteins by plasma samples from the community and hospital/clinic patients, segregated by malaria status.
10.1186/s12936-016-1393-4 Average and 95% confidence interval of intensity and breadth of antibody responses to 326 *Plasmodium sp.* proteins by 298 plasma samples from the community, segregated by age groups in decades.
10.1186/s12936-016-1393-4 Average temporal changes in intensity of antibody binding to *Plasmodium* antigens in longitudinal samples collected from adults (**top**) and children (**bottom**) from Mae Salid Noi community. **Cases** had confirmed *Plasmodium* infections in March 2013 and negative qPCR at the other two time-points; **controls** were qPCR-negative at all 3 time-points. The line represent the mean pixel intensity value of antibody binding to 326 antigens, error bars represent 95% confidence interval of the mean. Wilcoxon test p-values comparing the means between time-points are shown as asterisks: *** <0.0001; p values greater than 0.05 are not shown.

## Abbreviations

MBS: mass blood survey
PCD: passive case detection
SCR: seroconversion rate
SRR: seroreversion rate
Pf: *Plasmodium falciparum*
Pv: *Plasmodium vivax*
Po: *Plasmodium ovale*
Pm: *Plasmodium malariae*
