# Supplementary figures and images for: Common asymptomatic and submicroscopic malaria infections in Western Thailand revealed in longitudinal molecular and serological studies: a challenge to malaria elimination
Source: Malar J. 2016 Jun 22;15:333. doi: 10.1186/s12936-016-1393-4 (PMC4918199; doi:10.1186/s12936-016-1393-4)

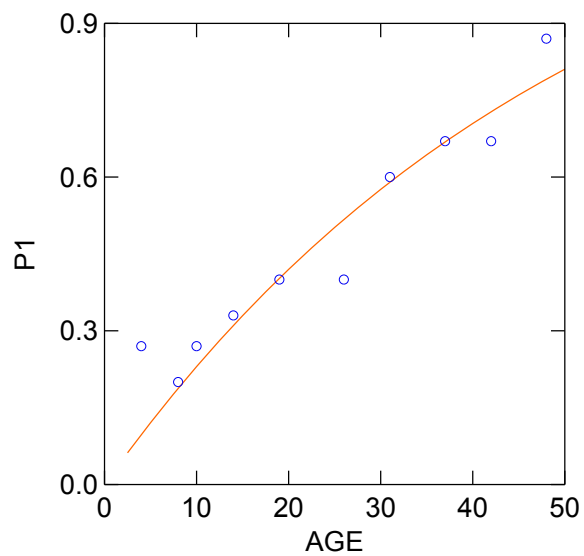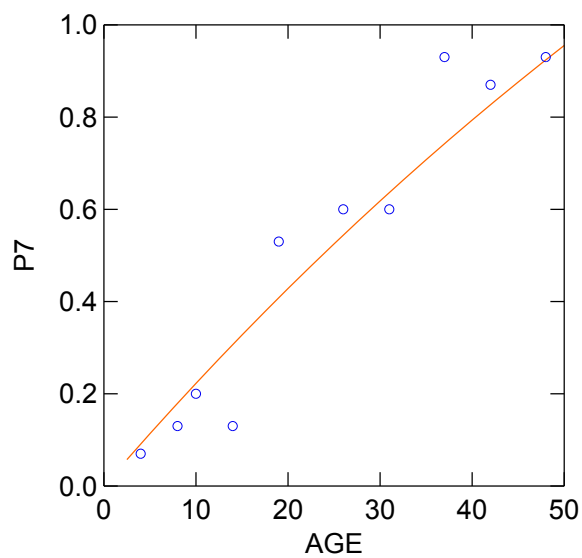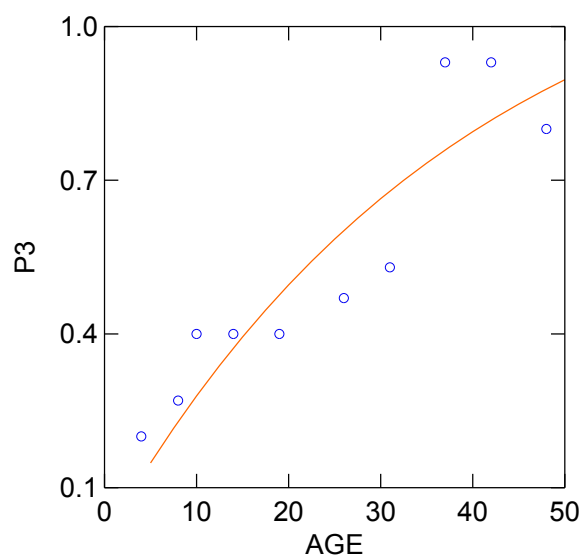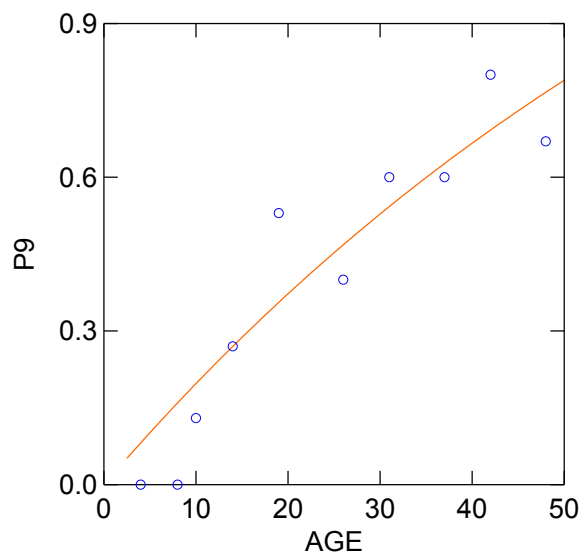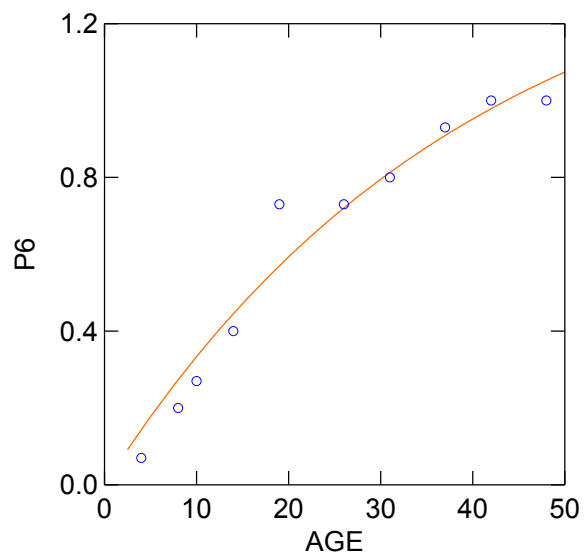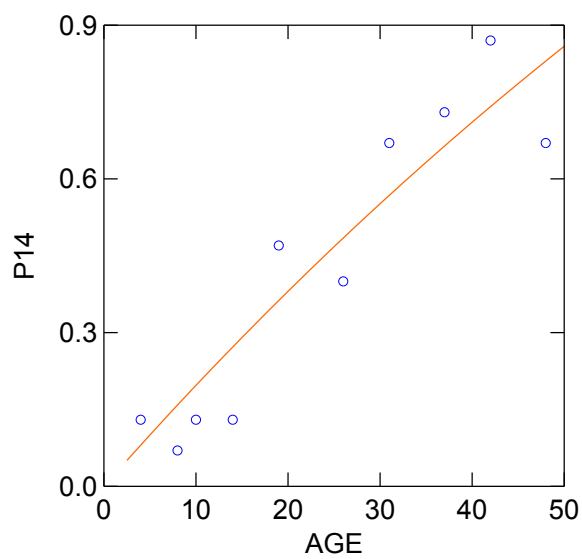

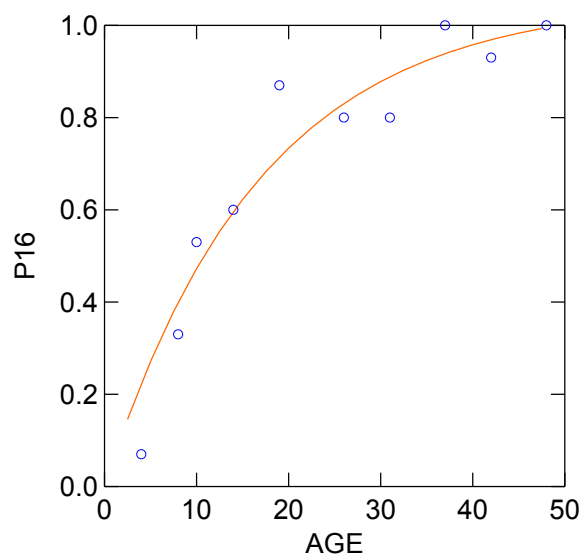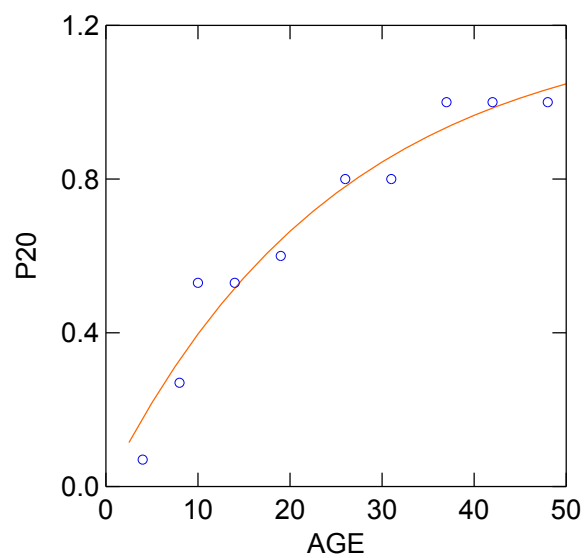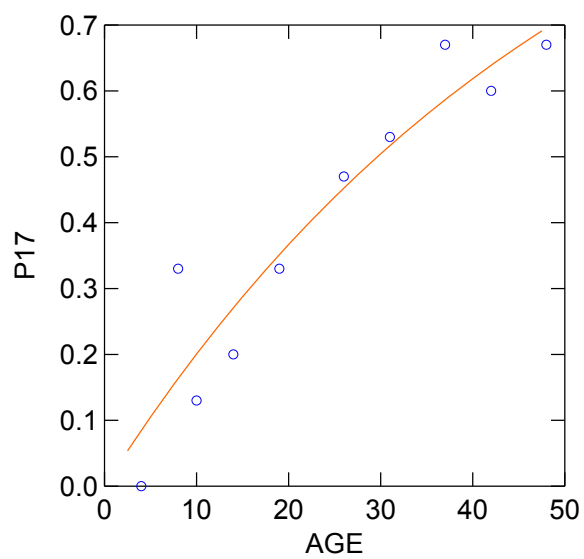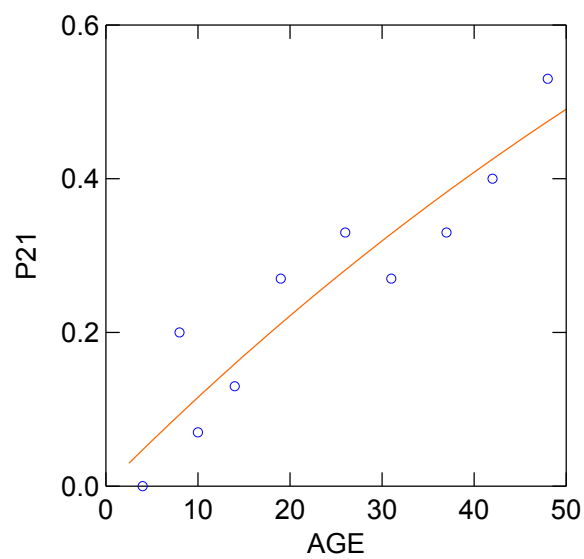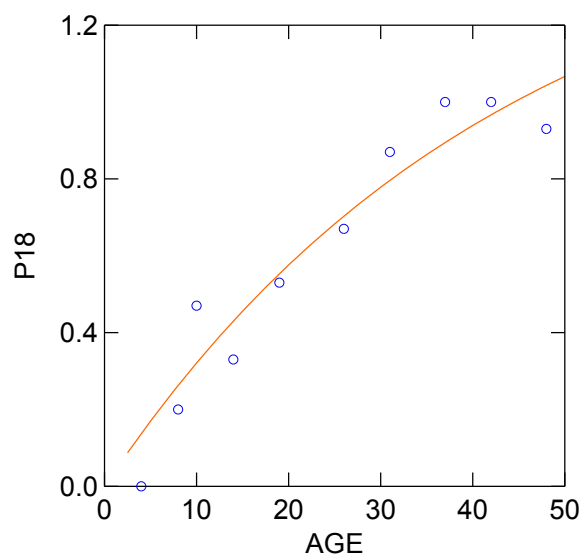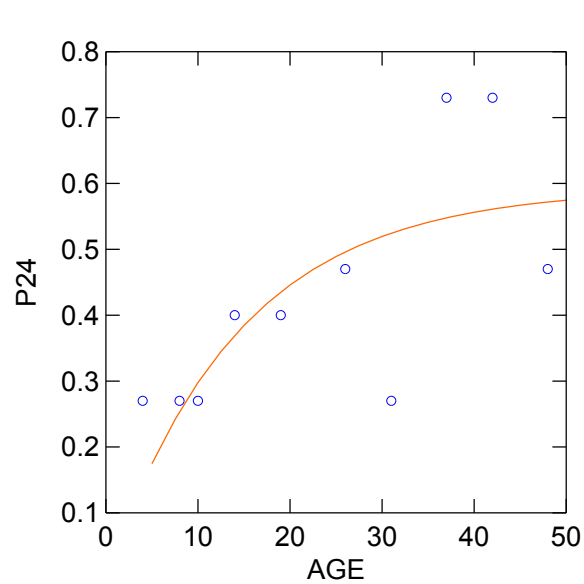

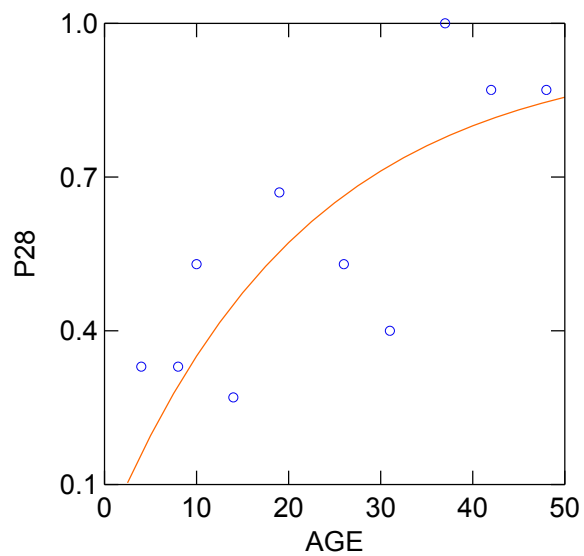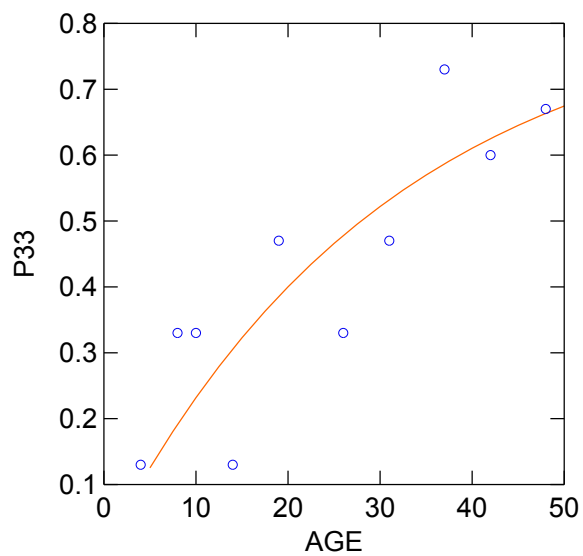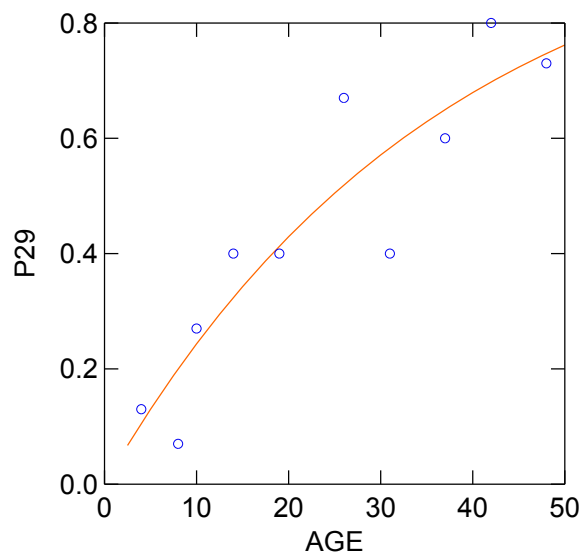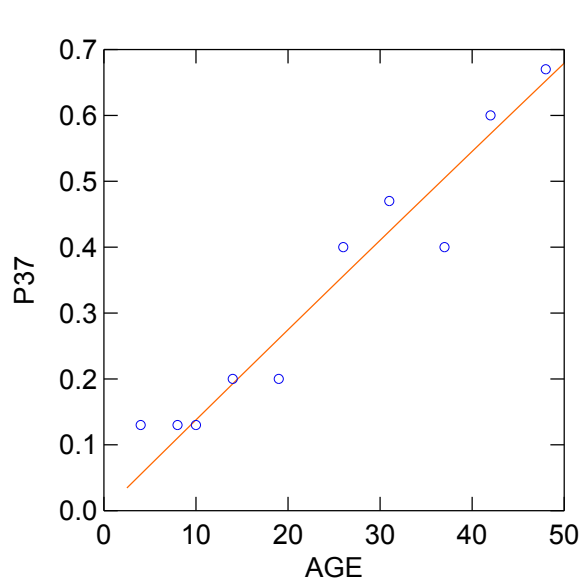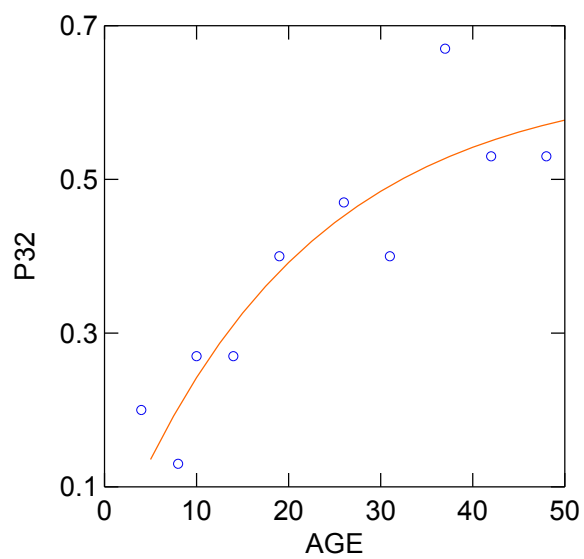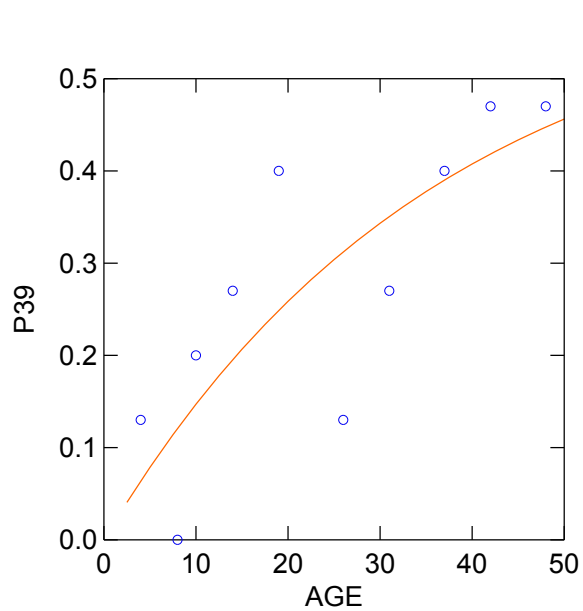

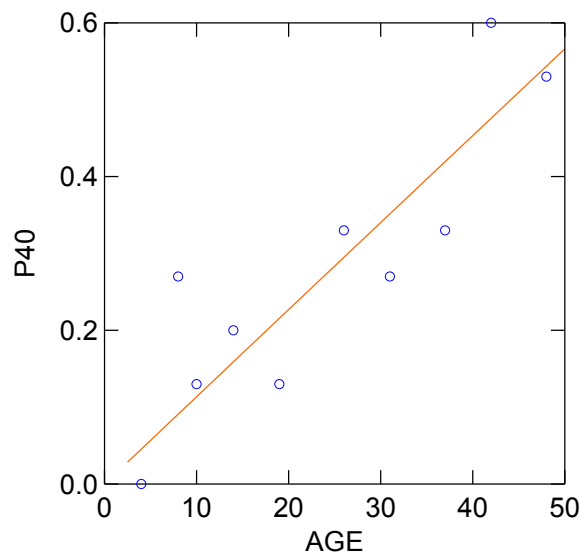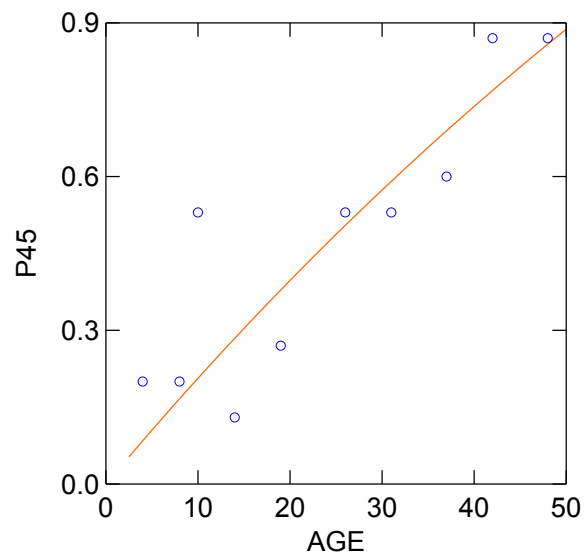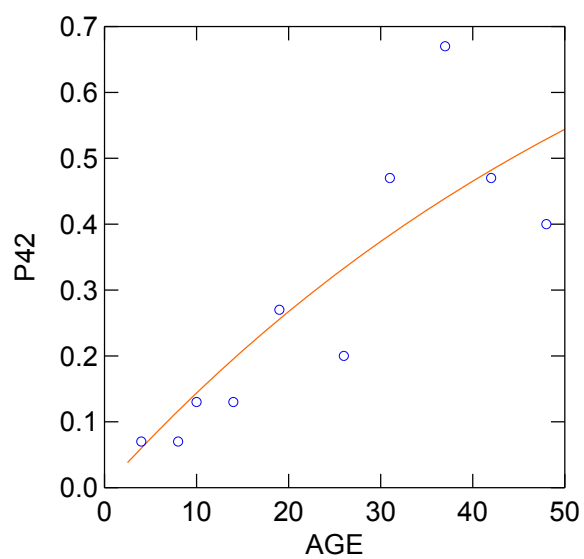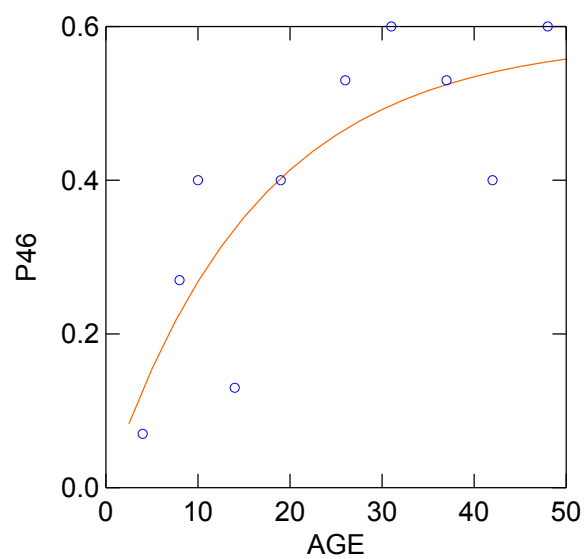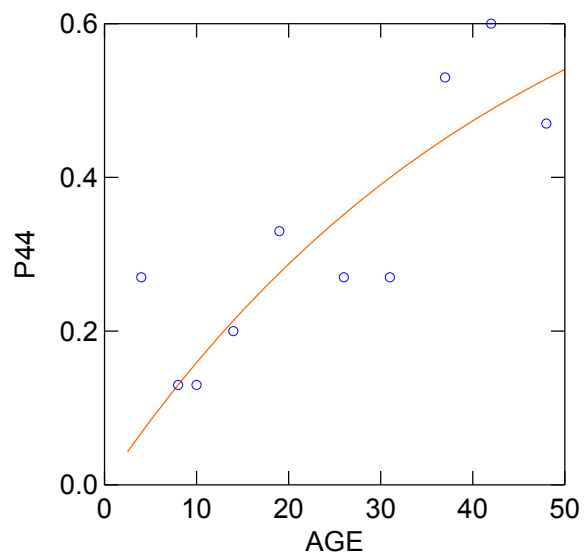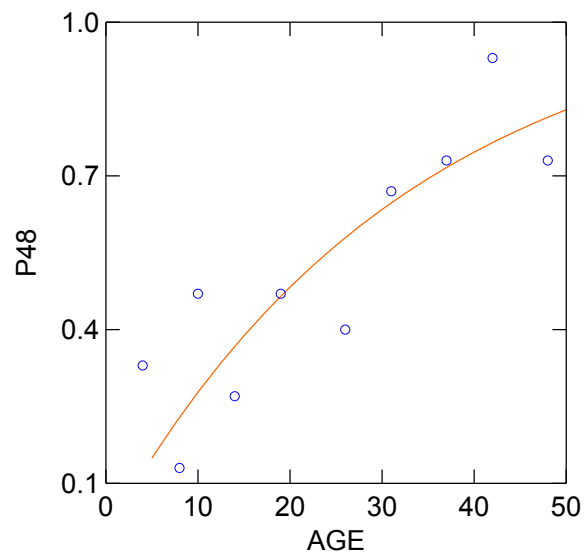

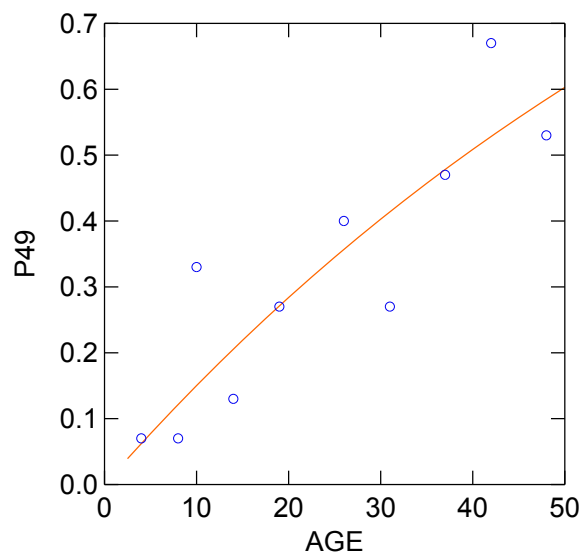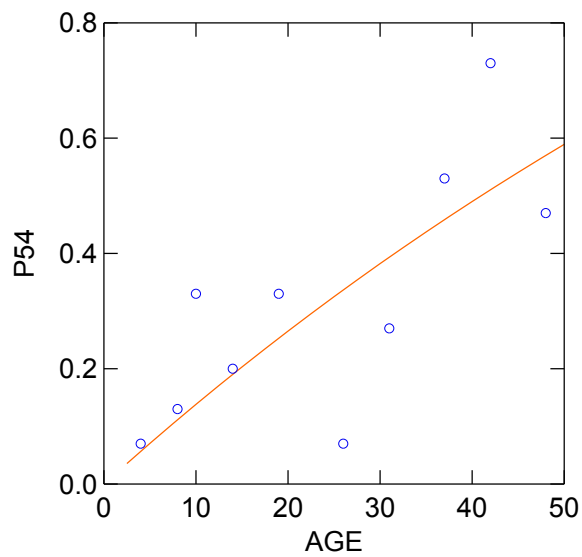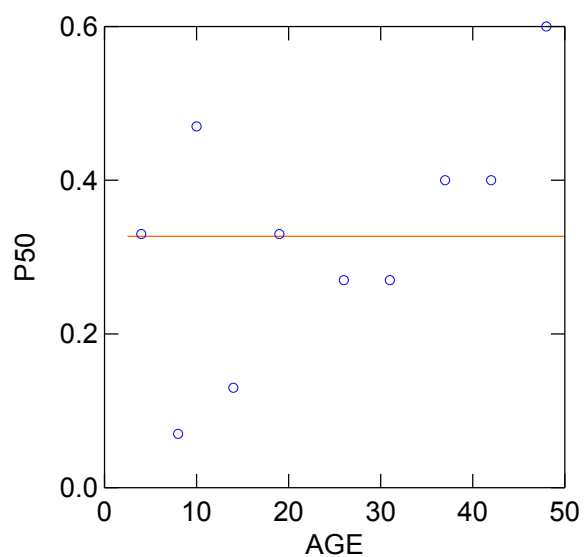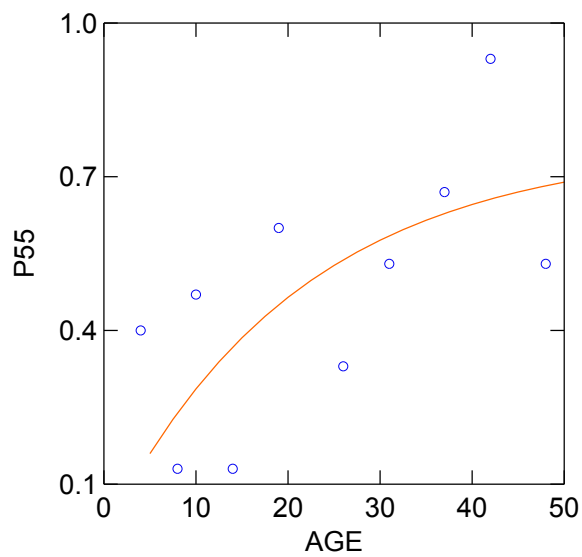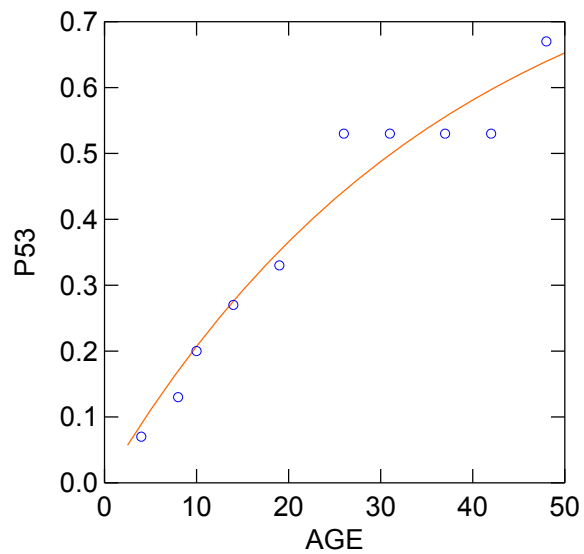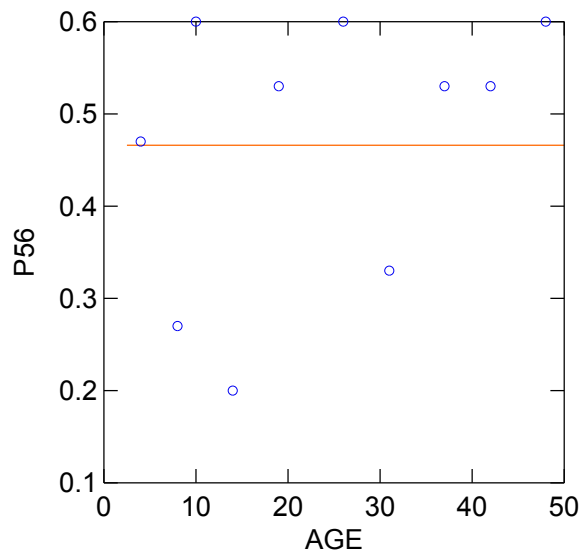

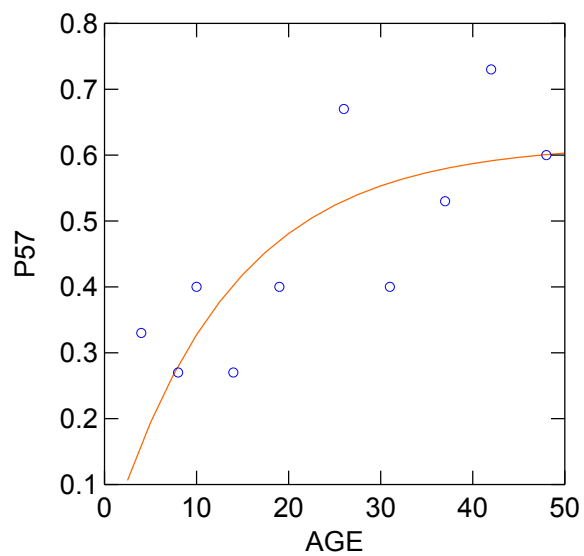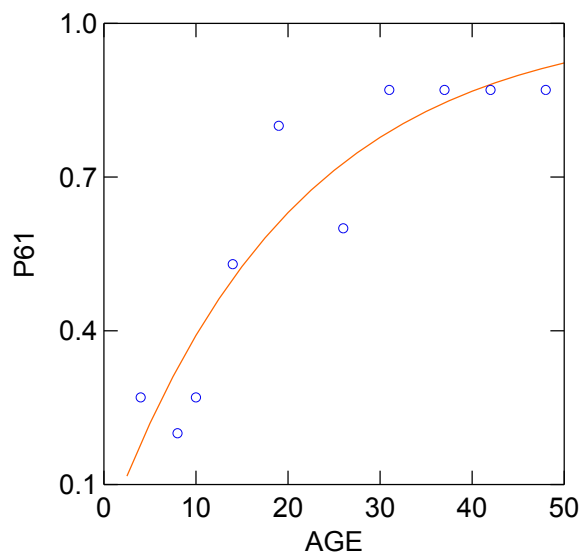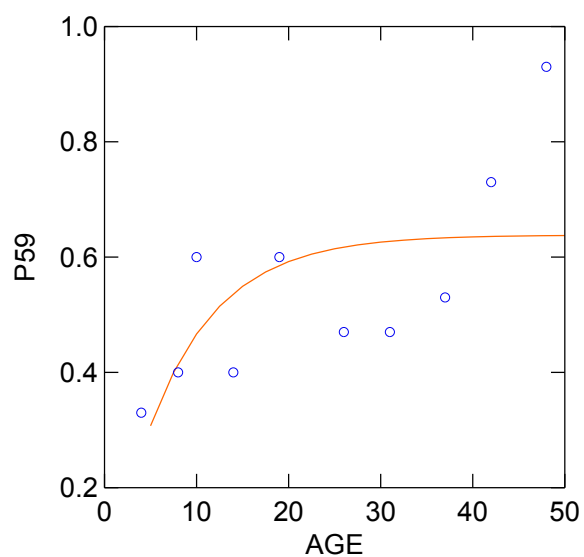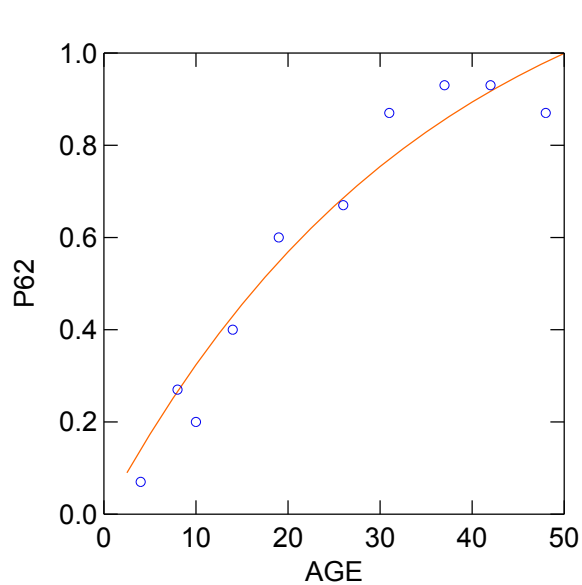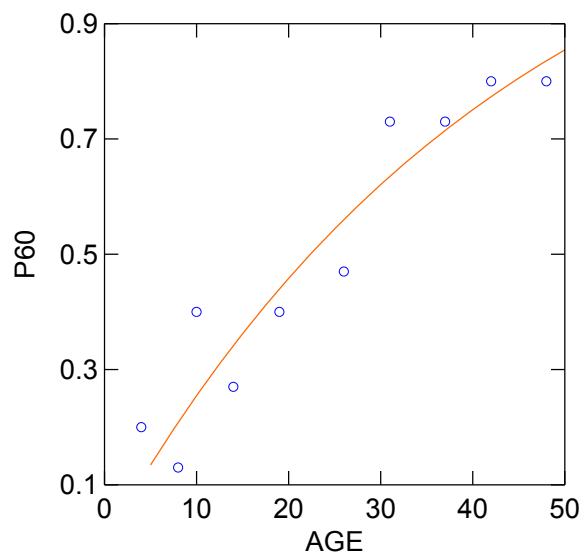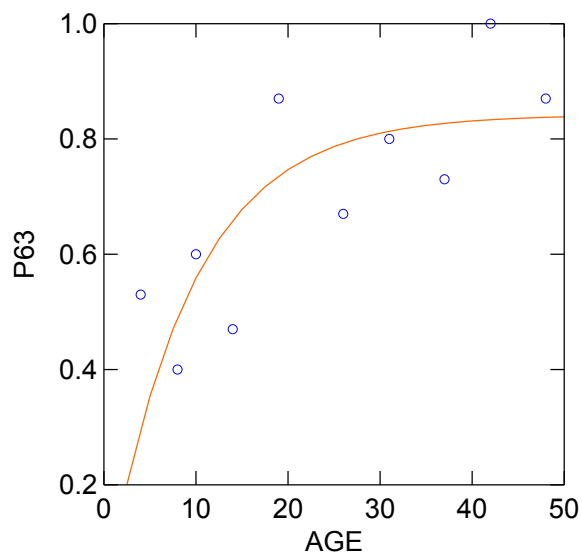

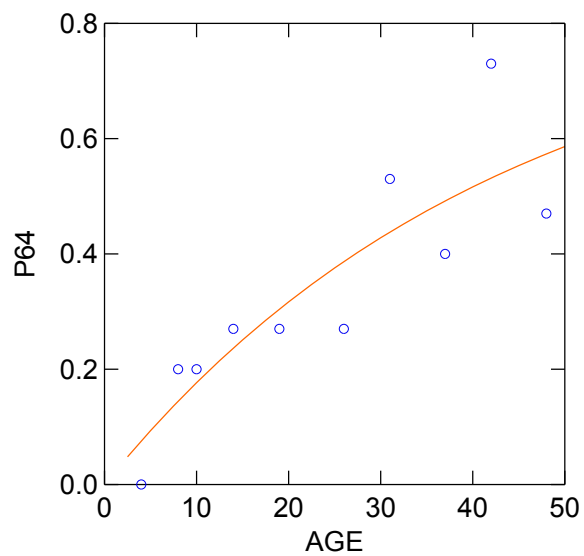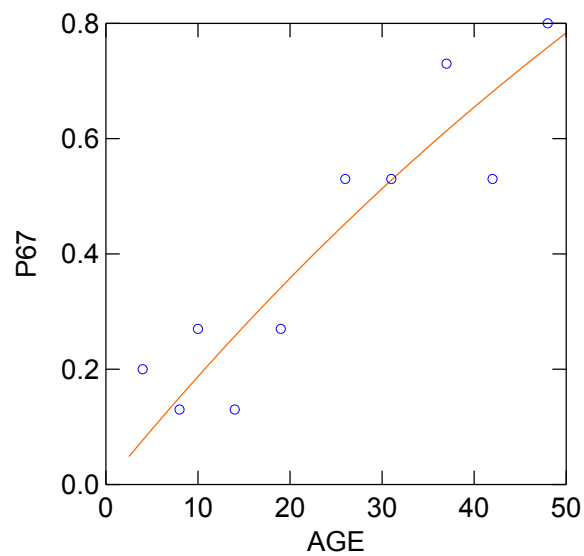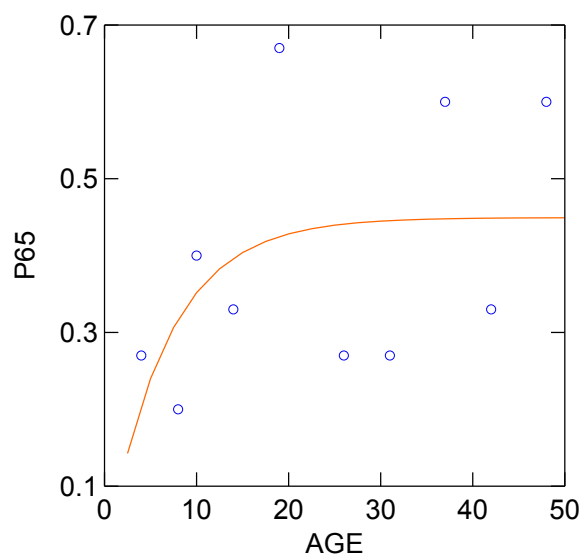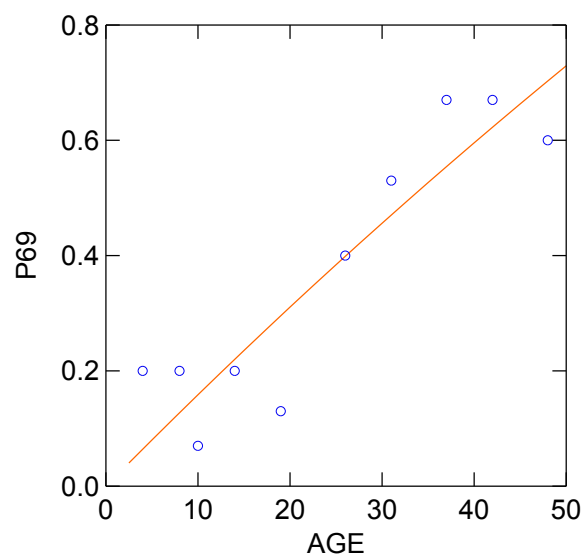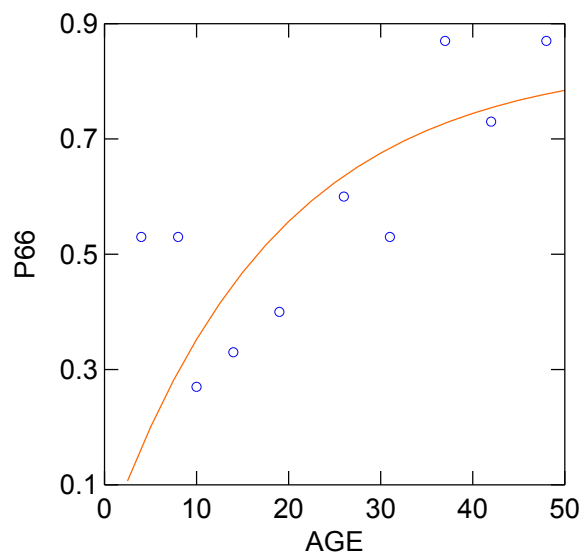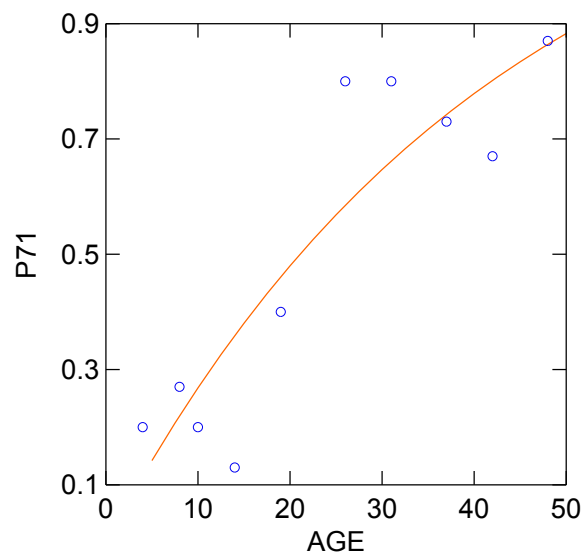

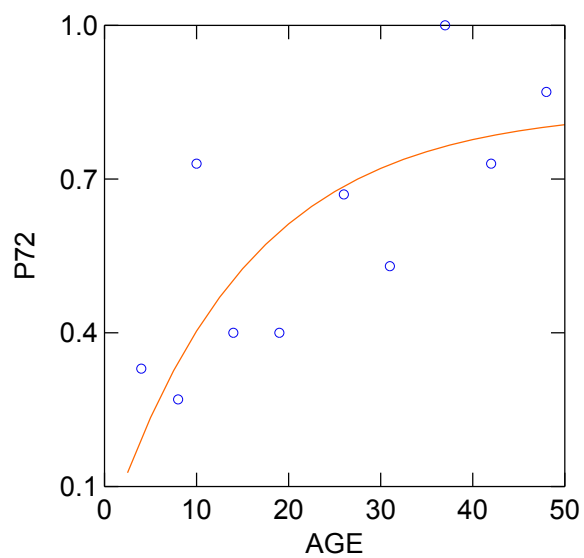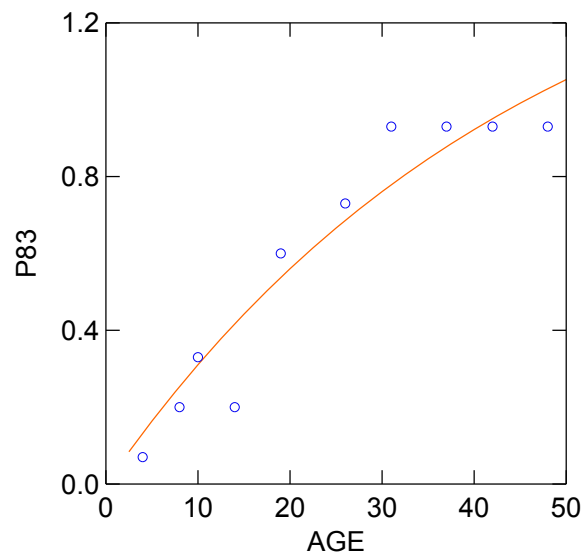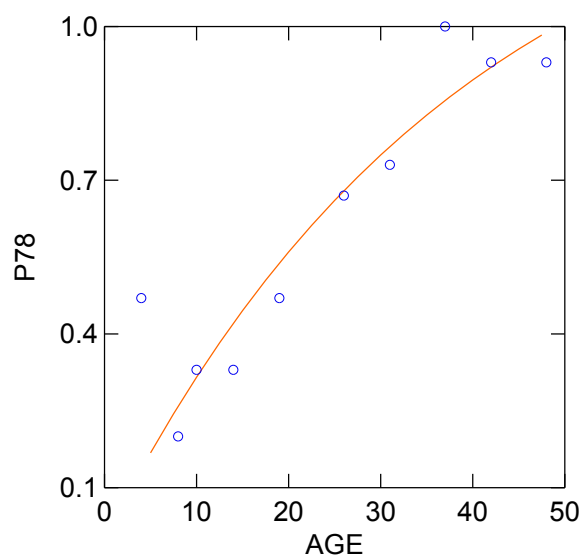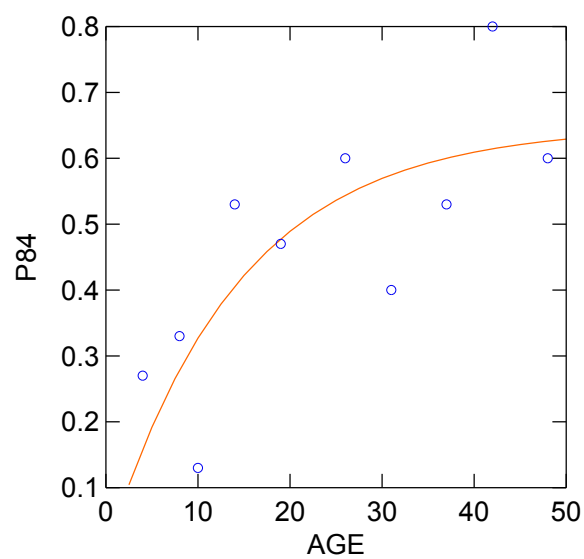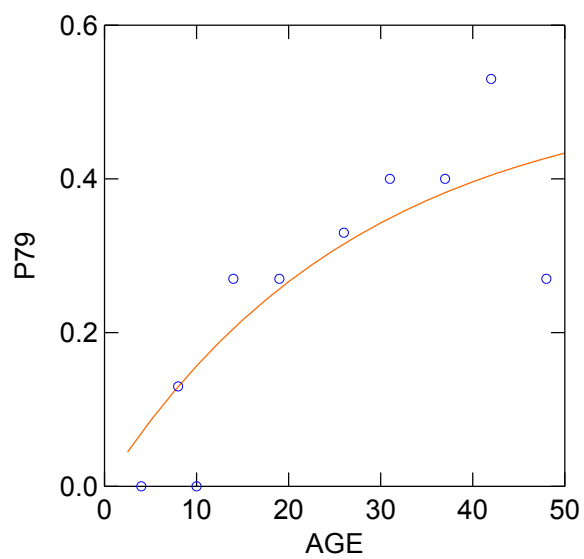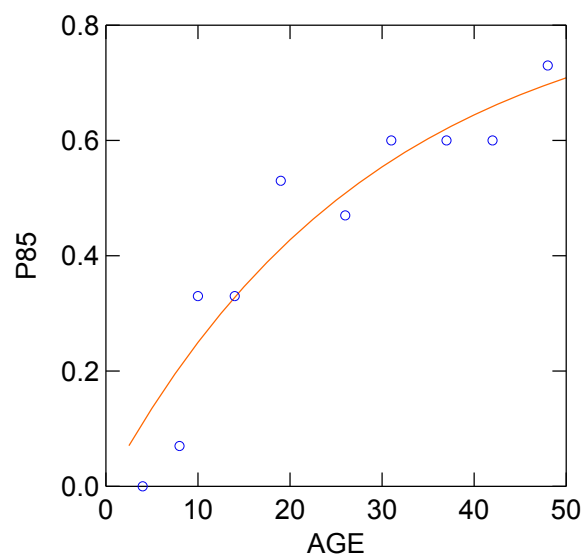

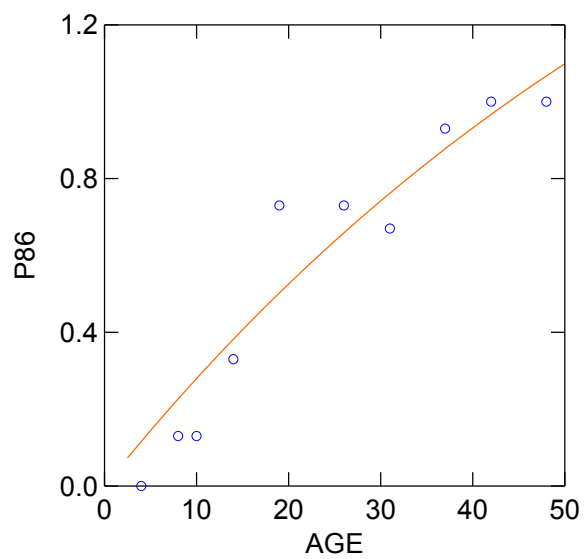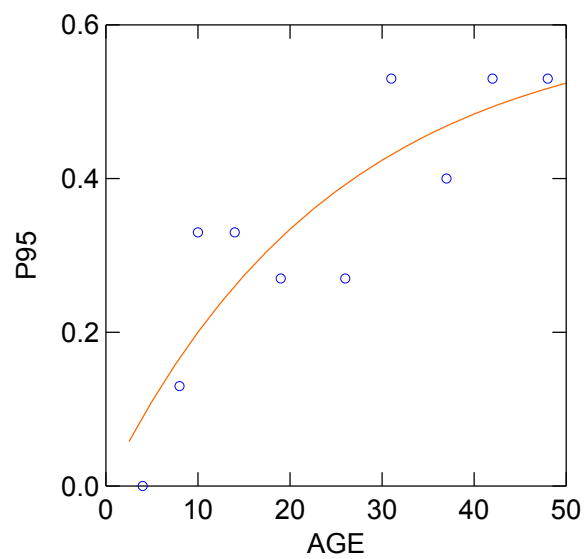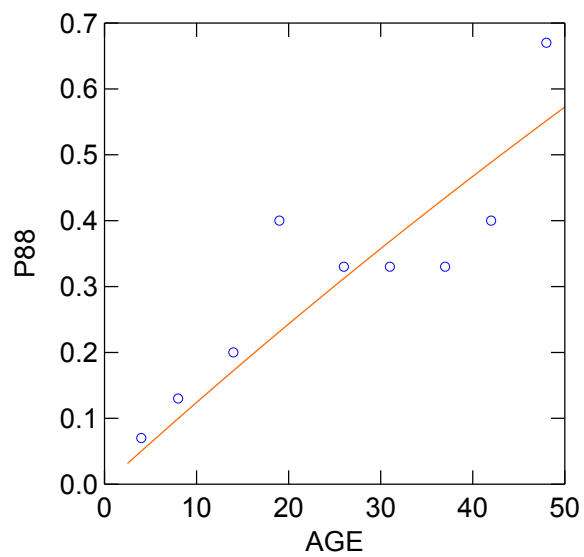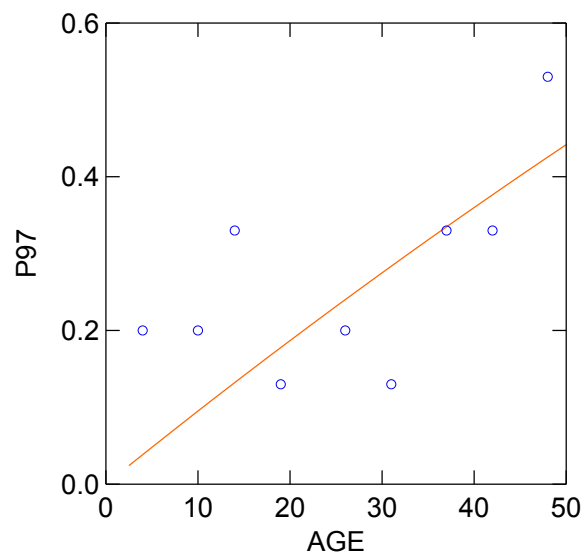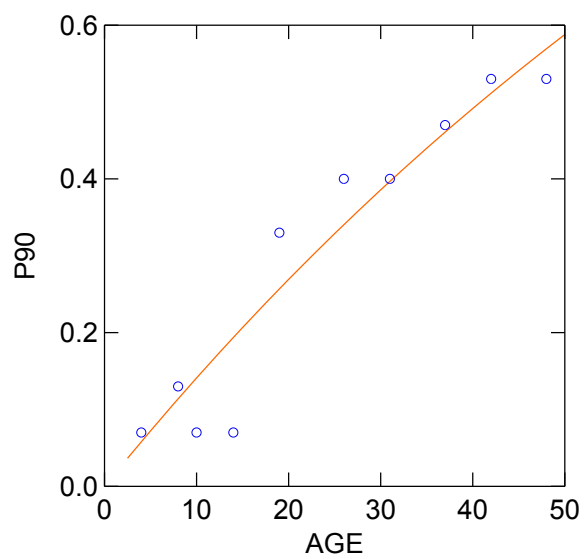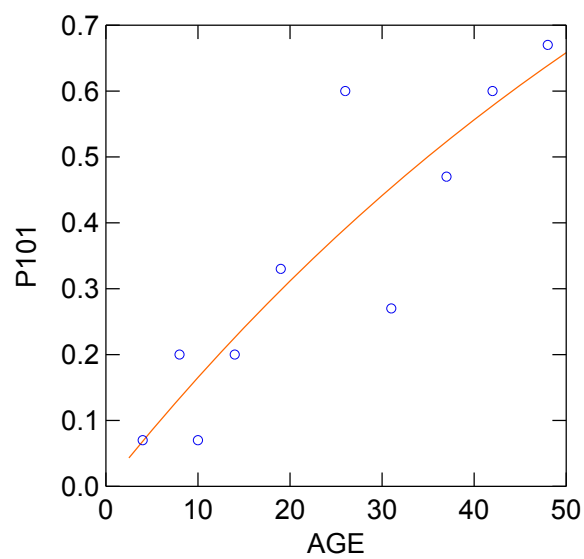

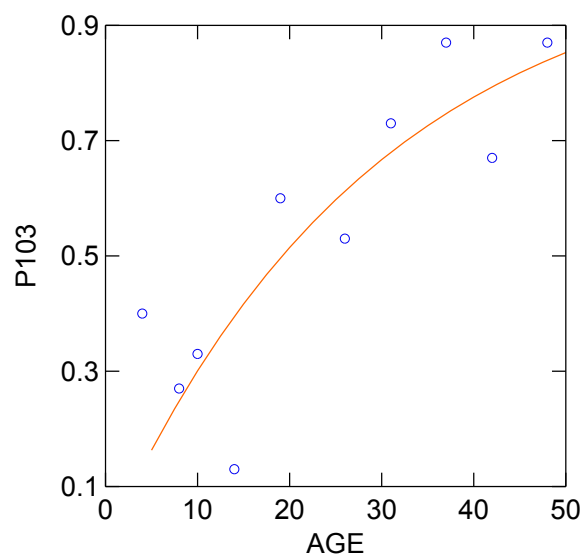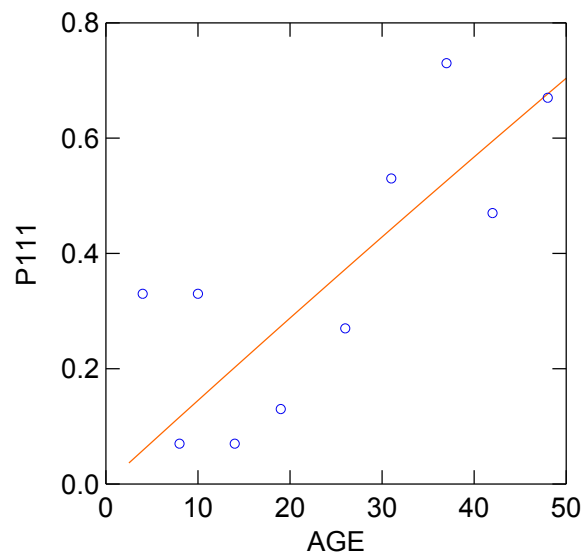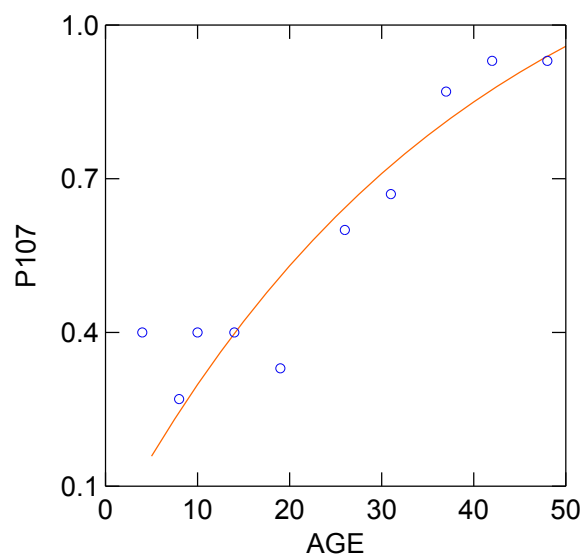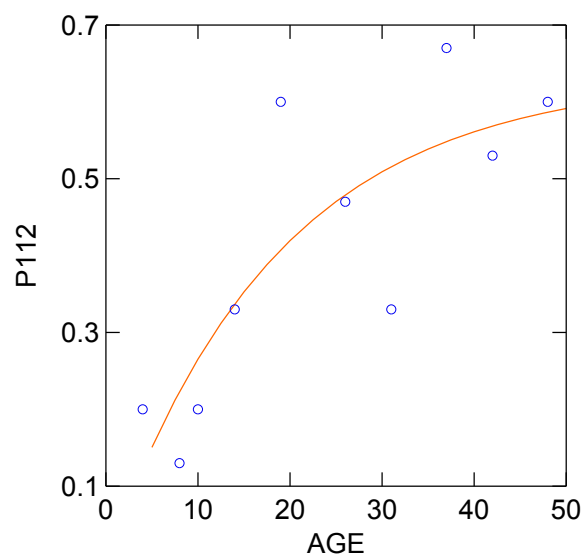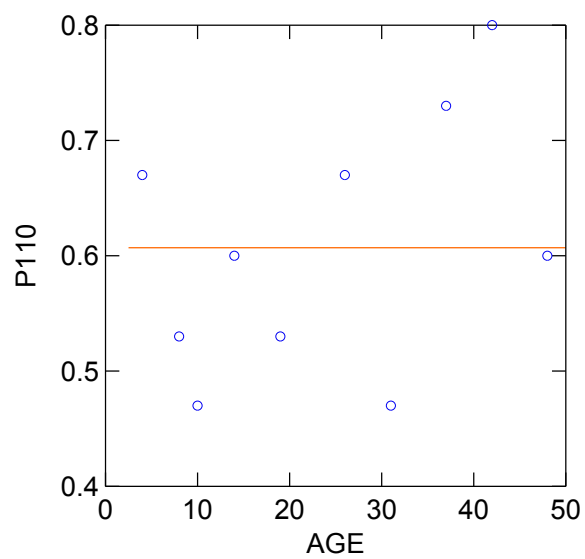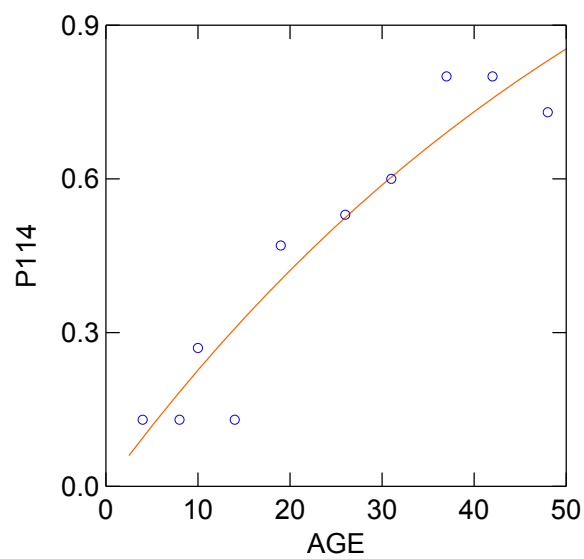

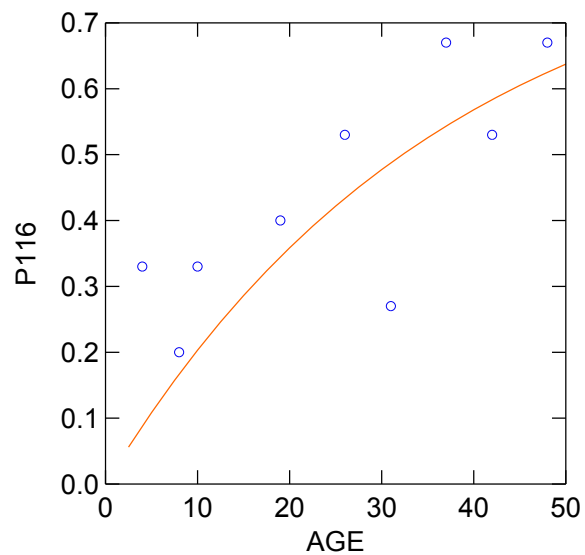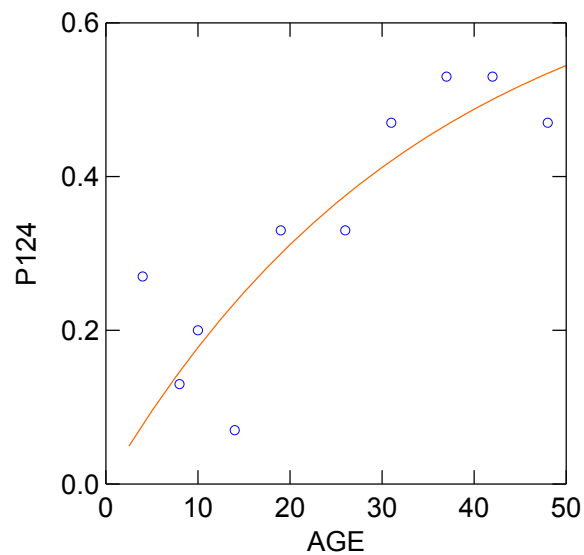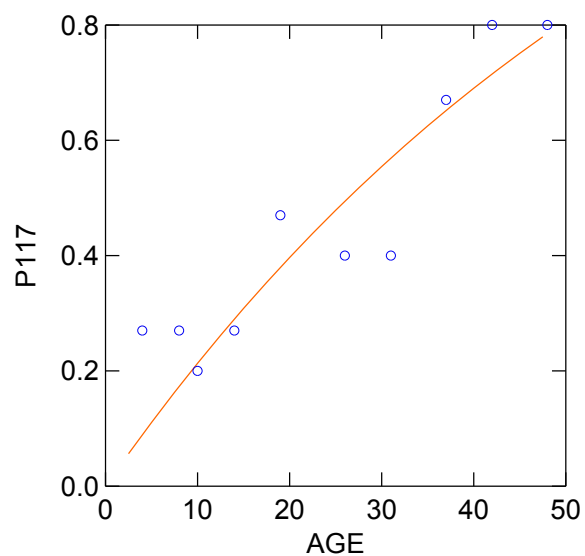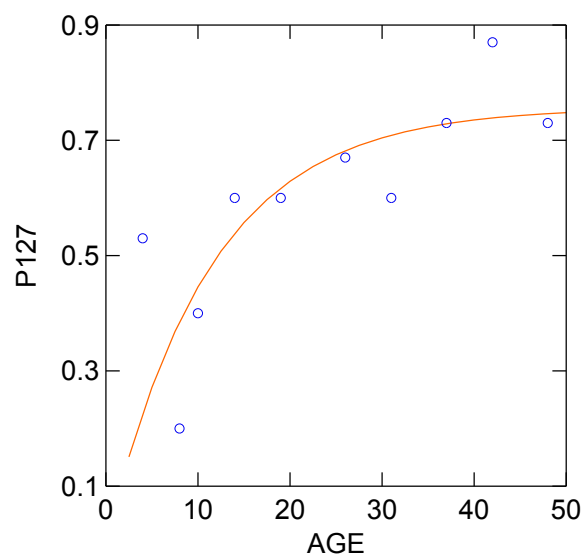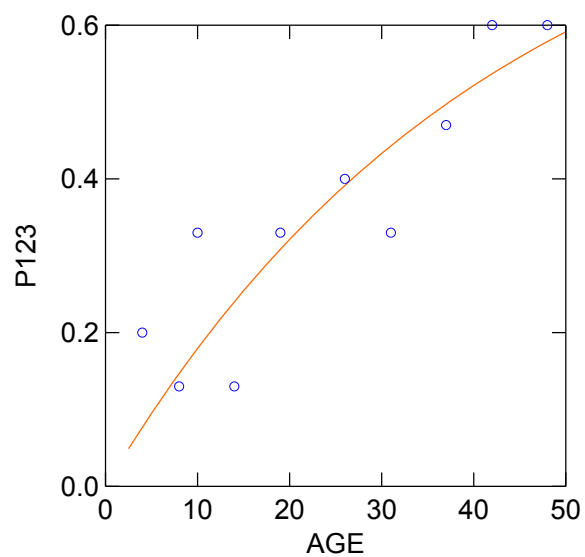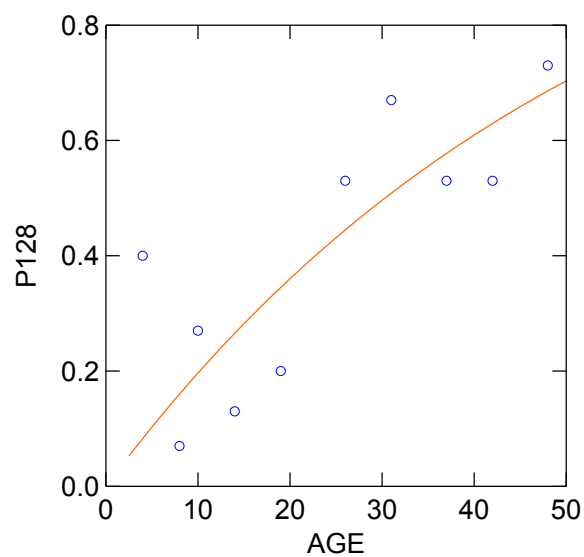

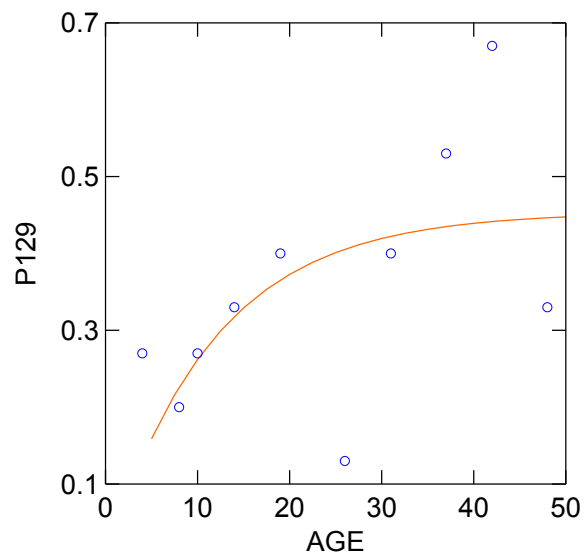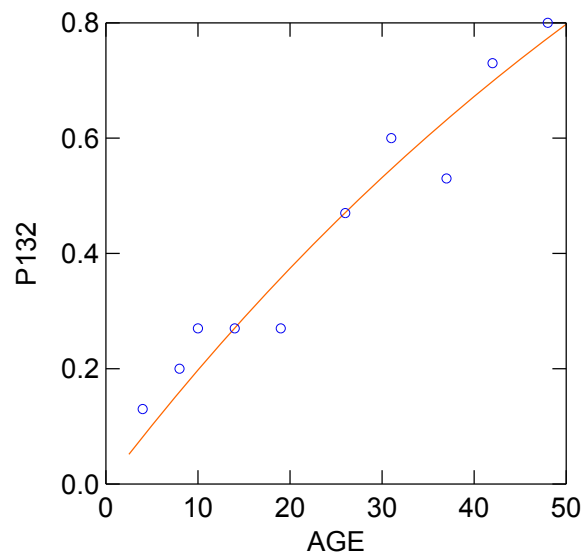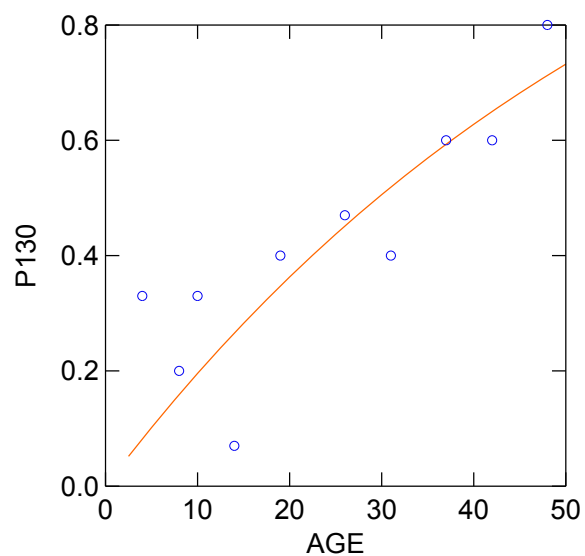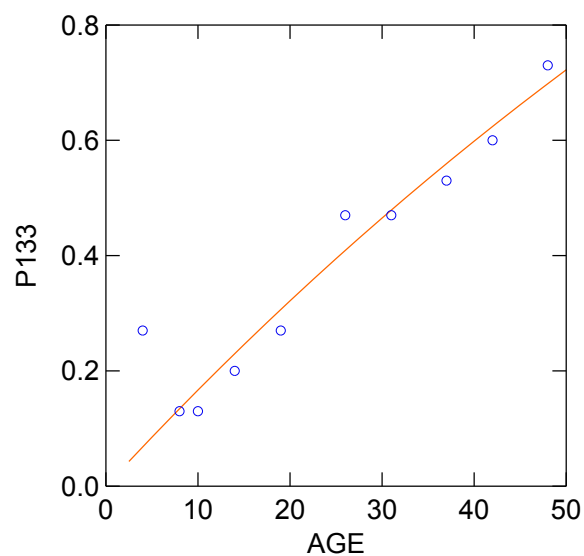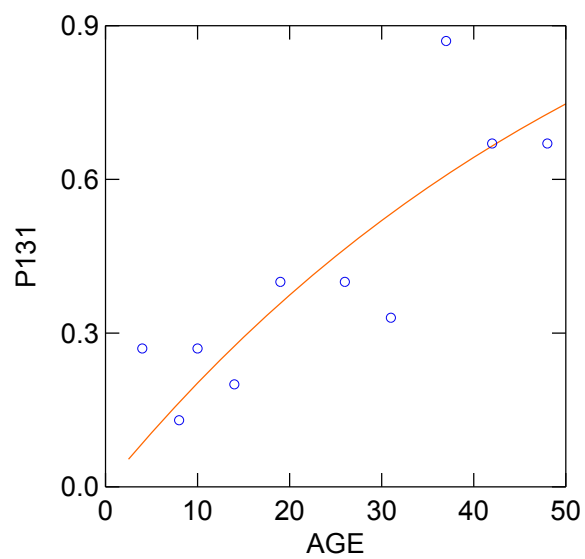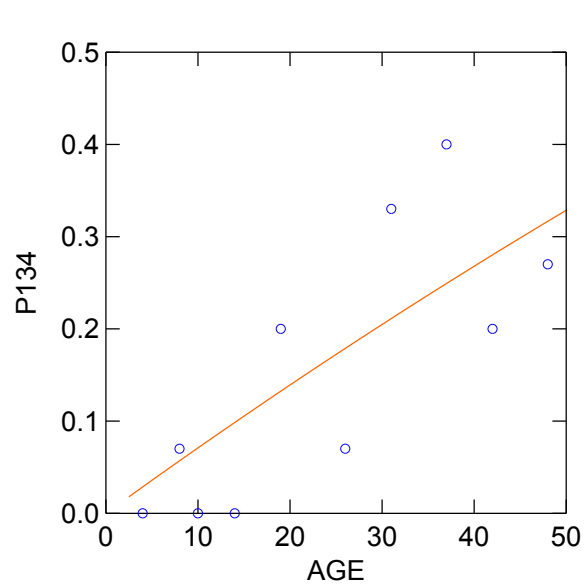

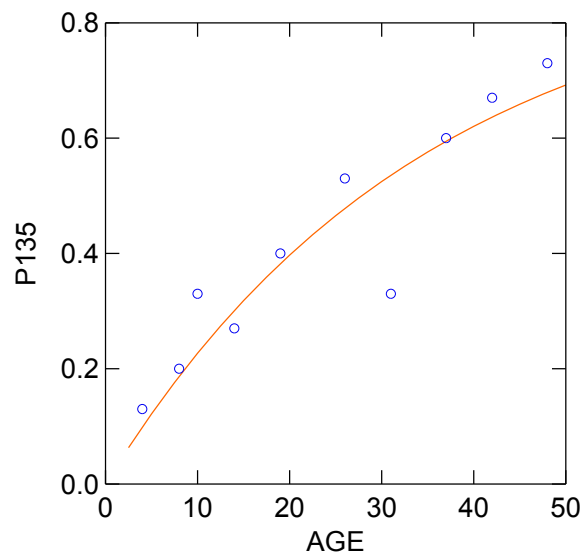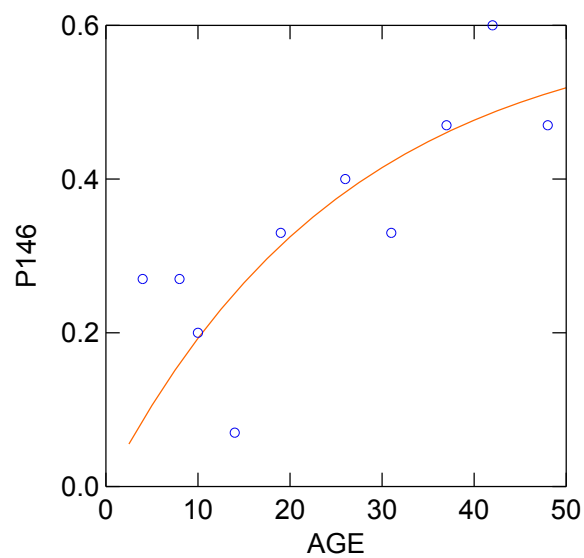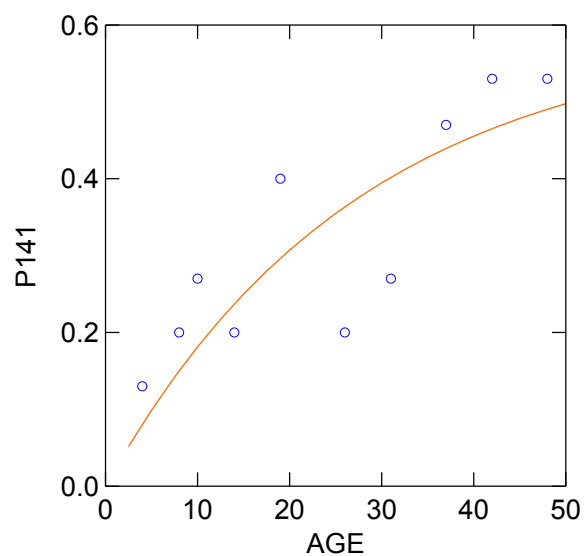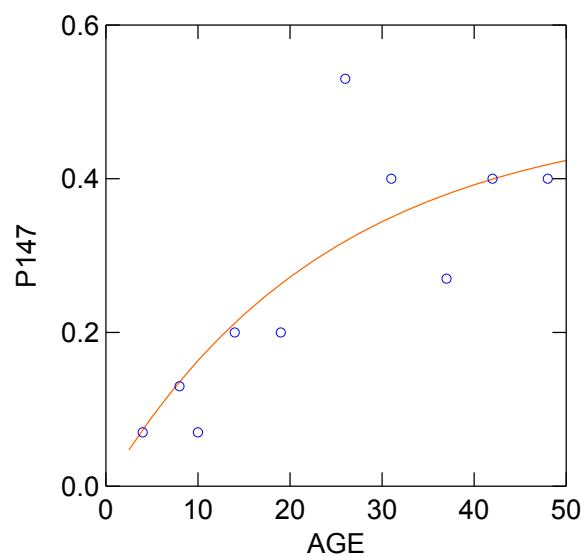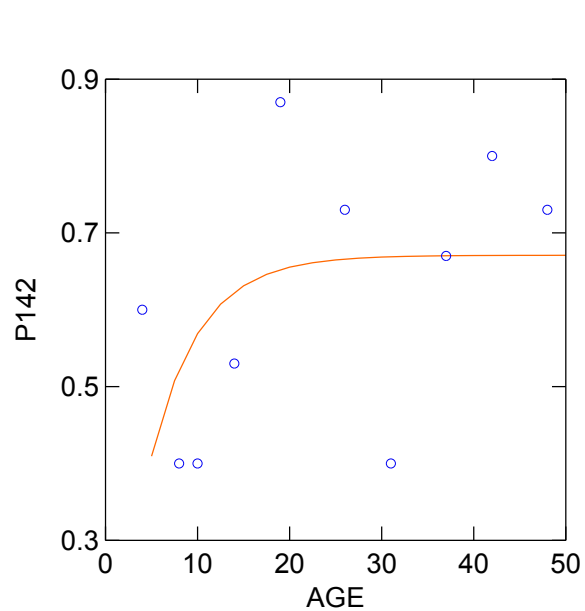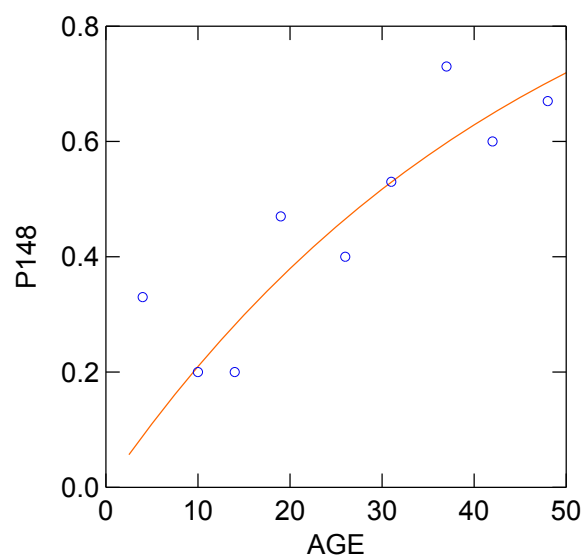

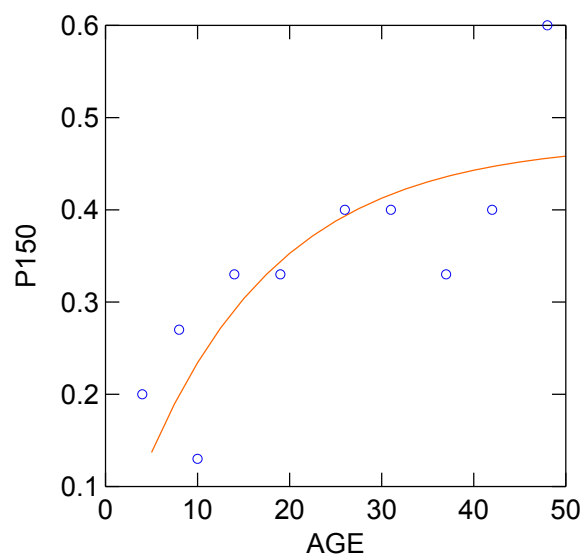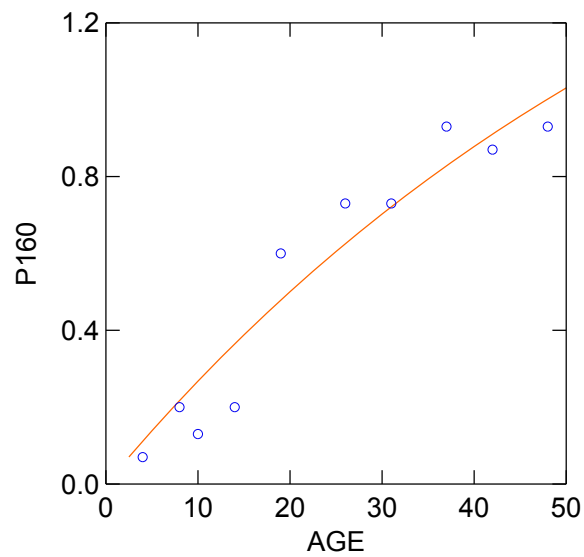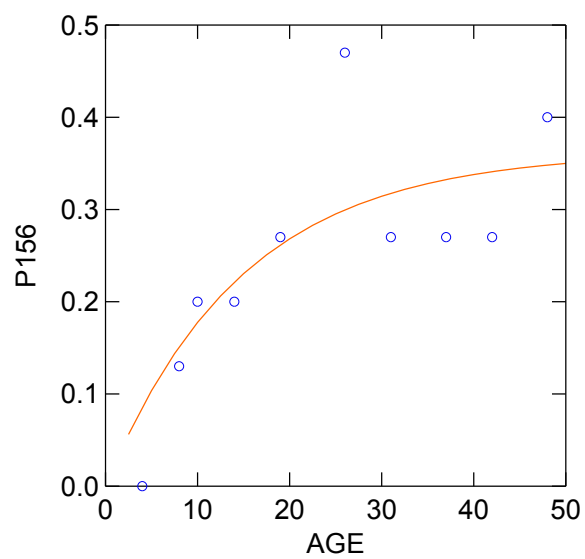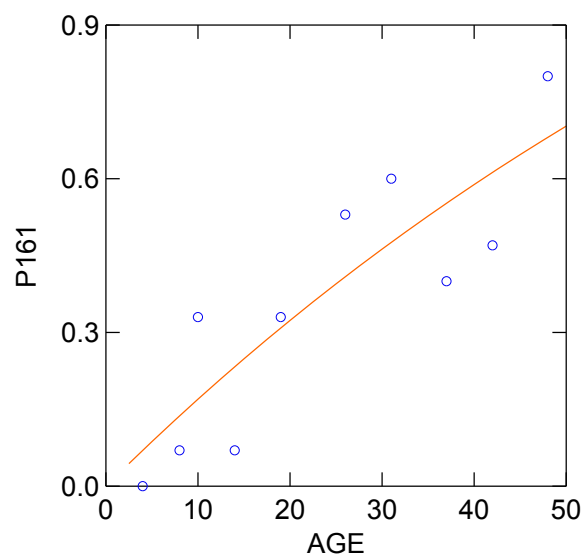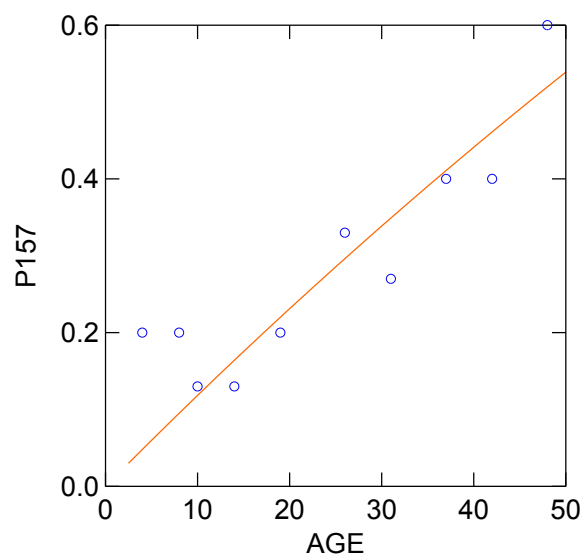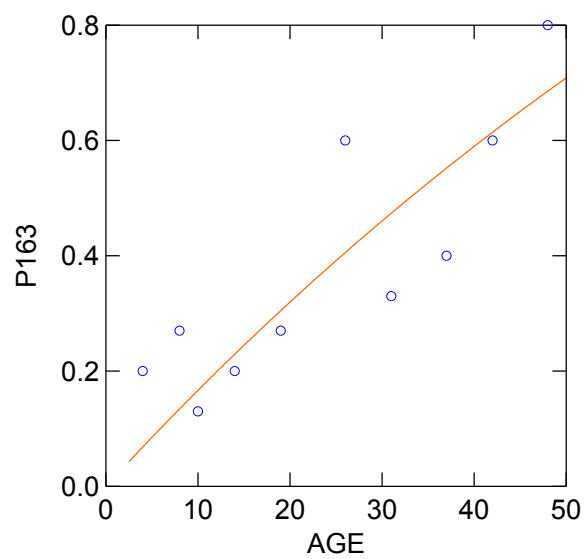

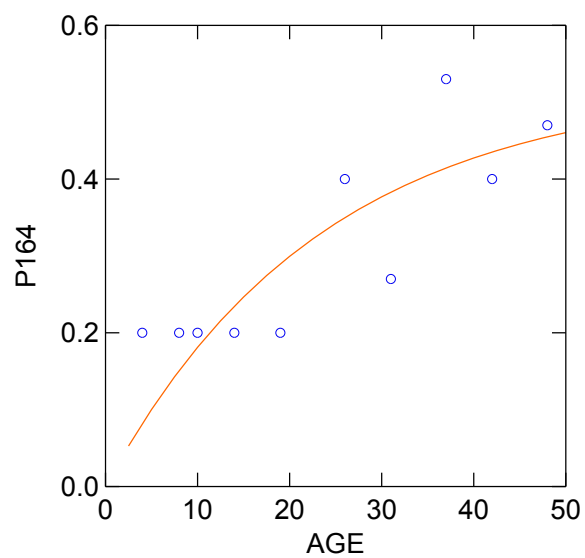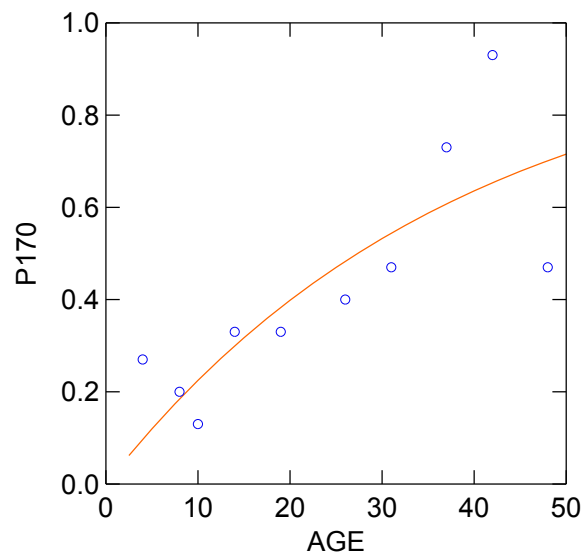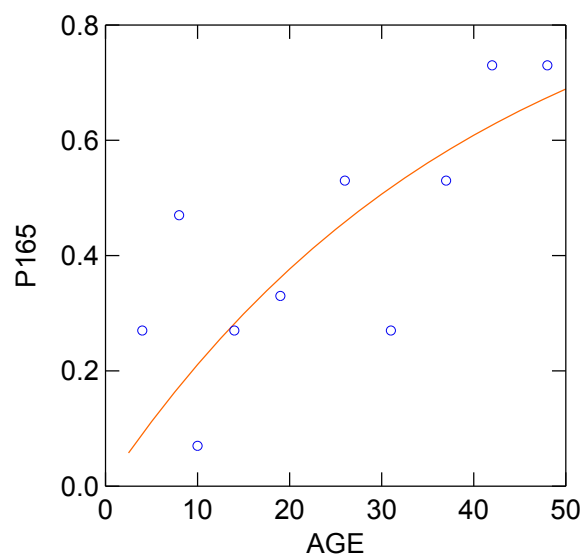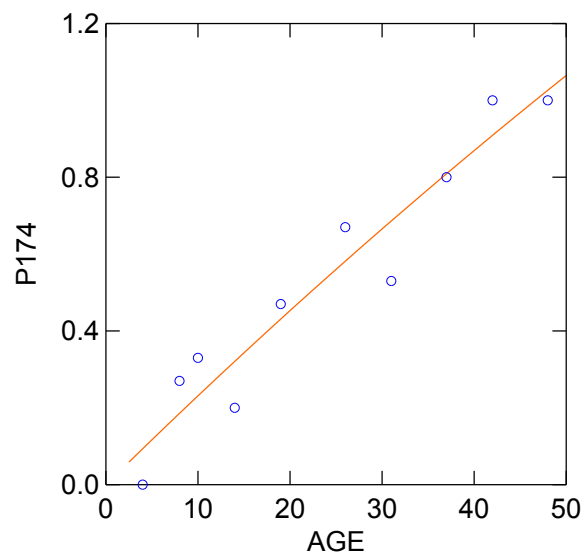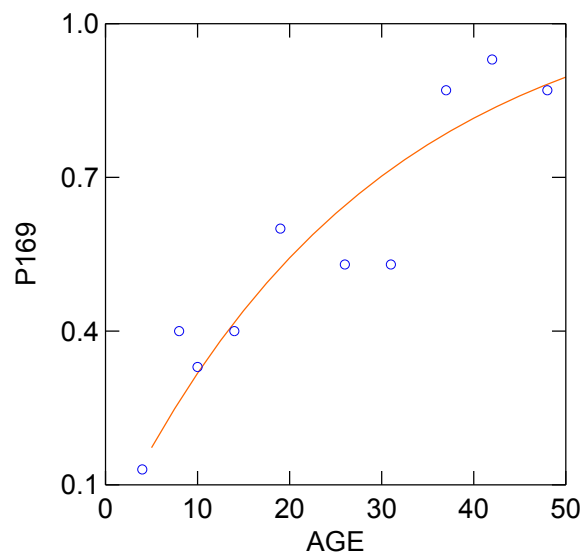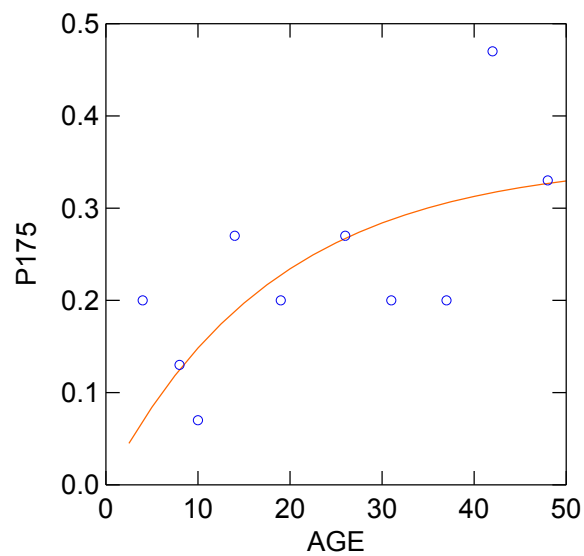

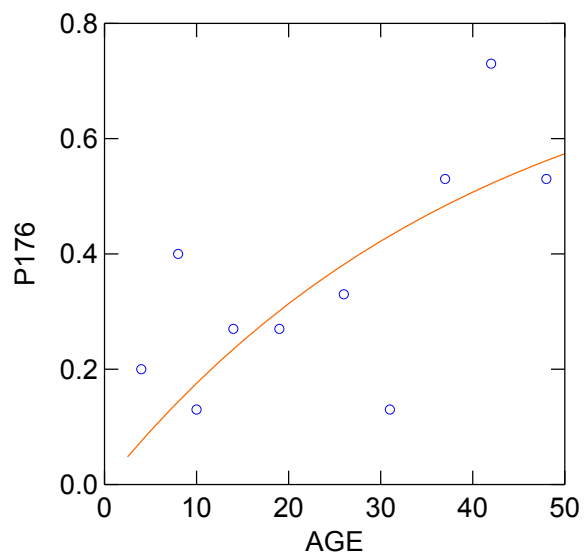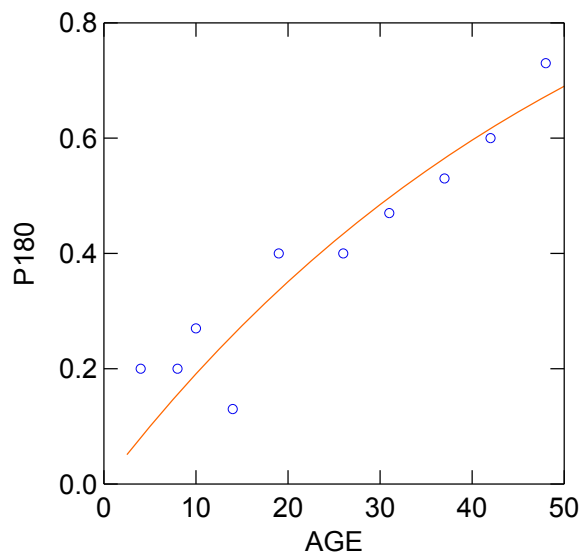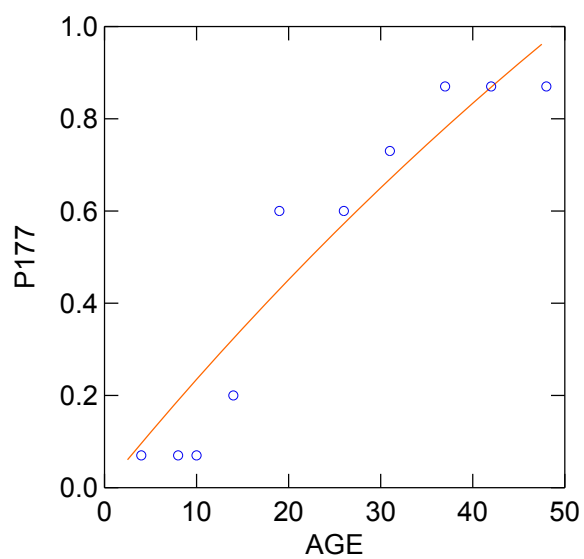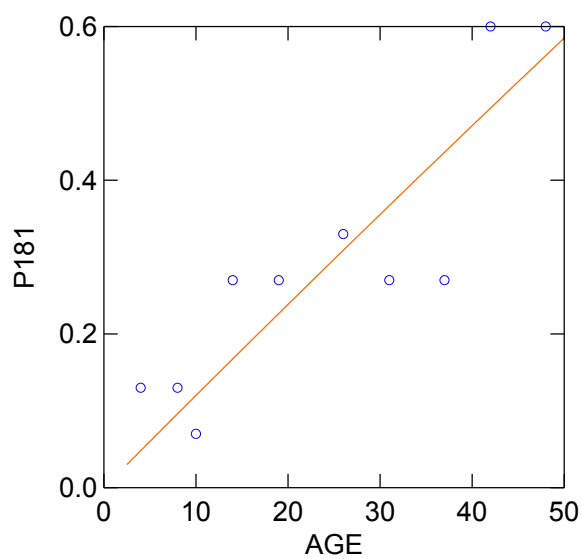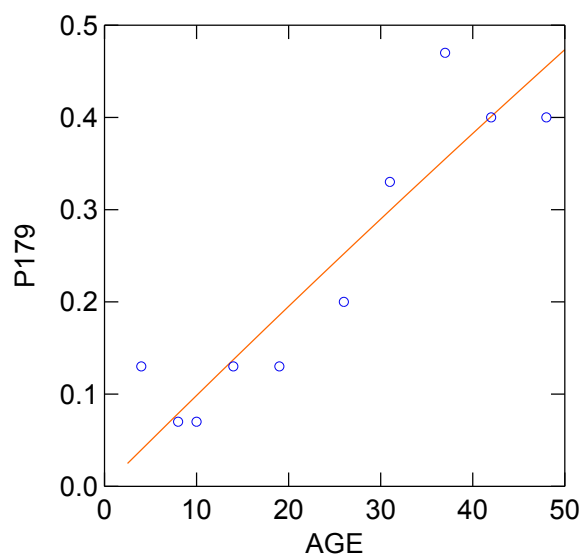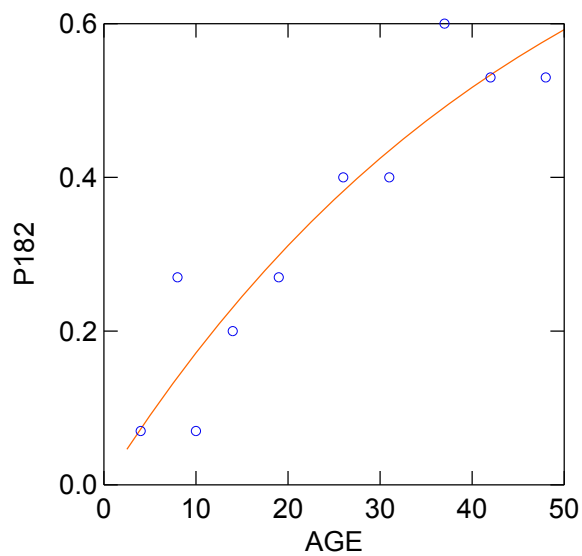

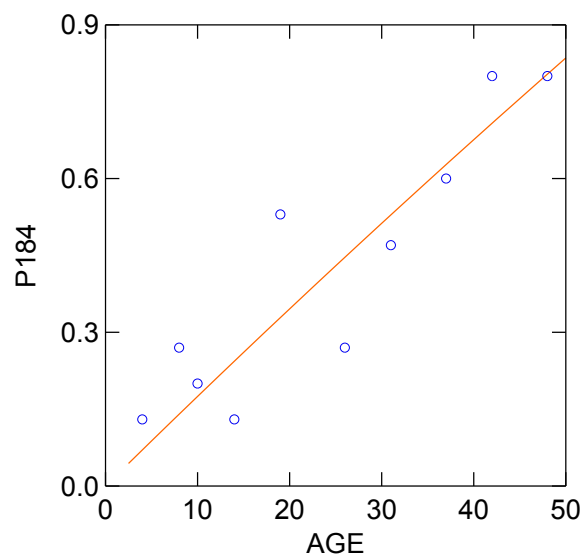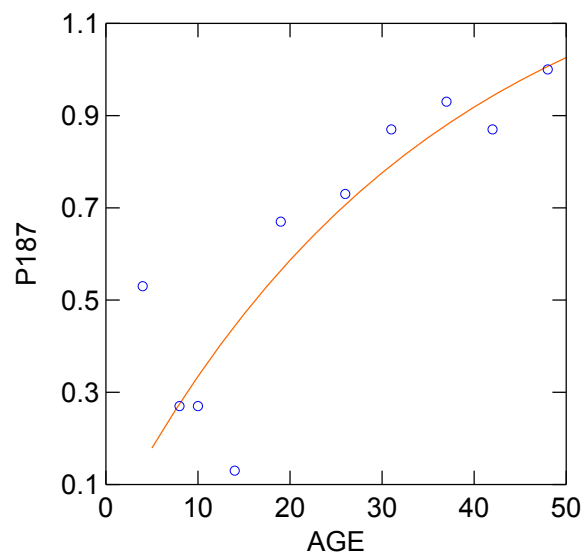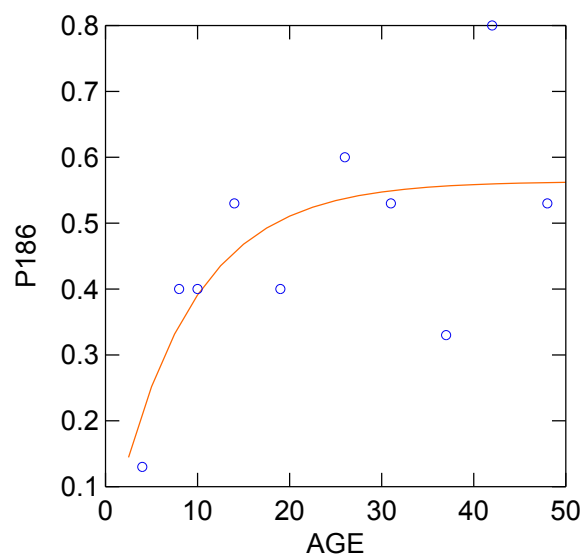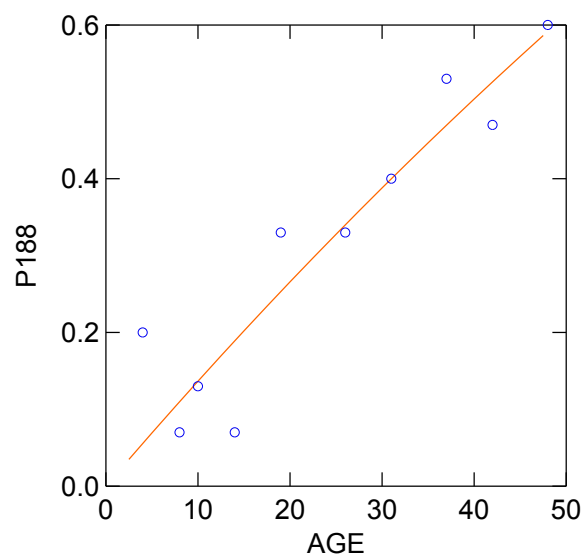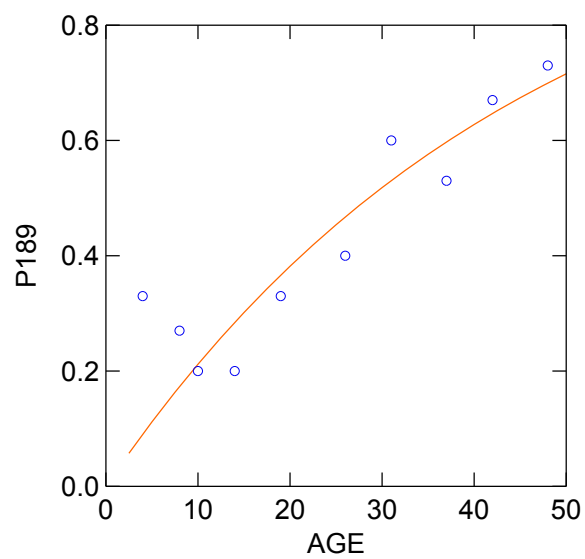

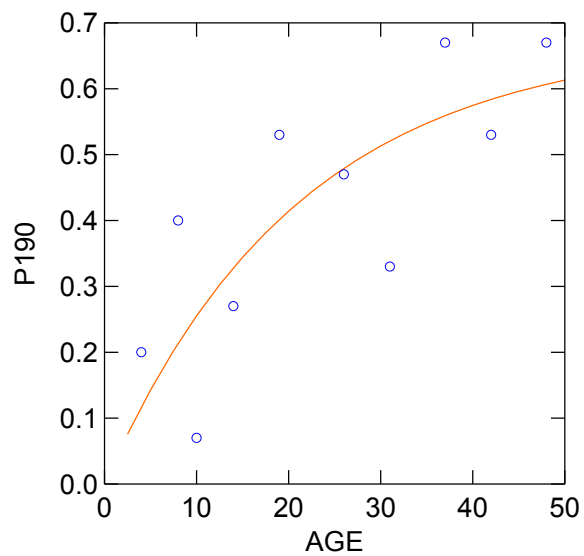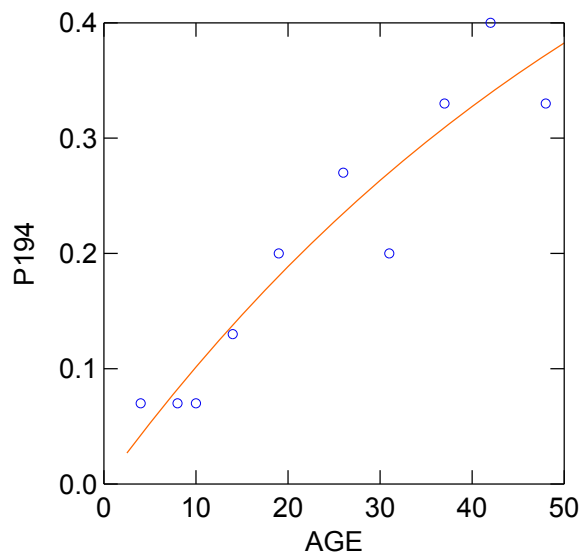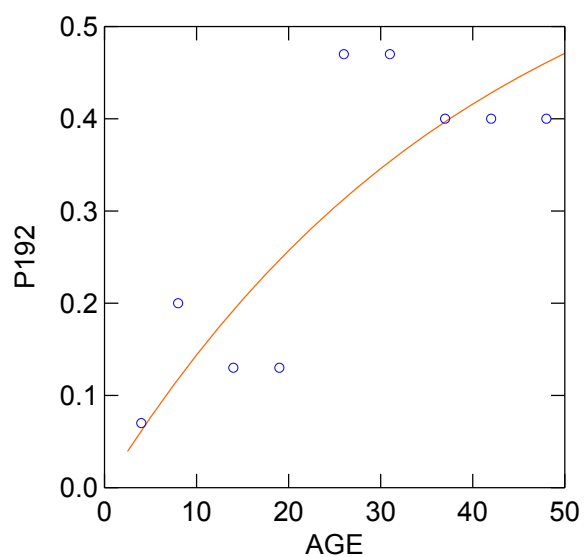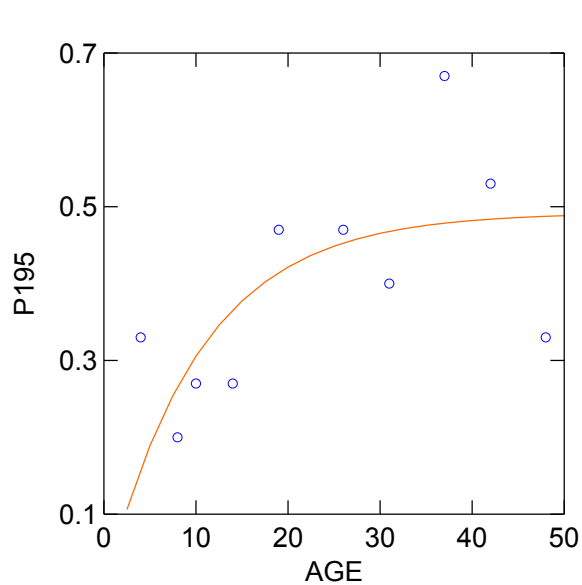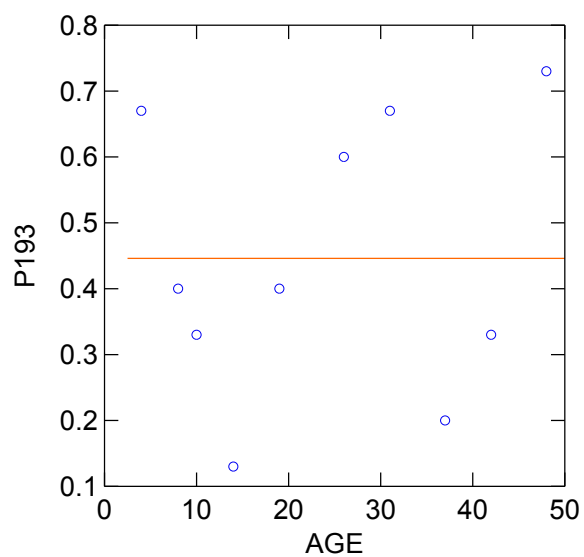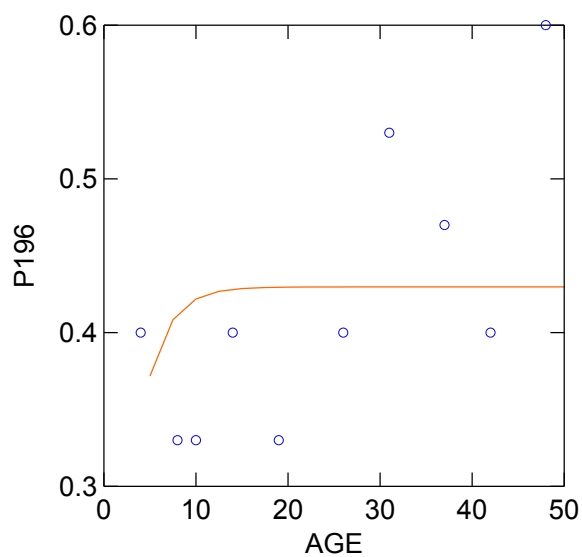

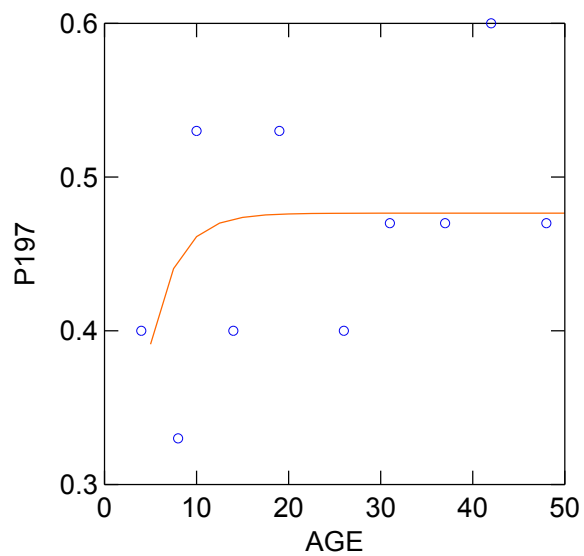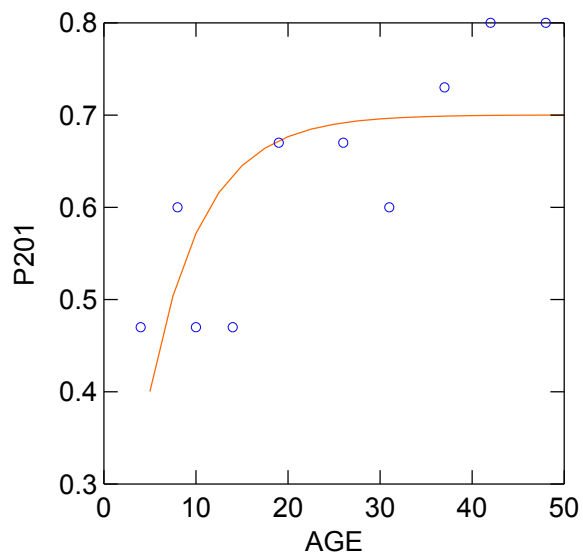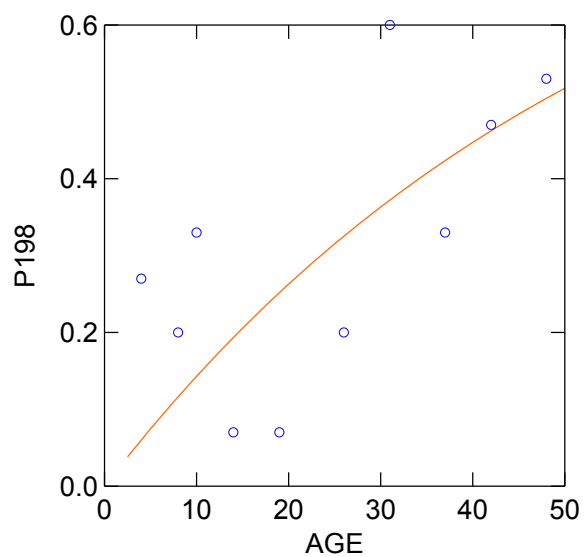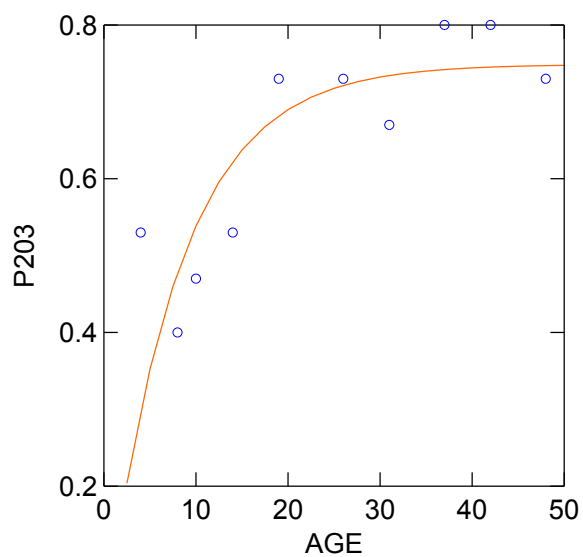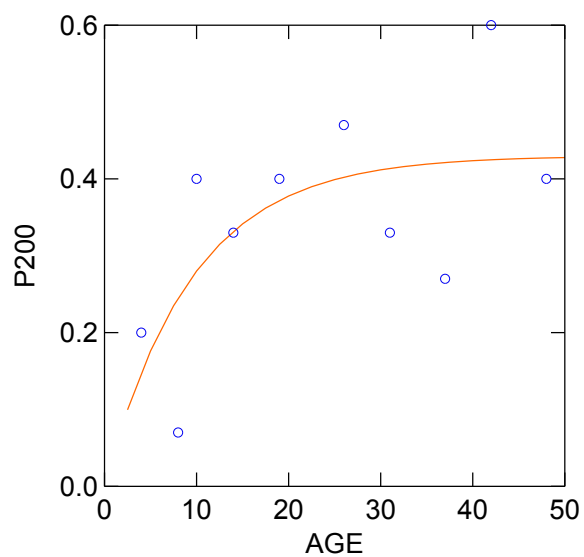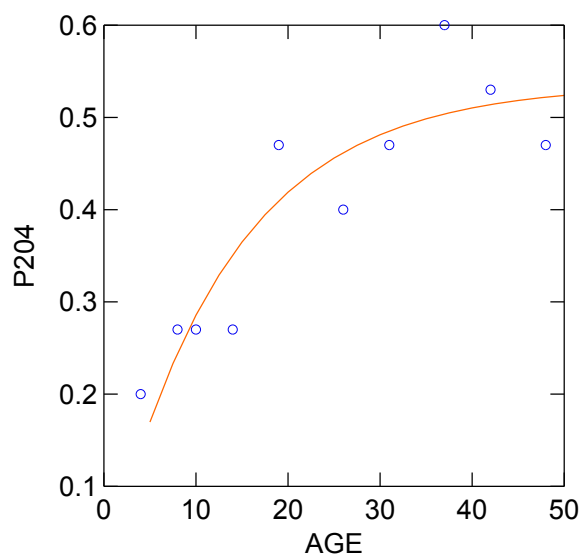

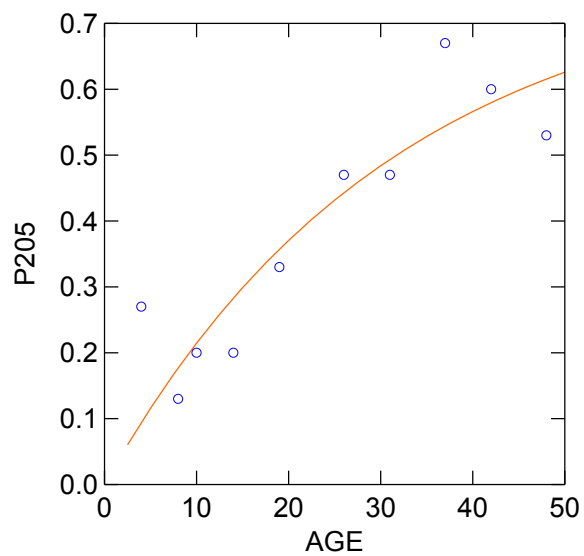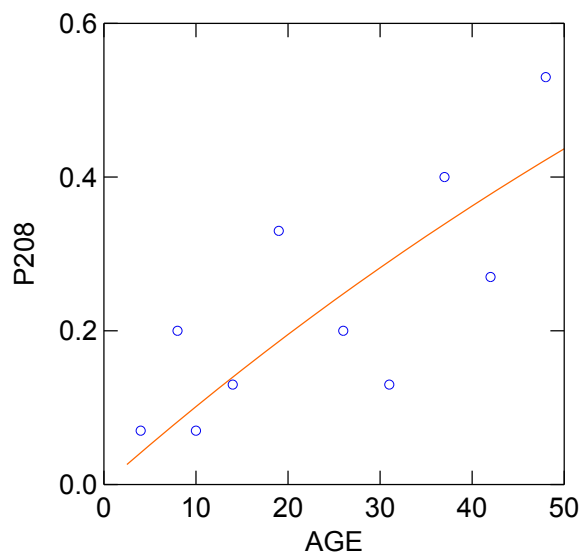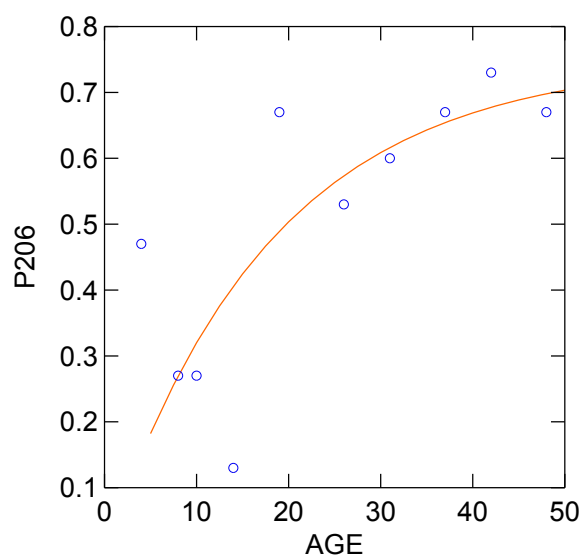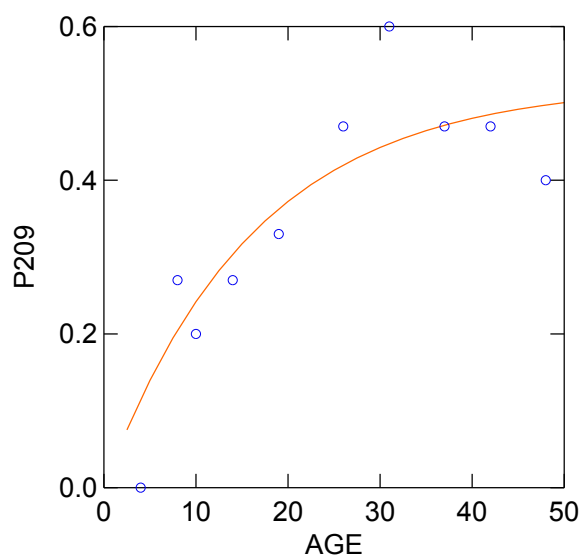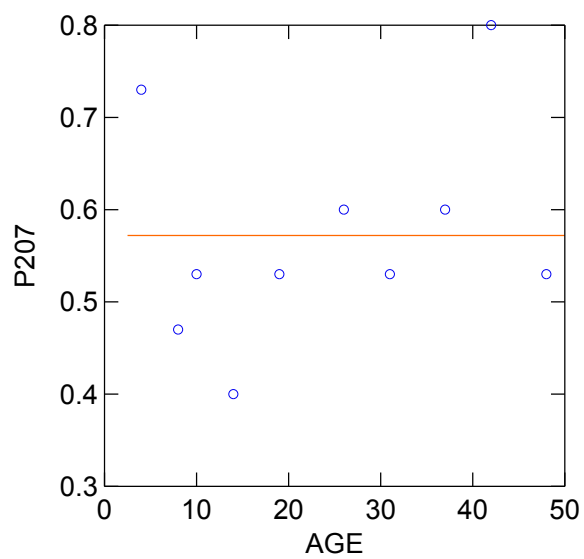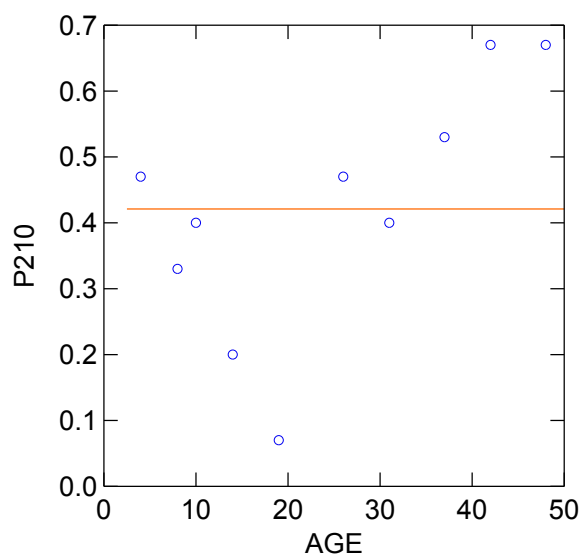

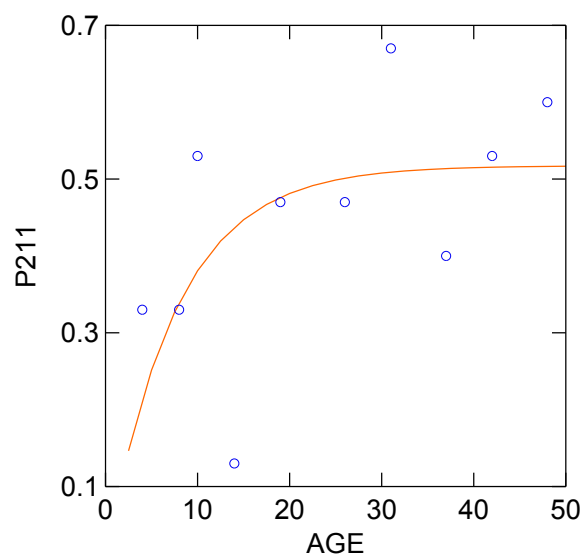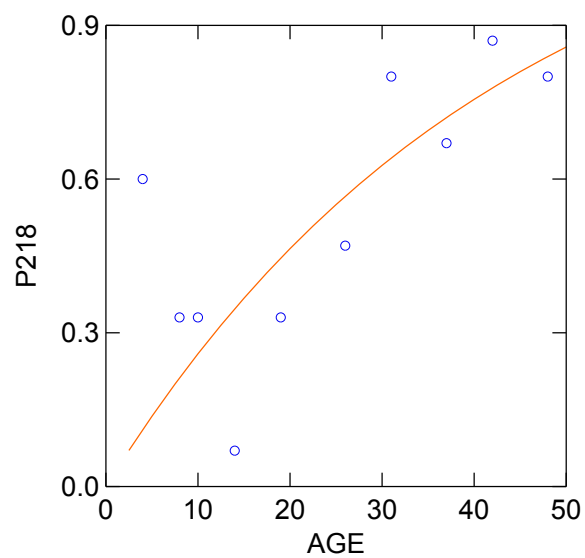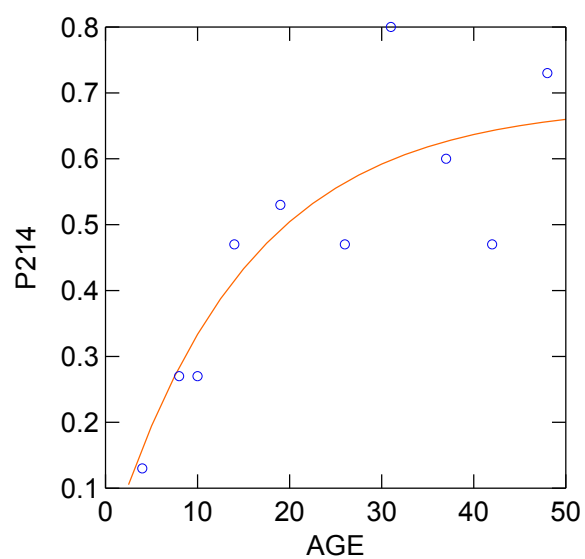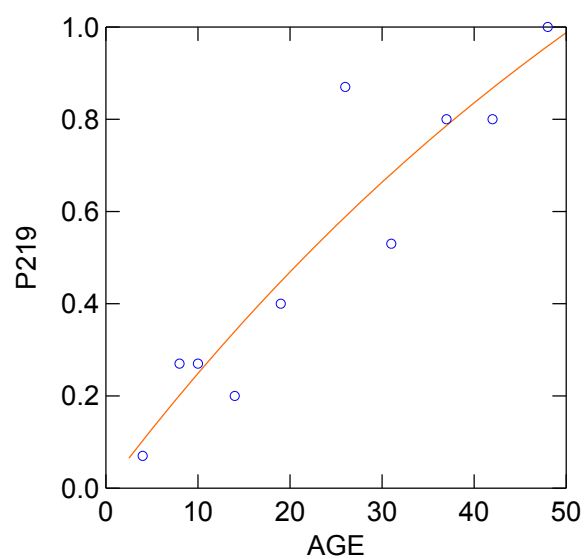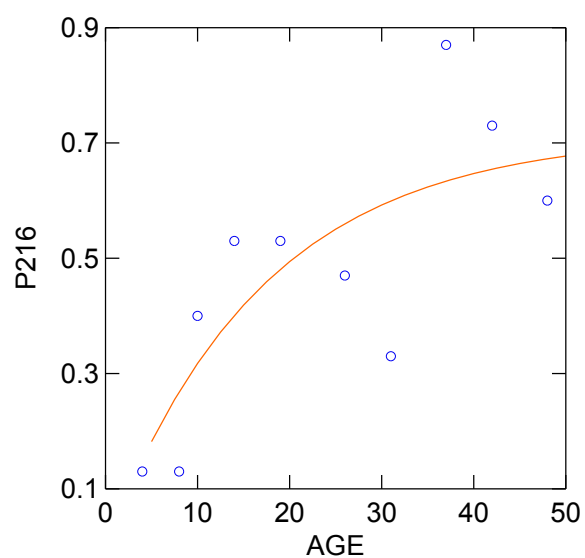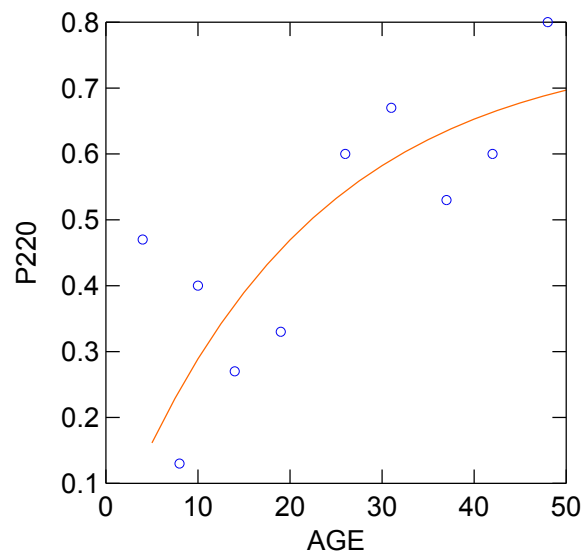

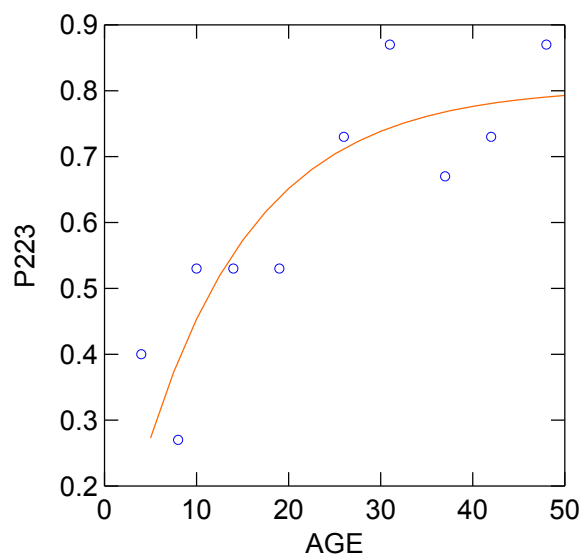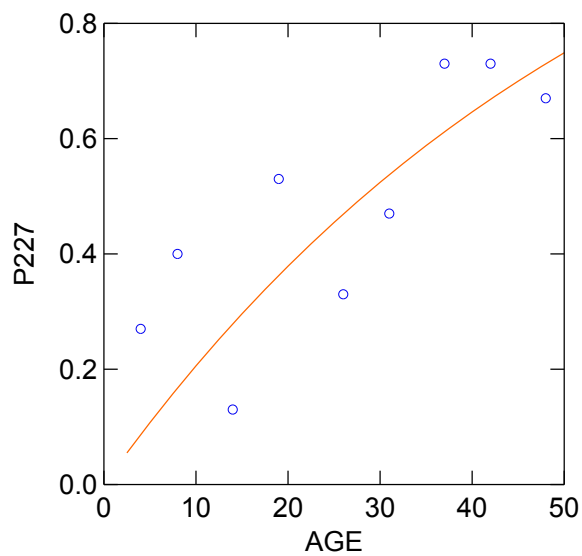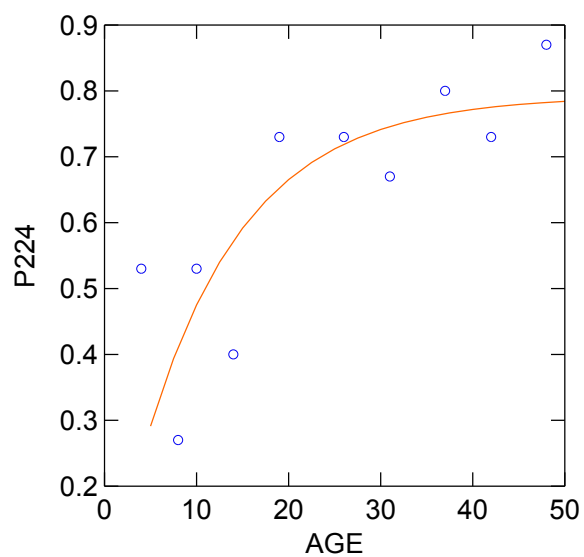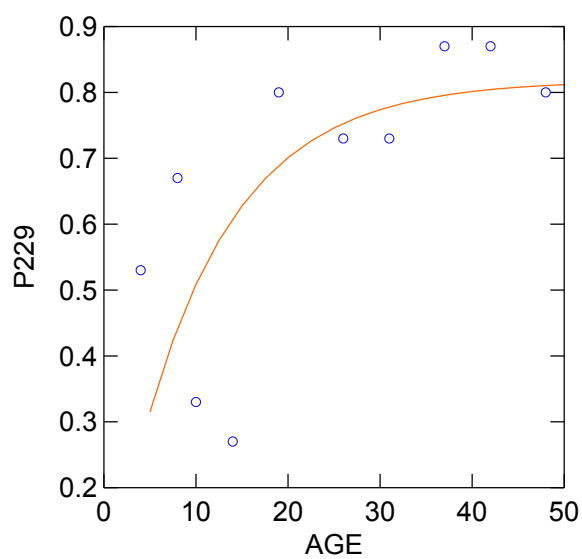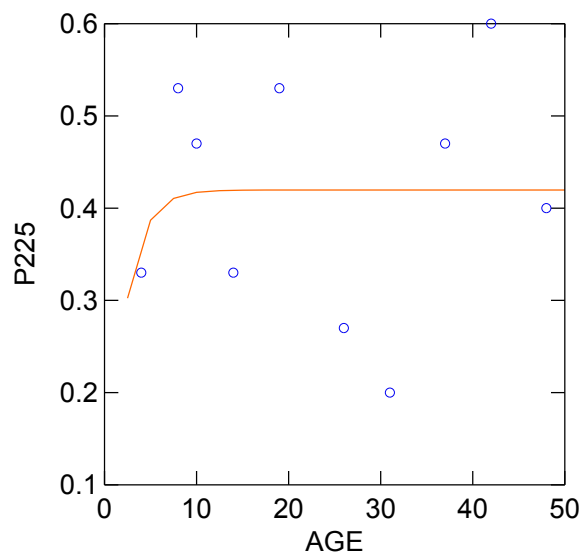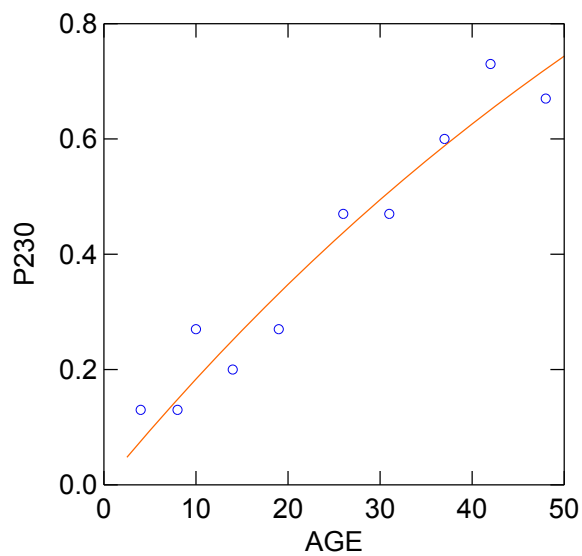

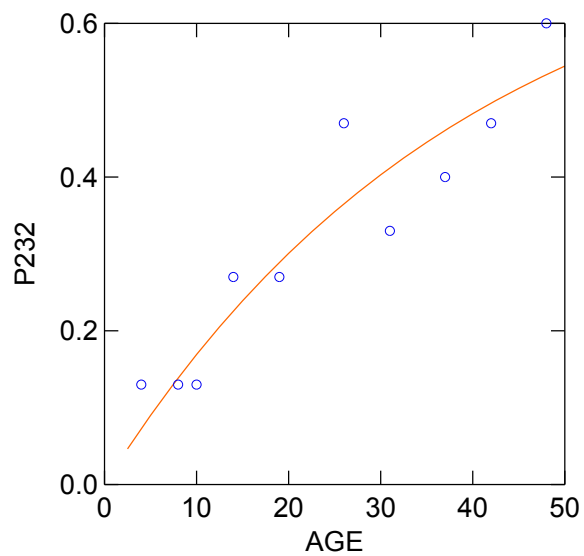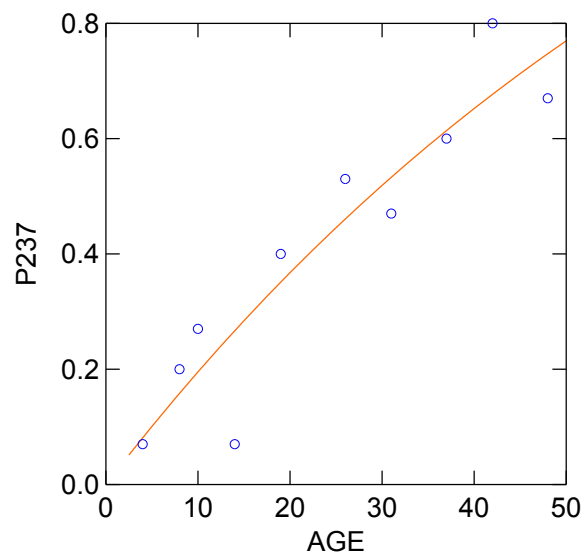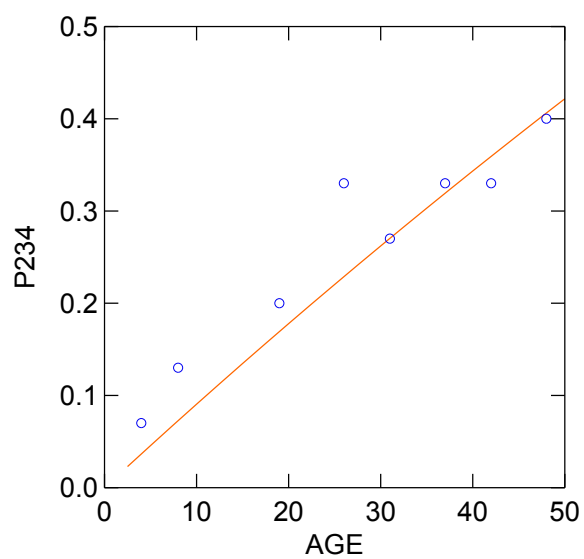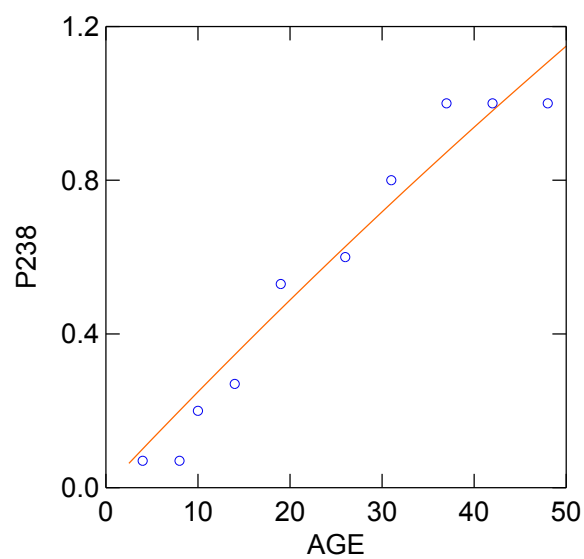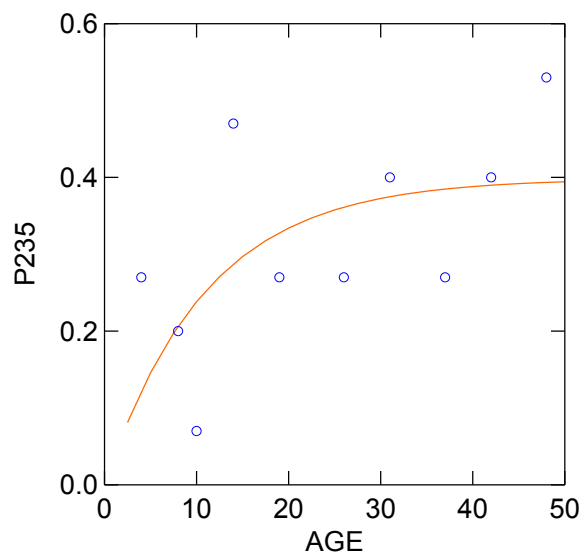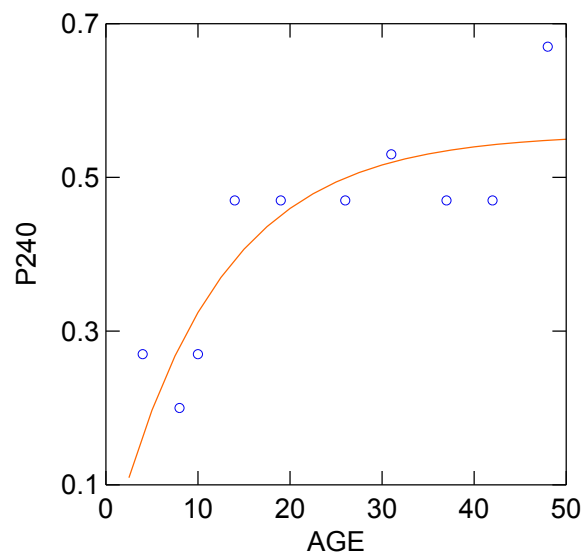

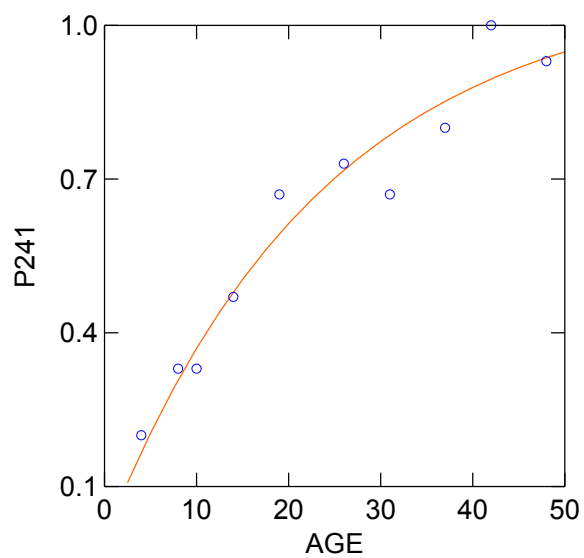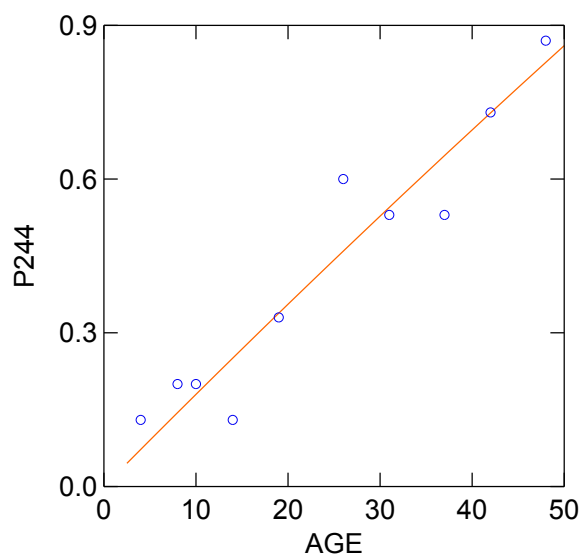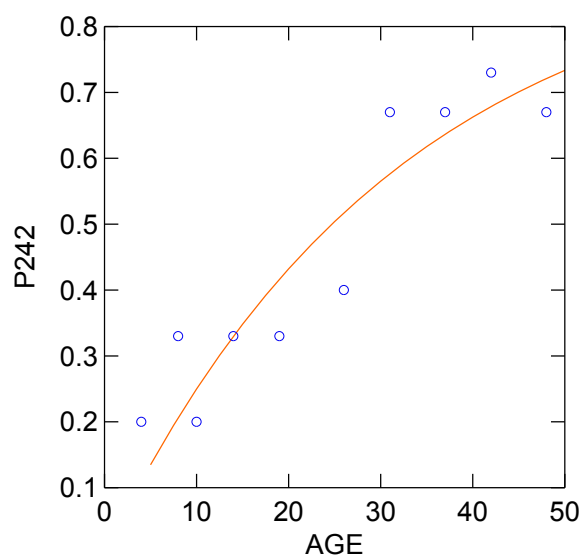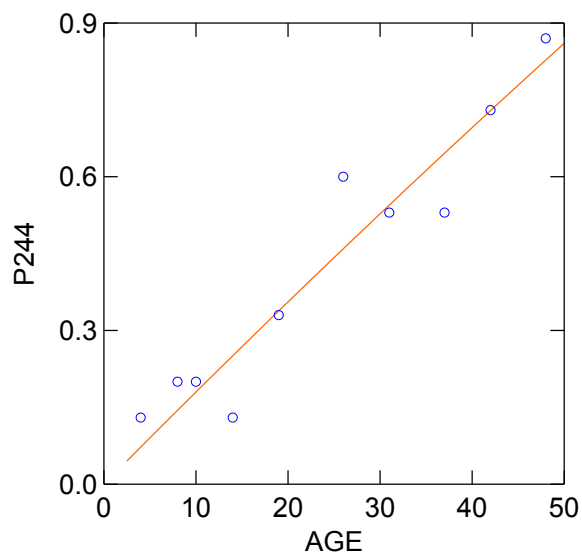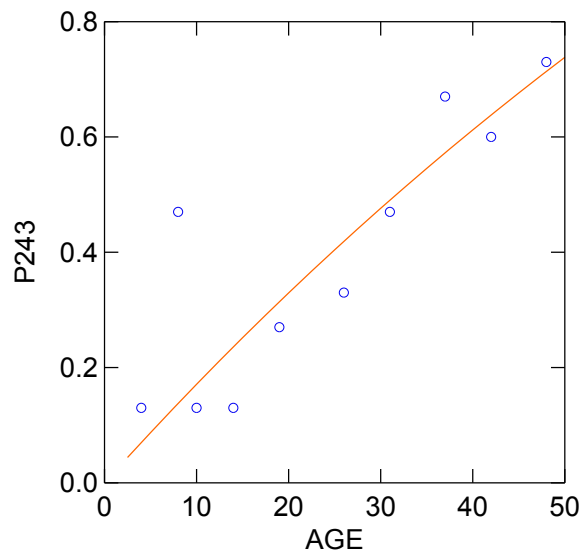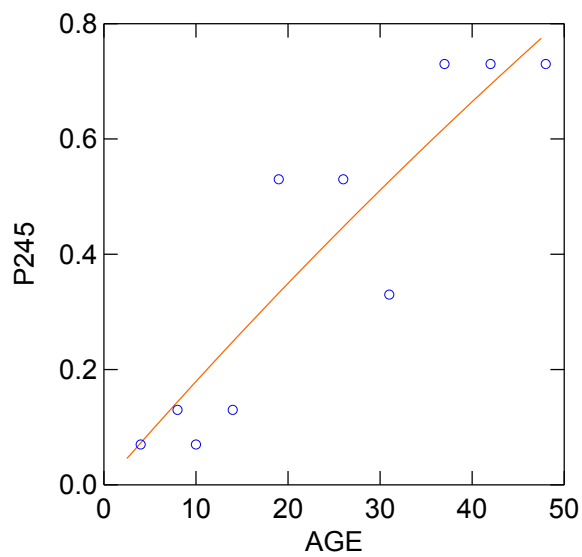

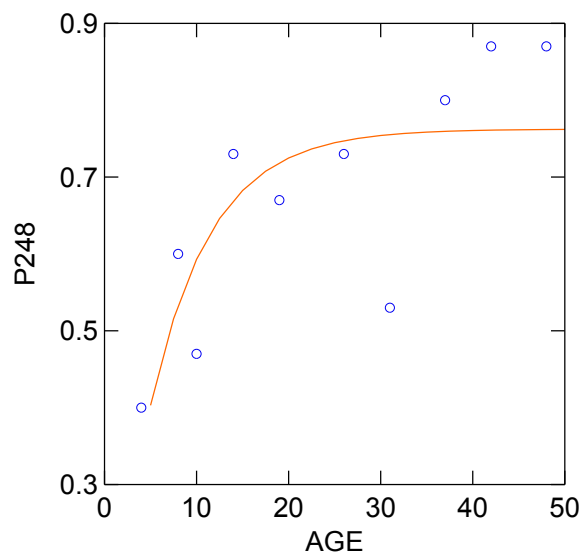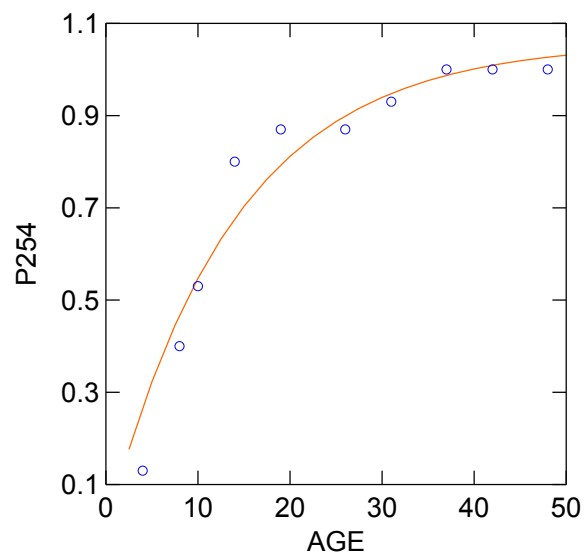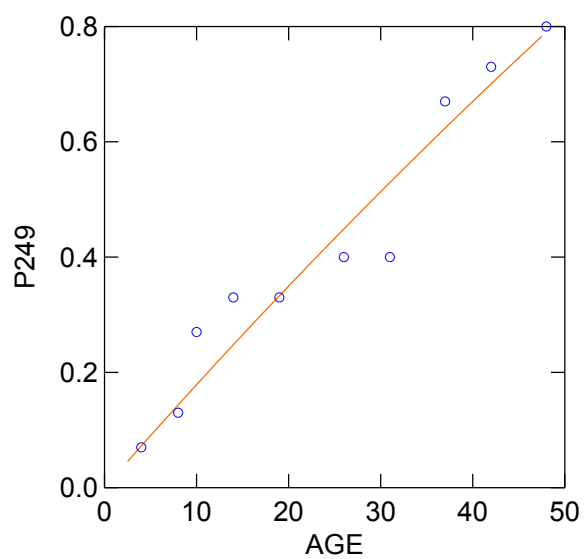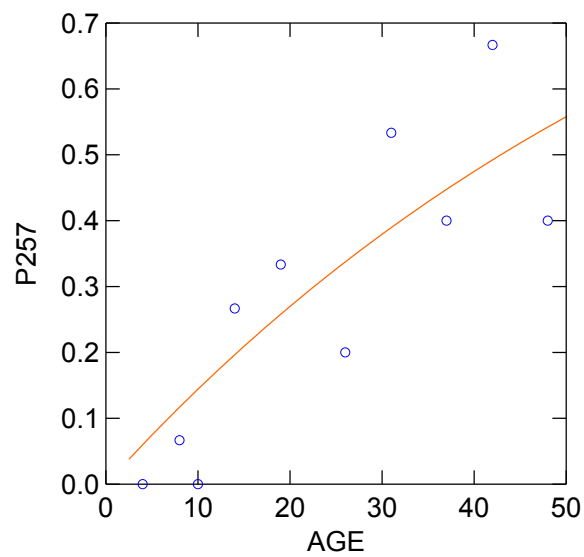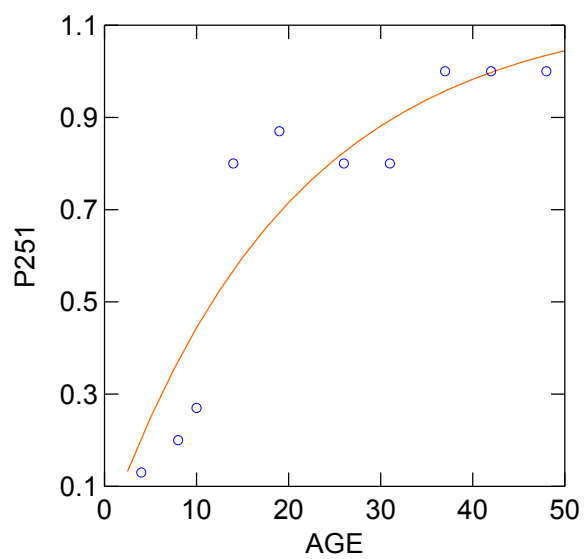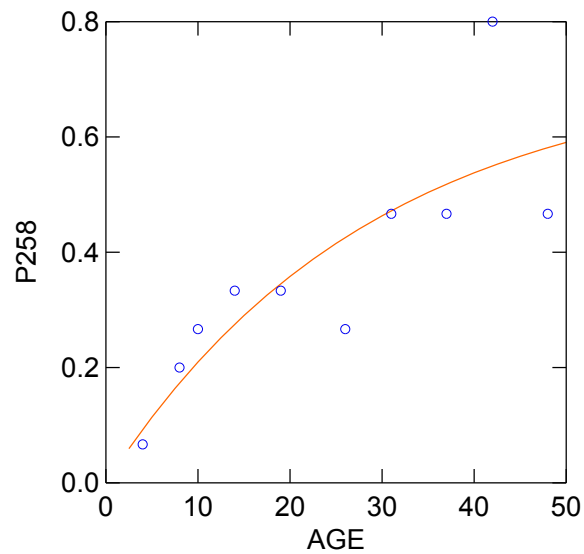

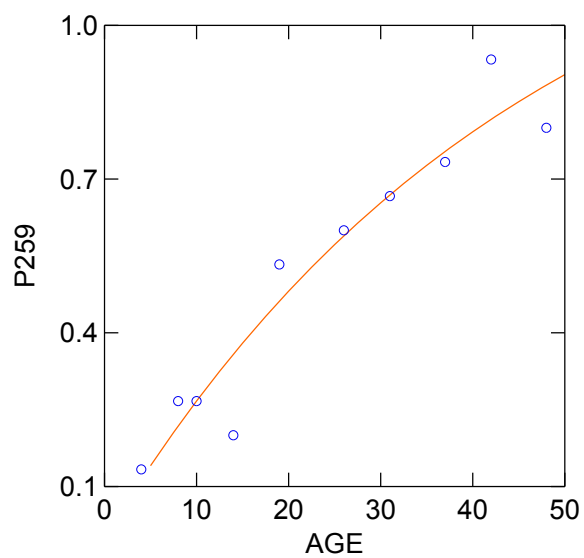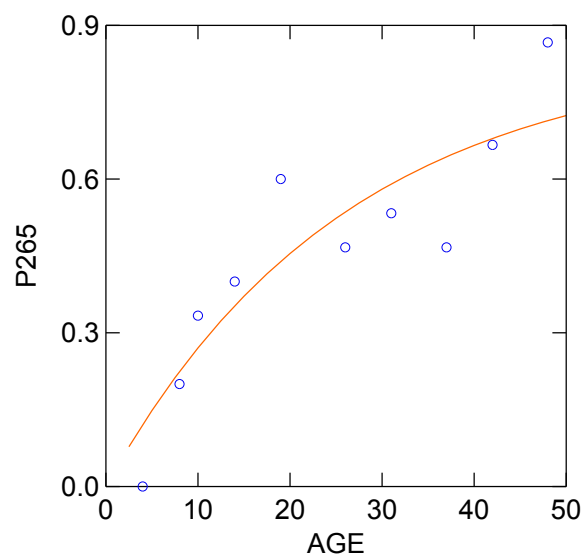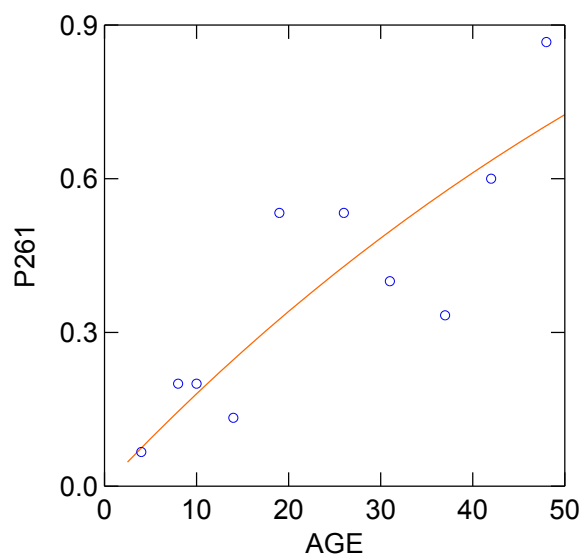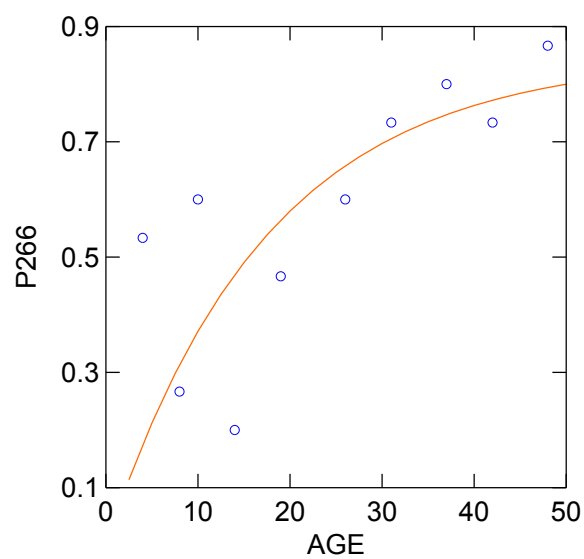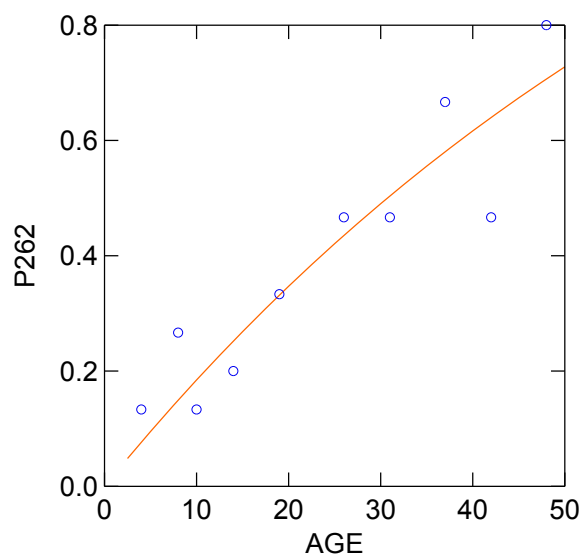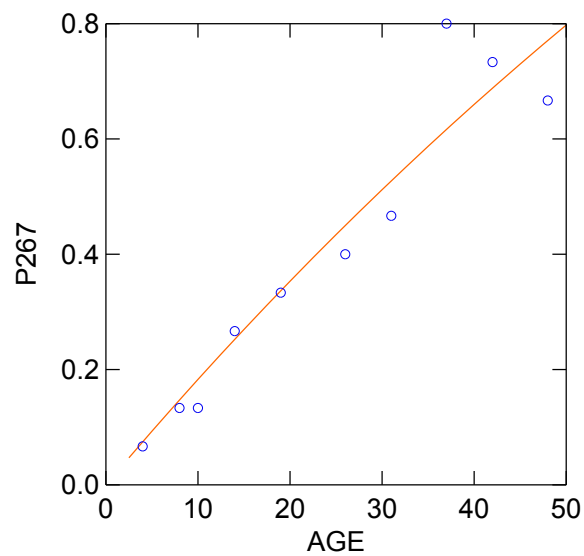

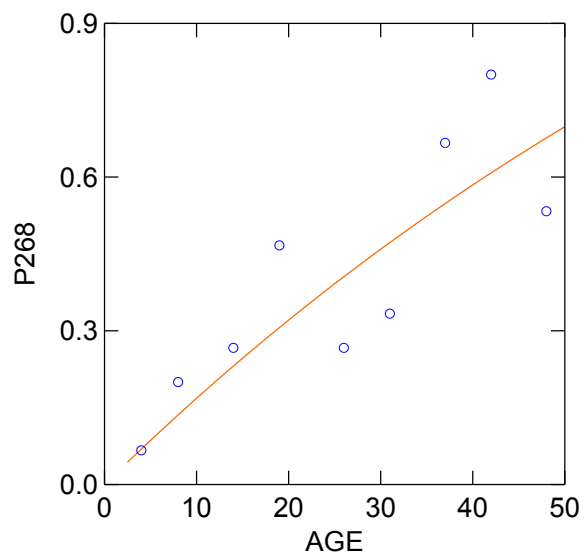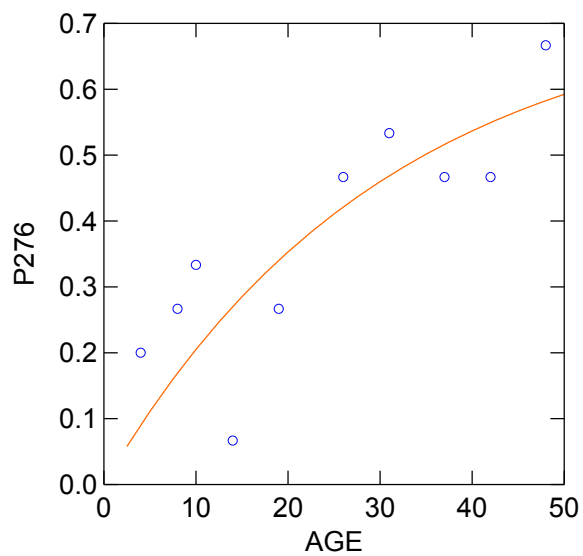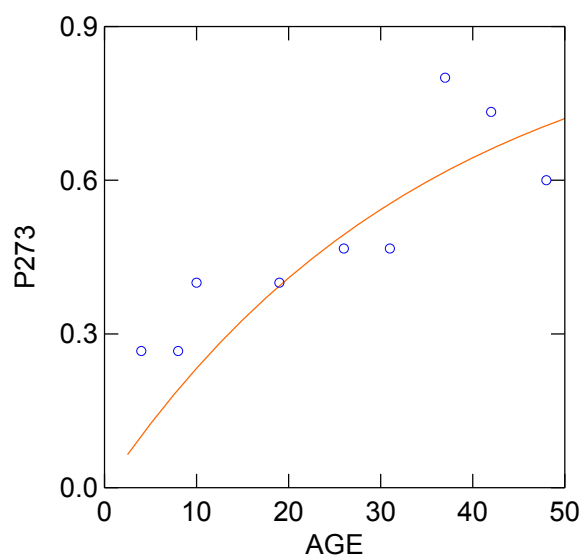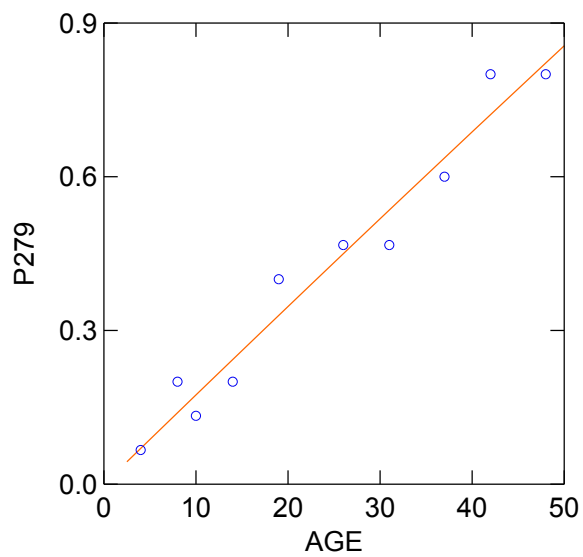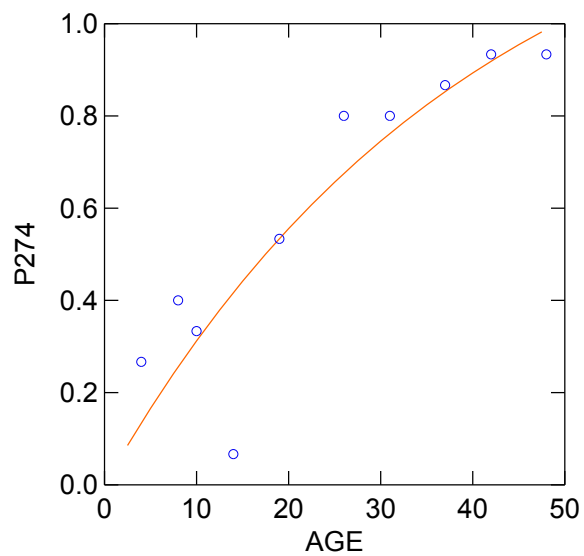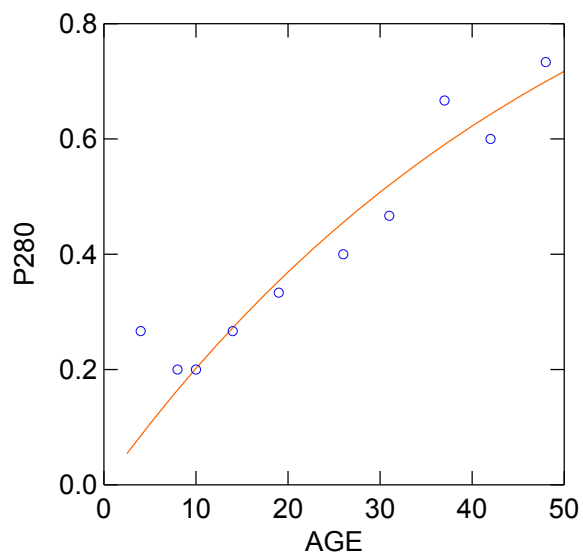

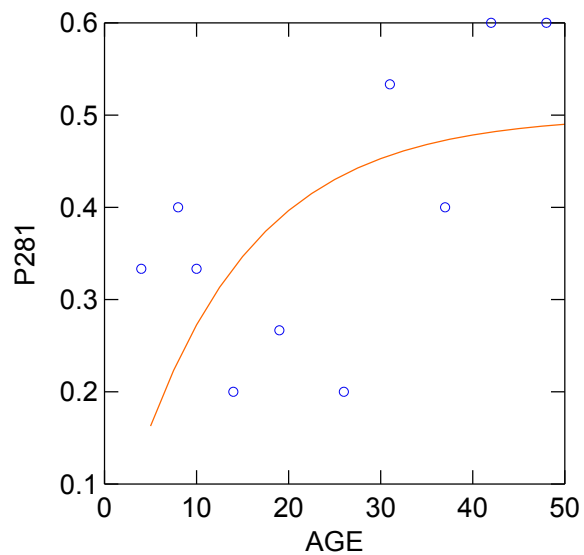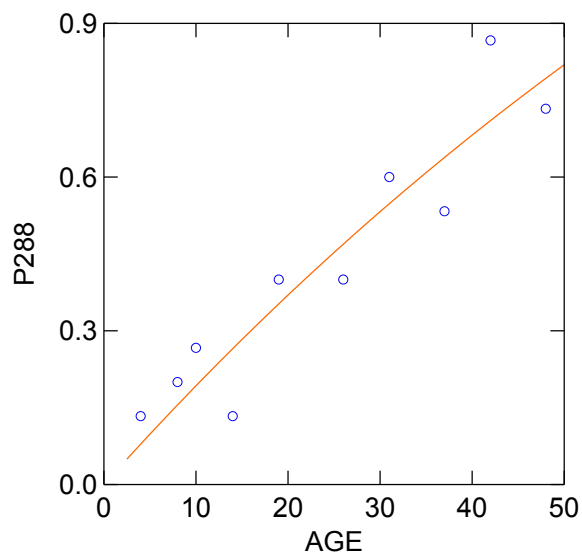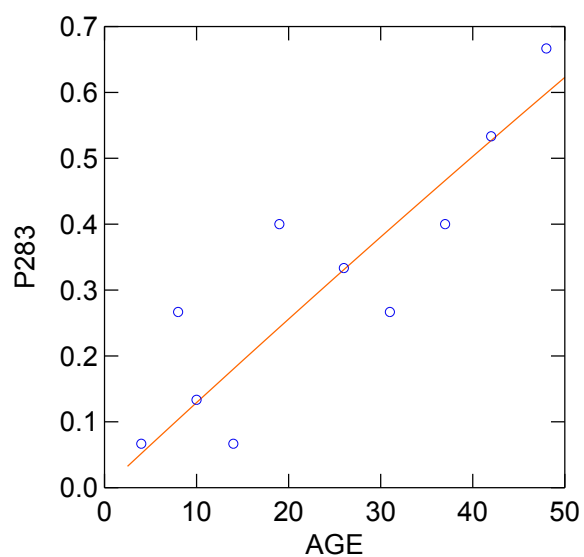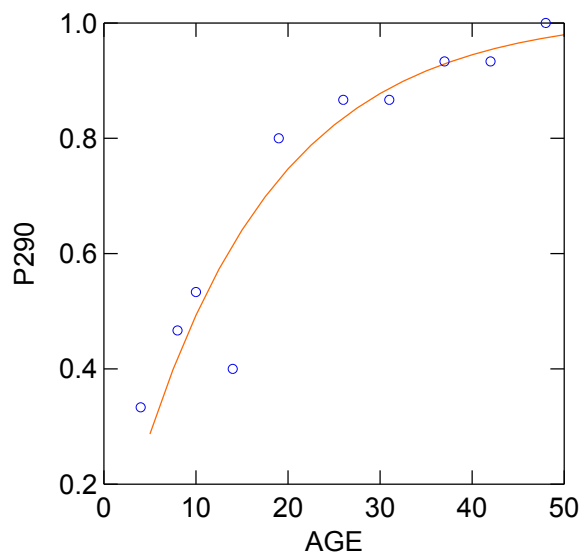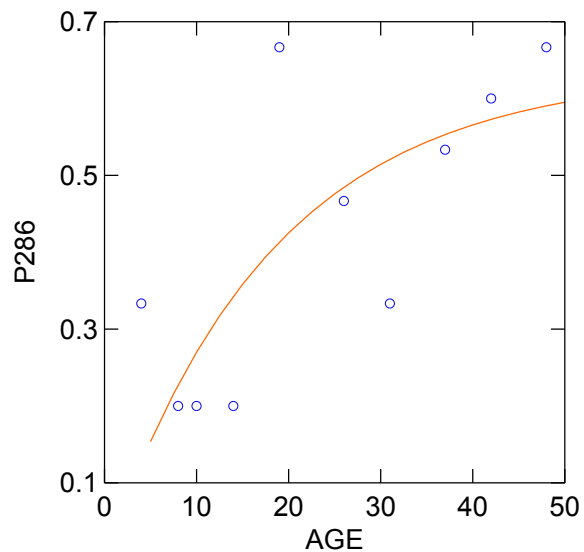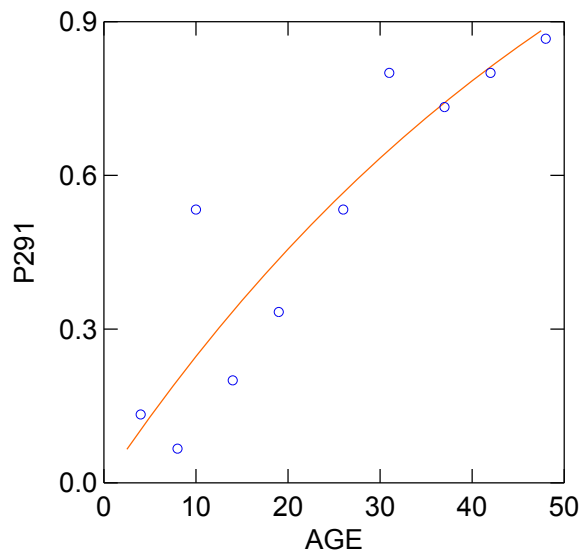

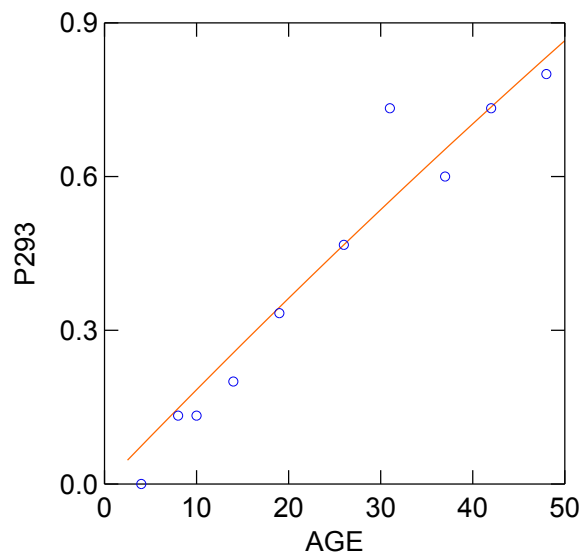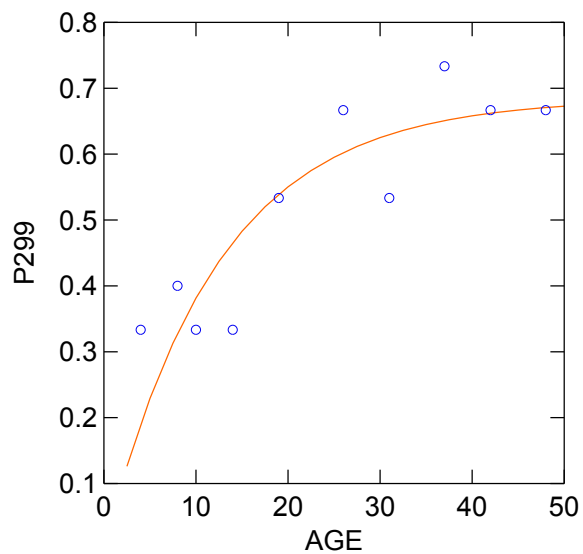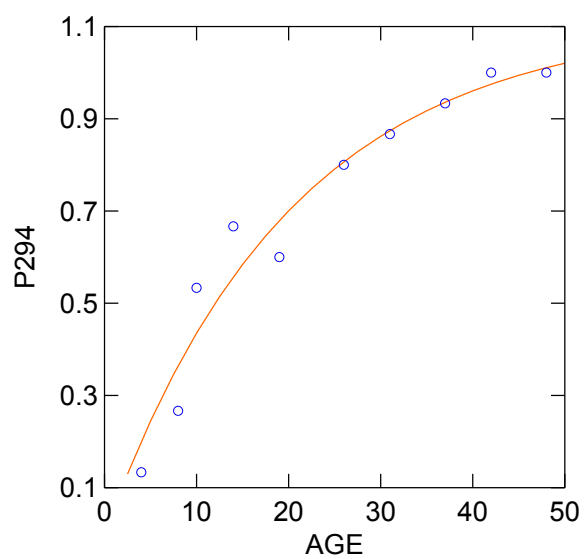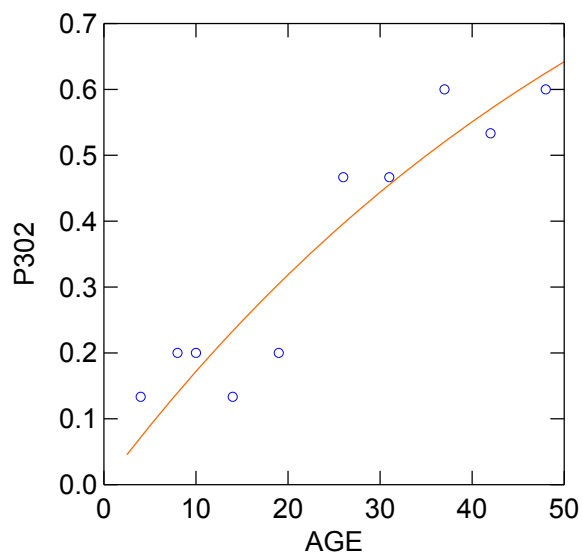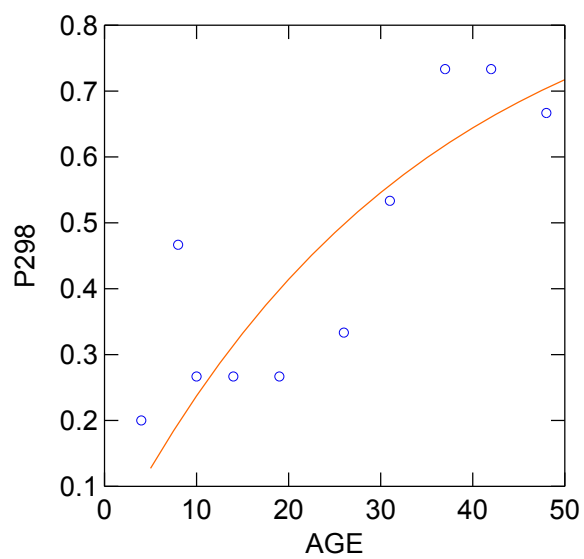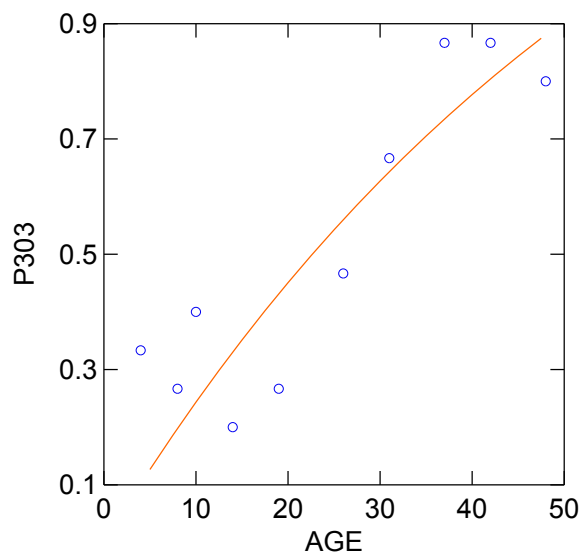

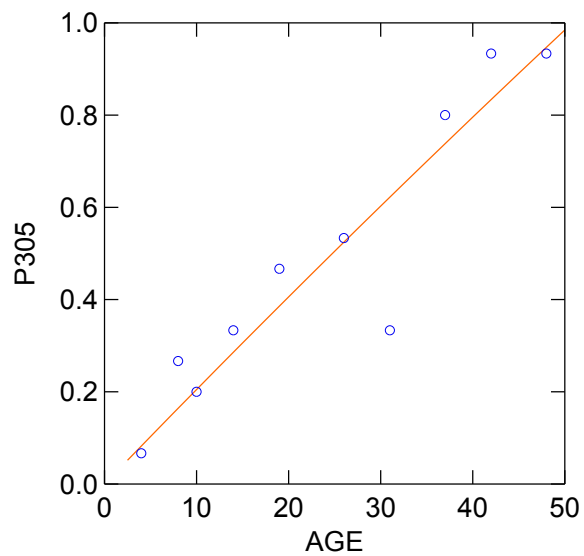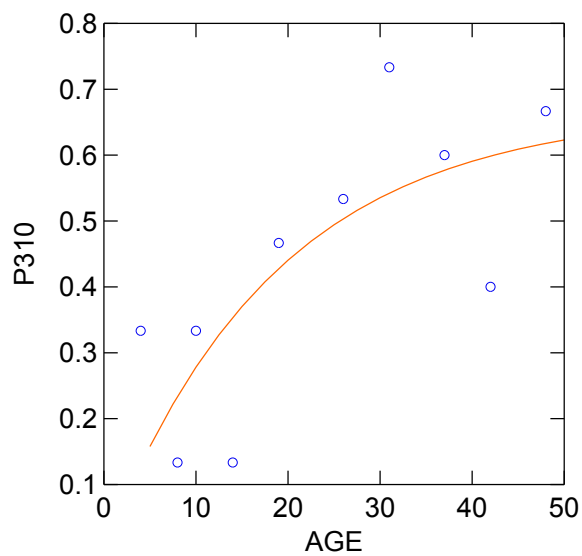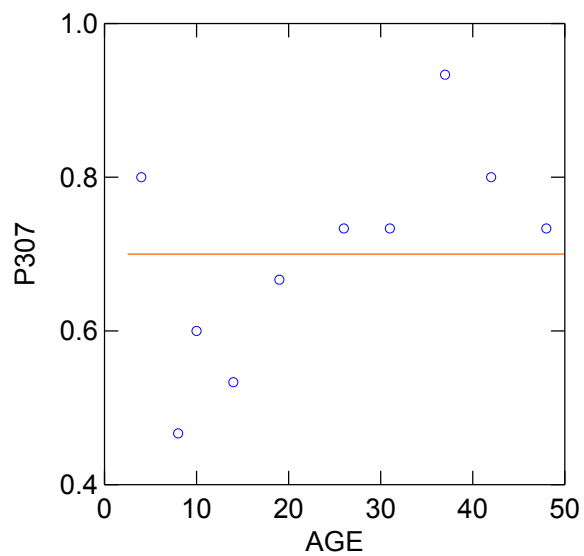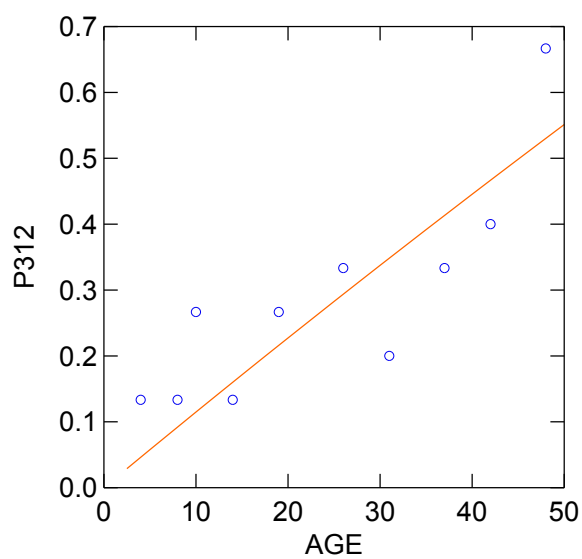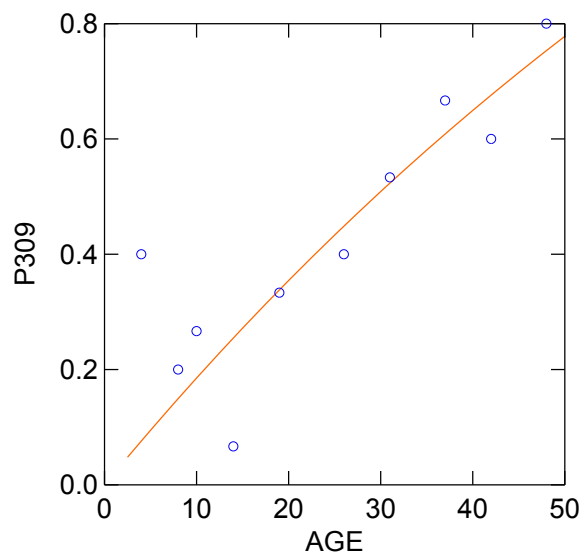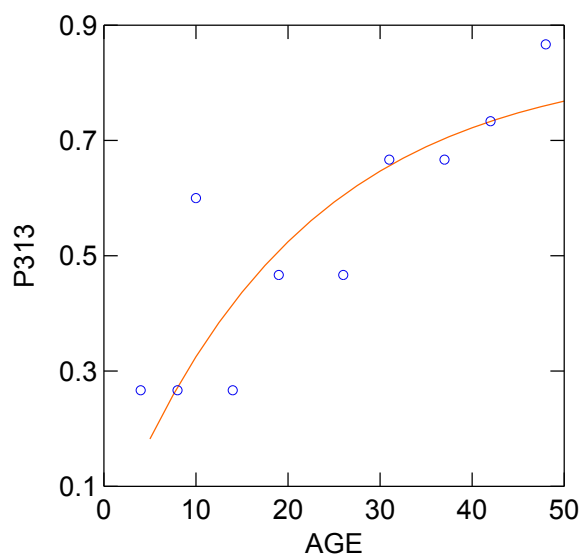

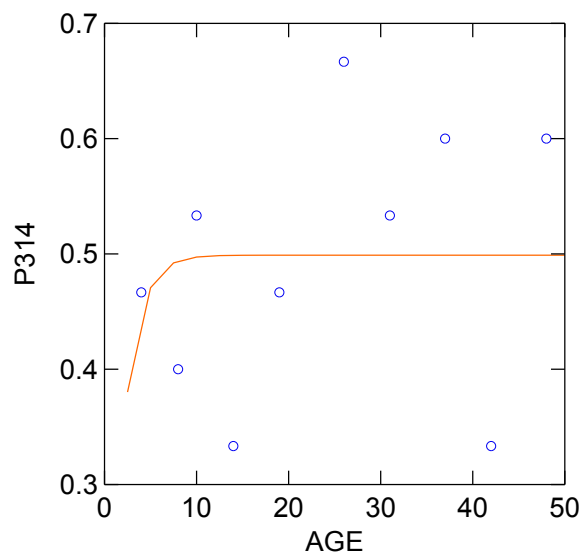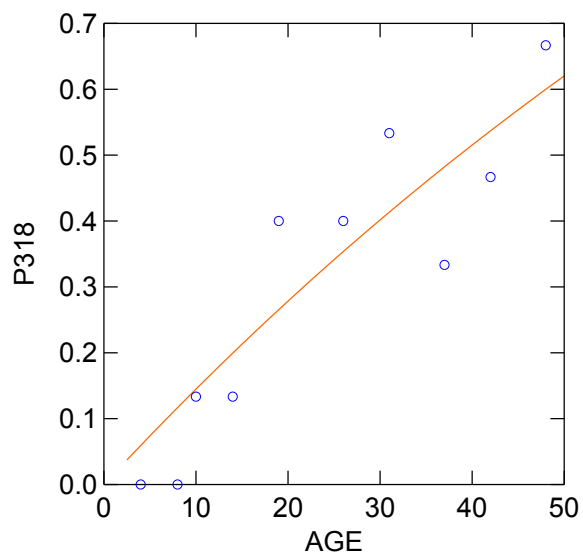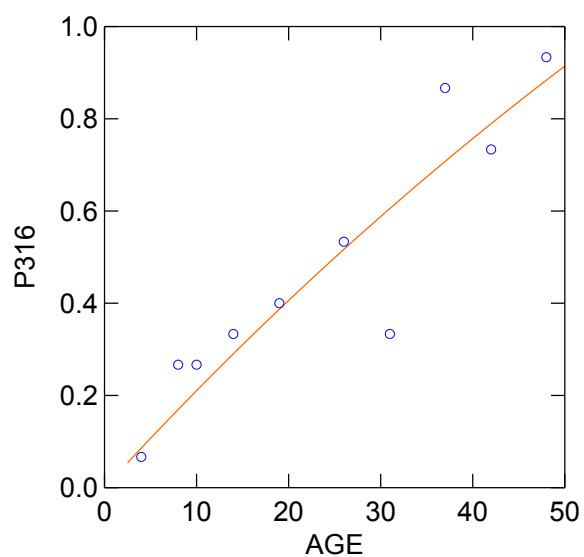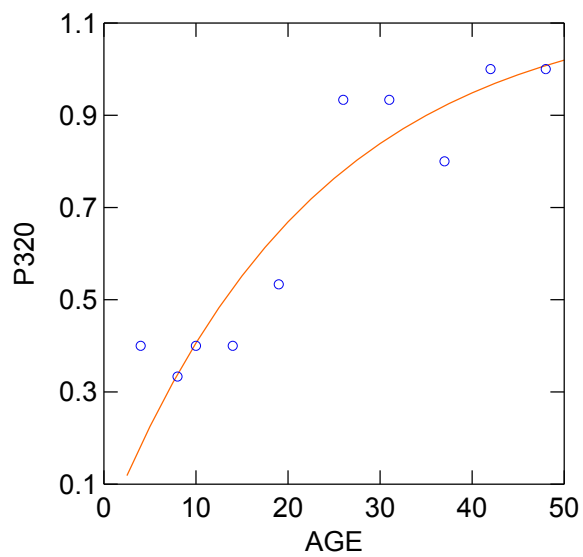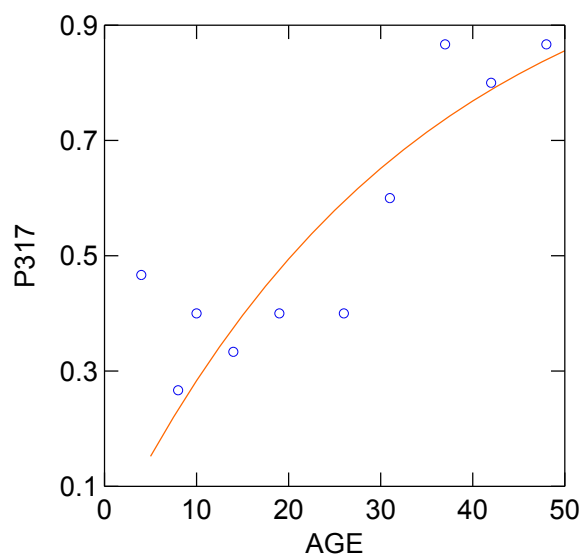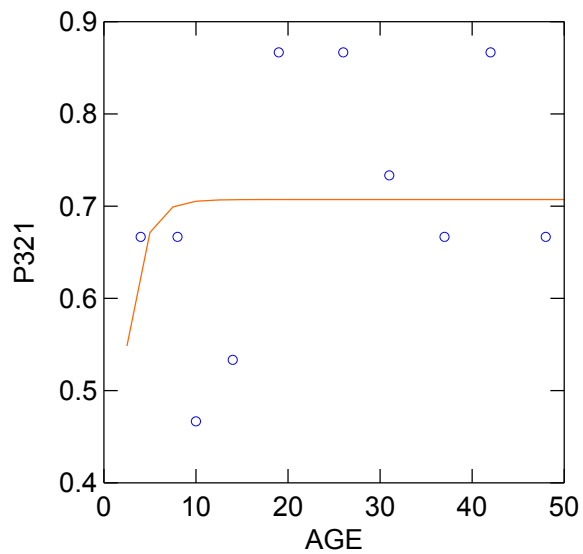

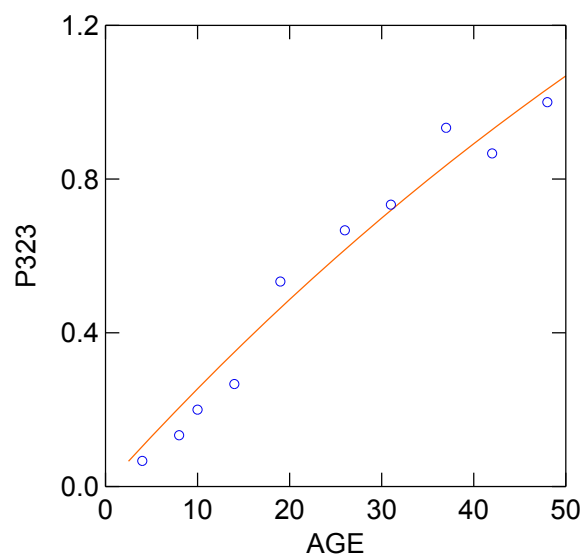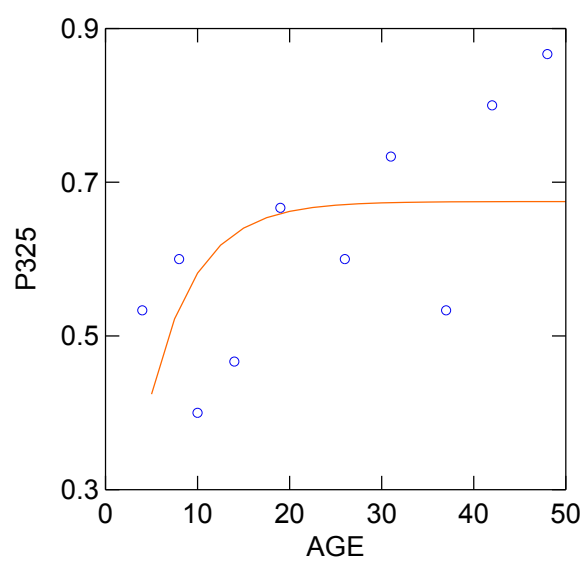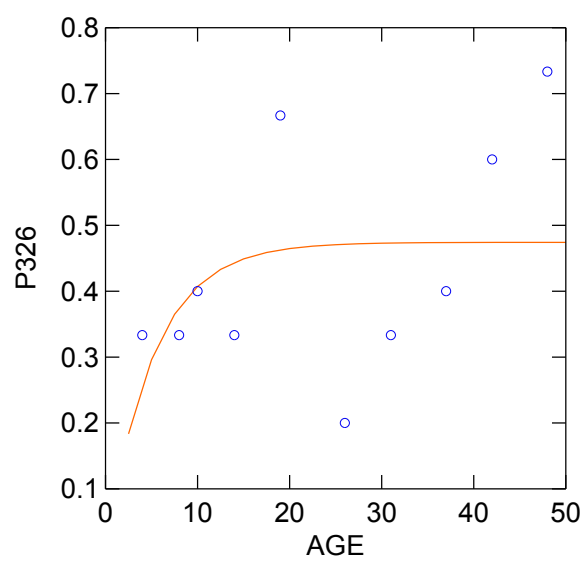

Supplement: Supplementary file 1 — 10.1186/s12936-016-1393-4 Age-fitted seroprevalence plots for seroconversion and reversion calculations. The average seroprevalence for each age group is plotted for the seroreactive Plasmodium proteins, denoted as P1 through P326. Their corresponding gene ID and protein description are found in Additional file 4. [file 12936_2016_1393_MOESM1_ESM.pdf]
